# MXene‐Integrated Composites for Biomedical Applications: Synthesis, Cancer Diagnosis, and Emerging Frontiers

**DOI:** 10.1002/smsc.202400492

**Published:** 2025-02-17

**Authors:** Aryan Saxena, Akshayat Tyagi, Sushipra Vats, Ishita Gupta, Akhil Gupta, Raminder Kaur, Saurabh Kr Tiwary, Ahmed A. Elzatahry, Maninderjeet Singh, Alamgir Karim

**Affiliations:** ^1^ Department of Applied Chemistry Delhi Technological University Delhi 110042 India; ^2^ Department of Chemical and Biomolecular Engineering (Materials Science and Engineering Program) University of Houston TX 77204 USA; ^3^ Department of Chemical and Biomolecular Engineering University of Houston TX 77204 USA

**Keywords:** 2D materials, biodegradability, cancer therapy, drugs, MXene synthesis, photoacoustic imaging photothermal therapy and photodynamic therapy

## Abstract

MXenes, a novel class of two‐dimensional (2D) transition metal carbides, carbonitrides, and nitrides, have gained significant attention in biomedicine. They are synthesized via two main approaches: top‐down etching of MAX (here, M represents an early transition metal, A represents an element belonging to the A‐group, and X represents N or C) phase precursors and bottom‐up chemical reduction of metal oxides. While the MAX phase method requires high temperatures, influencing morphology and posing safety concerns, alternative non‐MAX synthesis routes are emerging. Due to their unique physical and chemical properties, MXene‐based composites hold great promise in regenerative medicine, biosensing, and cancer therapy. However, challenges related to their stability, controlled drug release, and biodegradability remain. This review explores advancements in MXene synthesis, emphasizing non‐MAX phase fabrication and biomedical applications. Notably, MXene‐based composites have shown remarkable potential in cancer treatment, particularly in photothermal and photodynamic therapy. Their mechanisms, advantages, and limitations are discussed, along with future prospects and challenges in clinical translation. The development of MXene composites offers new avenues for innovative cancer therapies, paving the way for improved treatment strategies.

## Graphical abstract

MXenes are two‐dimensional materials with unique properties like high conductivity and potential for surface functionalization. This review focuses on their synthesis through top‐down and bottom‐up methods and their biomedical applications, including photodynamic therapy and photothermal therapy. The challenges and future directions for advancing MXenes in biomedical fields are also discussed.

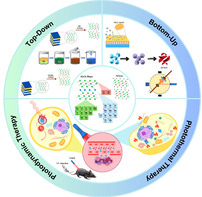

## 1 Introduction

The introduction of 2D materials has revived the advancement of illness therapy and biomedical research. Compared to single‐element graphene, 2D nanomaterials' distinctive properties offer higher performance, flexibility, and compatibility.^[^ ^1^ ^]^ These qualities were acknowledged after they were awarded the 2010 Nobel Prize.^[^ ^2^ ^]^ In addition, they differ from other kinds of nanomaterials in properties like physical, chemical, etc., due to their ultrathin thickness and 2D morphological structure.^[^ ^3^ ^]^ In 2004, Novoselov et al. made a key discovery by utilizing mechanical exfoliation to uncover the distinct transport properties of individual graphene sheets.^[^ ^4^ ^]^ Due to their extraordinary qualities in building, electrochemistry, and optoelectronics, 2‐dimensional materials like graphene are gaining attention in study. This has made it possible for cutting‐edge innovations to be made in conventional equipment like sensors,^[^ ^5^ ^]^ energy converters, and storage appliances.^[^ ^6^ ^]^ Researchers have realized the possibility of varying the atomic layers in the materials that are layered to generate novel features as a result of their study of 2D materials. Since it was discovered in the year 2011, the transition metal carbide (TMC) and nitride (MXene) have garnered significant recognition because of its unique composition, characterized by a layered structure comprising a nitrogen or carbon layer sandwiched between two transition metal layers.^[^ ^7^ ^]^ MXene is a potential material for industrial applications such as energy conversion and storage, sensors, optical devices, and environmental usages because of its excellent structural, physical, electrical, and magnetic characteristics.^[^ ^8^ ^]^ Due to its appealing features, single‐ and few‐layered MXene are the subject of current research rather than multilayered MXene.

A possible solution for biological applications has arisen in the form of MXenes.^[^ ^9^ ^]^ These compounds have the formula given as *Mn *+ 1*AXnTx* (where n could be from 1 to 3). Here, *M* stands for transition metals, *A* represents the main‐group elements, *X* represents nitrogen and/or carbon, *T* represents surface termination groups, and *x* stands for the no. of surface functionalities. MAX phases are 3D layered ternary nitrides/carbides, which are the source of these compounds. The selective etching process in MXene production can be influenced by factors like the extent and method of etching, the etching process duration, temperature, etc. Additionally, the specific MAX precursor used helps determine the etching selectivity. The choice of chemical etchant for exfoliation depends on the energy needed for breaking the M—A bond and the specific element being targeted.^[^ ^10^ ^]^

**Figure 1:**
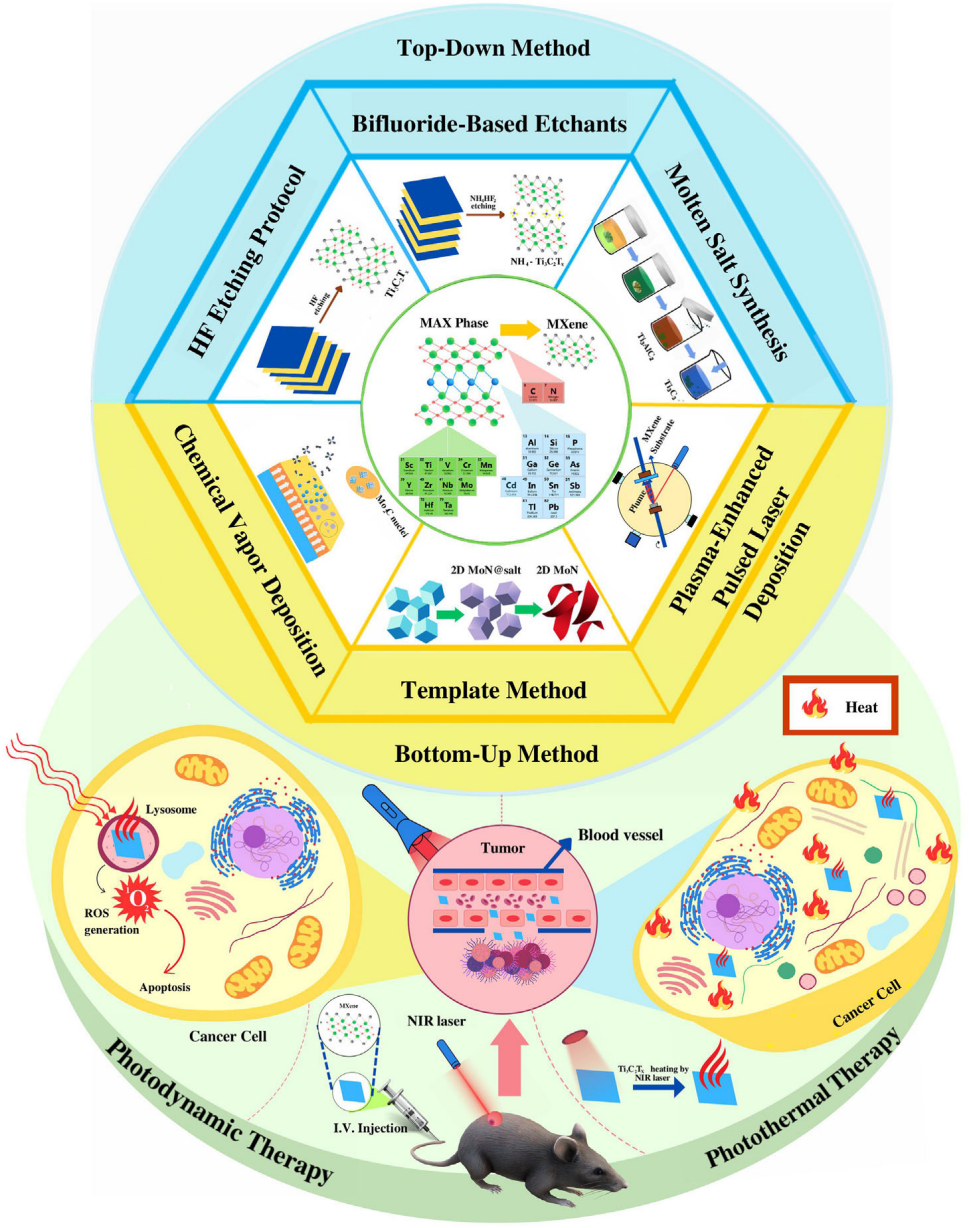
Schematic represents MXene synthesis methods and application in PDT and PTT cancer therapies.

The etching process and ambient factors can impact the selective etching procedure, which depends on the kind of MAX precursor employed. The chosen element type and the quantity of energy needed for breaking the M—A bond dictate the chemical etchant for the process of exfoliation (**Figure** **1**). Figure 1 illustrates the synthesis methods of MXenes and their biomedical applications, particularly in photodynamic therapy (PDT) and photothermal therapy (PTT). The synthesis is categorized into top‐down (e.g., HF etching, bifluoride‐based etching, molten salt synthesis) and bottom‐up (e.g., chemical vapor deposition (CVD), template methods, and plasma‐enhanced pulsed‐laser deposition (PEPLD)) approaches. These strategies enable the controlled exfoliation or formation of MXenes with tailored structures and surface terminations, such as O, OH, and F groups, which are essential for their functionalization and stability.

The figure also highlights the application of MXenes in PDT, where reactive oxygen species (ROS) are generated to induce cancer cell apoptosis, and in PTT, where MXenes achieve efficient photothermal conversion under near‐infrared (NIR) laser irradiation, leading to targeted tumor ablation. This visual representation emphasizes the critical role of synthesis strategies in enhancing MXene properties for advanced biomedical applications.

This review paper examines the biomedical applications of MXene‐based composites with a particular focus on regenerative medicine, infection therapy, and cancer treatments. It explores the integration of MXenes with other materials, such as nanosheets (NSs), nanoparticles (NPs), and polymers, and how these combinations influence the physicochemical properties of MXene compounds, including dispersion and photothermal activity. Unlike earlier reviews, which primarily focus on standalone MXene NSs and their general applications in energy storage, sensors, and environmental remediation, this review highlights the transformative potential of MXene‐based composites in the biomedical field. By emphasizing composite systems, this review addresses critical challenges like stability, biocompatibility, and biodegradability, often limiting factors for standalone MXenes in physiological environments.

Furthermore, this work bridges the synthesis of MXene composites and their practical biomedical applications. It provides insights into how advanced surface engineering strategies, such as PEGylation and NP functionalization, can overcome issues like aggregation and cytotoxicity. These strategies enhance the applicability of MXene composites in PTT, PDT, and targeted drug delivery. Importantly, this review underscores the synergistic effects achievable with MXene composites, as demonstrated in the combined PTT/PDT systems using MXene‐indocyanine green (ICG) hybrids, which exhibit superior therapeutic efficacy compared to individual components.

In addition to therapeutic applications, this review examines the role of MXene‐based composites in biosensing and wearable technologies, expanding the scope of their utility in the biomedical domain. This work contrasts with existing literature by addressing the challenges of scalability, stability, and clinical translation. It provides a forward‐looking perspective on the future of MXene‐based materials in biomedicine. It concludes by outlining the hurdles and opportunities the scientific community must address to develop MXene composites for biological applications further, offering a comprehensive roadmap for future research and practical deployment.

The potential of MXene‐integrated composite biomaterials in biomedical applications is examined in this review paper, emphasizing regenerative medicine, infection therapy, and cancer treatments. This article discusses the effects of combining MXenes with other substances, such as NSs, NPs, and polymers, on the physicochemical characteristics of MXene compounds, including dispersion and photothermal activities, in contrast to earlier reviews that concentrated solely on MXene NSs.^[^ ^11^ ^]^ The article also looks at how these composites might affect the effectiveness of MXene NSs in biomedical usages and their biocompatibility and biodegradability. The article also covers MXene‐based wearables and sensor development for biosensing applications. The essay concludes by outlining future directions and difficulties the scientific community must overcome to develop MXene‐based materials for biological applications further. This in‐depth analysis seeks to provide an invaluable roadmap for future progress in biomedicine, particularly about MXene‐based materials.

## 2 Synthesis and Modification of MXenes of Mxene

### 2.1 Synthesis of MXene

Recently, wet‐chemical etching methods that use hydrofluoric acid or etchants containing or producing hydrofluoric acid have been used to manufacture MXenes.^[^ ^12^, ^13^, ^14^ ^]^ The T_x_ in the formula *Mn *+ 1x*n*T_x_ represents the surface functions added during this process, such as O, F, or OH. Etching is still essential since mechanical exfoliation cannot produce MXenes in MAX phases because the A and M elements are tightly bound. Almost 20 members have already been generated in the MXene family, and more are anticipated to do so.

The MXene family possesses three different atomic structures, ranging from M_2_X to M_3_X_2_ and M_4_X_3_, which can be customized to meet specific material requirements.^[^ ^12^, ^15^, ^16^ ^]^ MXenes possess hydrophilic surfaces.^[^ ^12^ ^]^ and higher metallic conductivities (around 6000–8000 S cm^−1^).^[^ ^17^, ^18^ ^]^ Compared against other two‐dimensional (2D) materials like graphene, which makes them appropriate for uses like energy storage devices,^[^ ^19^, ^20^, ^21^, ^22^ ^]^ water desalination,^[^ ^23^ ^]^ catalysis,^[^ ^24^ ^]^ electromagnetic interference shielding,^[^ ^25^ ^]^ and producing transparent, conductive thin films.^[^ ^17^, ^18^, ^26^, ^27^ ^]^ As a result, MXenes have been attracting immense attention and hold significant potential in various technological fields.

The synthesis process for creating MXenes involves several stages, beginning with the aluminum layer etching from the parent MAX phase. This is followed by exfoliation utilizing a mixture of HF and HCl and the deposition of copper NPs. Once the etching is complete, the material must be washed to eliminate any residual acid and reaction products (salts) present and achieve a safe pH level of around 6. The washing procedure typically involves continuous centrifugation to separate the MXene, which has a multilayered structure, from the acidic solution, allowing for decantation of the acidic supernatant. Afterward, the multilayered flakes can be gathered through filtration with a vacuum and subsequently dried under vacuum conditions once the pH reaches ≈6.^[^ ^28^ ^]^

To create MXenes from the MAX phases, layered powders of MAX phases are combined with aq. HF and stirred at room temperature for a specific duration. This selective etching process removes the A layers of the MAX phase, causing the metallic bonds holding the MX layers together to be replaced by weaker surface termination bonds of hydroxyl, fluoride, or oxygen. The resulting mixture was separated from the solid with the help of centrifugation and filtration methods. The deionized water is used to wash the resulting mixture until the solution's pH falls within the 4–6 range, which yields a few‐layer (FL) MXene.^[^ ^29^ ^]^ Its important to note that MXene flakes may crumple when the pH of the MXene solution is lowered, as is the case with Ti_3_C_2_T_x_.^[^ ^30^ ^]^

While significant progress has been made in synthesizing MXenes and their composites, scalability and reproducibility remain key challenges. Developing greener, cost‐effective synthesis methods that avoid hazardous chemicals like HF and bifluorides is essential for industrial adoption. Moreover, innovative strategies such as one‐pot synthesis and in situ functionalization could streamline the fabrication of multifunctional MXenes, opening new avenues for biomedical applications.

#### 2.1.1 Top–Down Method of MXene Synthesis

Bulk crystal exfoliation directly through mechanical or chemical means is called the top‐down approach. In the synthesis of MXenes, liquid‐phase exfoliation is the preferred method because it tends to produce high‐yield and high‐efficiency ultrathin nanoscale. Creating nanoscale 2D MXenes from parent MAX‐phase ceramics involves two steps. First, multilayer stacked MXenes are delaminated through hydrofluoric acid (HF) etching. Next, the MXenes are disintegrated using either probe sonication breaking or intercalation by organic base molecules to produce a few layers or single‐layer MXenes. This method enables the fabrication of various types of MXenes with different morphologies, including size at the nanoscale and thickness at the atomic level (**Table** **1**).^[^ ^31^ ^]^

**Table 1:** Procedural steps involved in the fabrication of MXene via top‐down synthesis approach.

Method name	Etching	Washing	Drying	References
HF etching (Ti_3_C_2_T_x_ using HF)	Ti_3_AlC_2_ powder (0.5 g) is gradually added to 10 mL of HF solution. The reaction proceeds at 23 °C for 5–24 h, depending on the HF concentration (30, 10, 5 wt%).	Centrifuge the mixture and wash it with deionized water five times. Then, use vacuum‐assisted filtration with a PVDF membrane (0.22 μm pore size).	Vacuum dry at 80 °C for 24 h—final samples: 30 F‐Ti_3_C_2_T_x_, 10 F‐Ti_3_C_2_T_x_, 5 F‐Ti_3_C_2_T_x_.	[28]
Bifluoride etching (NH_4_HF_2_)	Ti_3_AlC_2_ powder (0.5 g) stirred with 10 mL of 2 m NH_4_HF_2_ solution at 23 °C for 1 day.	Centrifuge at 3500 rpm and wash with deionized water five times. Filter using a PVDF membrane. Repeat rinsing to remove impurities.	Dry in a vacuum oven at 120 °C for 24 h. Final sample: NH_4_‐Ti_3_C_2_T_x_.	[28]
Cl‐terminated Ti_3_C_2_T_x_ (molten salt)	Titanium, alumina, and graphite powders mixed with NaCl/KCl salts, shaped into pellets, and heated at 660 °C to form the Ti_3_AlC_2_ MAX phase.	Dissolve any remaining salts using an ammonium persulfate solution. Wash to remove Cu residues and other contaminants.	Vacuum filtration followed by drying at 80 °C for 12 h.	[189]
MXenes using molten salt synthesis	Ti_3_AlC_2_ MAX phase etched at 1300 °C. CuCl_2_ was added after lowering the temperature to 700 °C for Ti_3_C_2_T_x_ production.	Wash the product with ammonium persulfate to remove salts and contaminants.	Dry the filtered product in an oven at 80 °C for 12 h.	[189]
HF etching protocol/synthesis of Ti_3_C_2_T_x_ using HF as an etchant	Over five minutes, stirring with a Teflon magnetic bar, 0.5 g of Ti_3_AlC_2_ powder was gradually introduced to 10 mL of etchant.	Using centrifugation, each powder was washed five times individually, using a total of 1000 mL of deionized water. (5 min @ 3500 rpm per cycle). Ti_3_C_2_T_x_ sediments were vacuum‐assisted filtered over a polyvinyl difluoride (PVDF) filter membrane with a 0.22 m pore size before being washed once more with 1000 mL of deionized water (Durapore, Millipore).	MXene particles are vacuum‐dried at 80 °C for 24 h. The powders were given 30 F‐Ti_3_C_2_T_x_,10 F‐Ti_3_C_2_T_x_, and 5 F‐Ti_3_C_2_T_x_, respectively, for 30, 10, and 5 wt% HF.	[28]
	The reaction was then allowed to continue at room temperature (23 °C) for the following durations and concentrations of HF: 5 h for 30 wt%, 18 h for ten wt%, and 24 h for five wt%.			
Synthesis of Ti_3_C_2_T_x_ using a fluoride‐based etchant, NH_4_HF_2_	10 mL of 2 M NH_4_HF_2_ were combined with half a gram of Ti_3_AlC_2_ powder while continuously stirred. The reaction was then allowed for 1 day at room temperature (23 °C).	The combined produced was centrifuged at a rate of 3500 rpm. After this, they are washed with the deionized water (5 times per 5 min per cycle) following the Ti_3_C_2_T_x_ product's passage through a PVDF filter membrane, a 0.22 m pore size, washing was carried out using vacuum‐assisted filtration. The filtration system was then used to rinse the product with 1000 mL of deionized water.	The powder was dried in a vacuum oven for 24 h at 120 °C. NH_4_‐Ti_3_C_2_T_x_ is the MXene fabricated by this method.	[28]
Cl‐terminated Ti_3_C_2_T_x_ and Ti_2_CT_x_ MXenes using molten salt synthesis	Titanium, alumina, and graphite powders are combined with NaCl and KCl salts, shaped into pellets, and heated in a crucible containing a bed of chloride salt at ≈660 °C to produce the Ti_3_AlC_2_ MAX phase. The molten salt protects the reactants from oxidation by acting as a reactive medium and preventing them from directly touching the air. Etching at 1300 °C for 1‐h results in the Ti_3_AlC_2_ MAX phase. For producing Ti_3_C_2_T_x_ MXene, CuCl_2_ is added to the melted mixture only after lowering the crucible's temperature to 700 °C.	After cooling to room temperature, the samples are rinsed with deionized (DI) water and an ammonium persulfate (APS, (NH_4_)2S2O8) solution to facilitate the dissolution of any remaining solidified salts and to eliminate the Cu residue from the surface of the MXene particles.	The surface MXene materials formed using this molten salt preparation technique were F‐free and Cl‐containing. Vacuum filtration is used to collect the finished product, which is then dried for 12 h in an oven at 80 °C.	[189]

##### HF Etching Protocol

**Figure 2:**
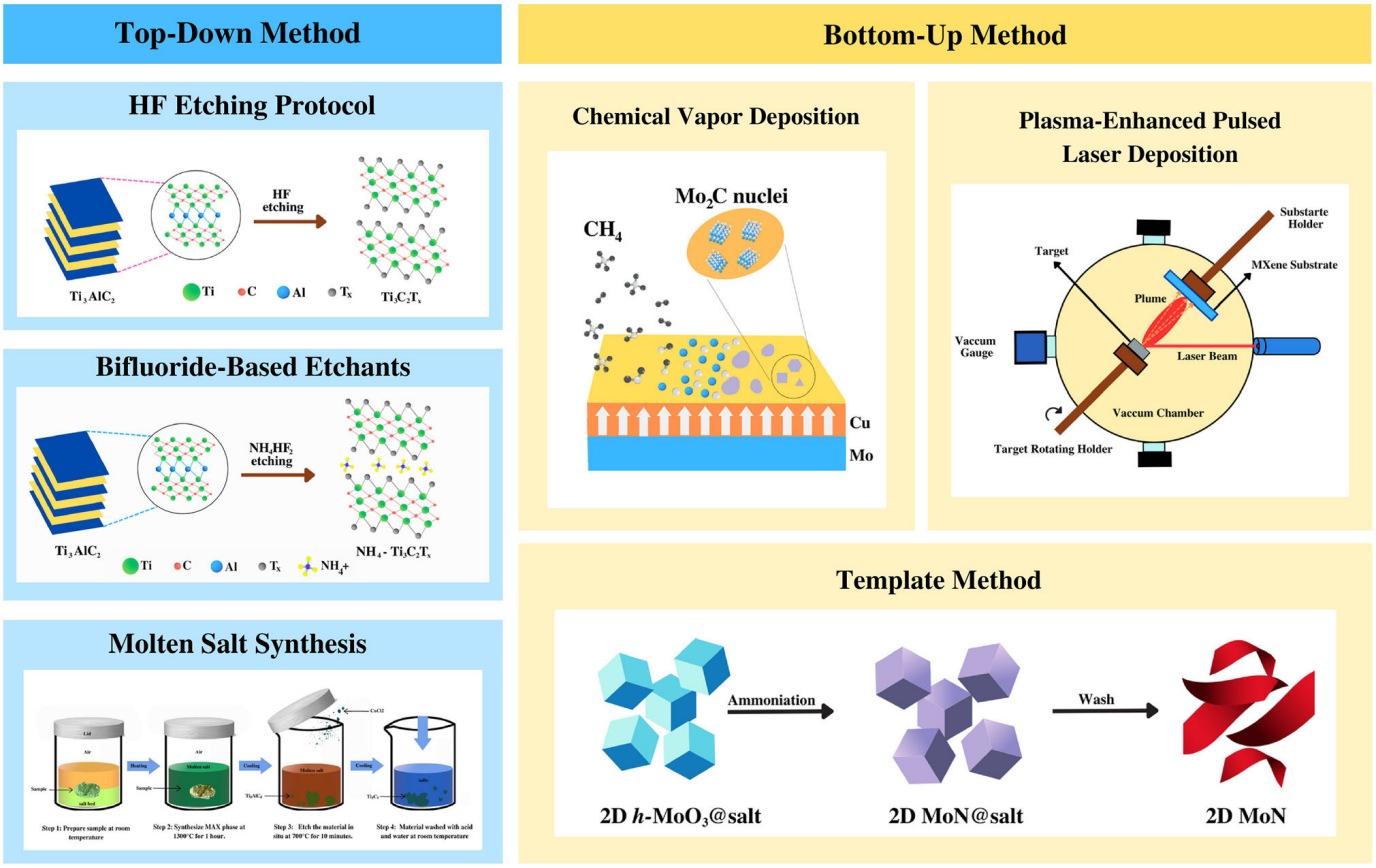
Top‐down and bottom‐up synthesis methods.

The results obtained from analyzing three different concentrations of HF (30, 10, and 5 wt%) indicate that eliminating Al could be attained using as little as 5 wt% HF. Additionally, when using a concentration of 30 wt% HF, a 5‐h etching period was found to be sufficient. The synthesis of Ti_3_C_2_T_x_ can be accomplished using the three different HF concentrations, as depicted in **Figure** **2**.

The synthesis of Ti_3_C_2_T_x_ MXene involves several steps. First, a Teflon magnetic bar is used to stir 0.5 g of powdered Ti_3_AlC_2_ into 10 mL of etchant over five minutes. To prevent uncontrolled bubbling from the exothermic reaction, additional Ti_3_AlC_2_ powder is steadily added. The reaction takes place at ambient temperature, employing distinct HF concentrations and different durations of 5, 18, and 24 h. These durations correspond to HF solutions of 30, 10, and 5 wt%, respectively. These durations are sufficient to achieve exhaustive etching. After etching, the powders are subjected to five wash cycles using 1000 mL of deionized water, each conducted for a brief period at 3500 rpm using centrifugation. The leftover Ti_3_C_2_T_x_ sediments are collected by the method of vacuum‐assisted filtration over a filter membrane of polyvinyl difluoride (PVDF) with a 0.22 μm filter porosity (Durapore, Millipore) and washed again with 1 L of distilled water before being collected.

MXene particles are vacuum dried at 80 °C for a time period of 24 h. The powders have been given the names 30F‐Ti_3_C_2_T_x_, 10F‐Ti_3_C_2_T_x_, and 5F‐Ti_3_C_2_T_x_, respectively, for 30, 10, and 5 wt% HF. All the three MXene powders have been kept in vacuum condition before being processed or subjected to further examination. As is clear from numerous research and reviews pertaining to MXenes, HF concentrated form has been used frequently as a preferred etchant to extract Al from Ti_3_AlC_2_ in a selective manner. However, employing diluted etchant with 5 wt% HF is a workaround for not utilizing excessively high HF concentrations. The alternative procedure involves creating etchants with 3–5 wt% HF in situ using fluoride salts like LiF or NH_4_HF_2_, or hydrogen fluoride.

According to studies on MXenes, concentrated HF has commonly been employed as a favored etchant to extract Al from Ti_3_AlC_2_ selectively. Therefore, a remedy for not using extremely high HF concentrations is diluted etchant with 5 wt% HF. The alternative procedure involves creating etchants with 3–5 wt% HF in situ using fluoride salts like LiF NH_4_HF_2_ or hydrogen fluoride.^[^ ^28^ ^]^

##### Bifluoride‐Based Etchants

Thin coatings of Ti_3_AlC_2_^[^ ^26^ ^]^ have been etched using salts of HF, like NH_4_HF_2_, at ambient temperature.^[^ ^32^ ^]^ For a couple of hours, Ti_3_AlC_2_ powder was etched for 5 days at 60 °C or room temperature.^[^ ^33^ ^]^ In this article, the delamination methods for MXene powders made with NH_4_HF_2_ are described along with the method's description (which is covered in more detail later). These are the steps for creating NH_4_HF_2_ etched Ti_3_C_2_T_x_:

In an experiment, 2 m NH_4_HF_2_ with 10 mL volume was continuously mixed with 0.5 g of Ti_3_AlC_2_ powder, then left to react at 23 °C for 24 h. The final mixture was centrifuged after that at 3500 rpm, followed by washing five times using deionized water for 5 min per cycle. The Ti_3_C_2_T_x_ sample was then filtered using a PVDF membrane filter with a pore size equal to 0.22 μm, and vacuum‐assisted filtration was used for washing. The filtered product was further rinsed with 1000 mm of deionized water. A vacuum oven was used at 120 °C for 24 h to dry the powder. The final MXene obtained from this process is known as NH_4_‐Ti_3_C_2_T_x_. This process ensures that the product is free from any impurities and is of high purity, making it suitable for further research and experimentation.

The absence of peaks of Ti_3_AlC_2_, such as no (002) at 9.5°, in the X‐ray diffraction (XRD) pattern tells about the Al selective removal. From 9.5° in the Ti_3_AlC_2_ predecessors, the basal plane peaks of NH_4_‐Ti_3_C_2_T_x_ are displaced to 8.5° and 7.15°, respectively, corresponding to d‐spacings of 10.35 and 12.37. The existence of constrained water molecules and (NH_4_^+^) ions in between MXene layers contributes to the increased d‐spacing after drying.^[^ ^26^, ^33^ ^]^ In contrast to HF‐etched Ti_3_C_2_T_x_, NH_4_‐Ti_3_C_2_T_x_ takes more time or vacuum drying at higher temperatures to eliminate the closely packed water molecules from the powder, as depicted in Figure 2.

#### 2.1.2 Molten Salt Synthesis/One‐Pot Synthesis Process

Here, the process for synthesizing MXene from elemental precursors using titanium aluminum carbide production, in situ etching, and the method of one molten salt pot is described. The role of molten salts as reaction media is to prevent the reactants from oxidizing throughout the high‐temperature fabrication process, hence allowing the production of MXenes in an ambient atmosphere without inert gas protection. This one‐pot synthetic approach, which involves a high‐temperature etching, takes about 10 min to produce Cl‐terminated Ti_2_CTx and Ti_3_C_2_T_x_ MXenes. This molten salt preparation technique produced MXene without surface F atoms but contained Cl atoms. When tested as an active material in the working electrode of a nonaqueous half‐cell, the MXene displayed a fast, reversible Li‐ion intercalation reaction.^[^ ^34^ ^]^

Ti_3_C_2_T_x_ MXene obtained by the one‐pot synthesis in an air atmosphere. Here, stoichiometric proportions of graphite, powdered titanium, and alumina are combined using chloride salts (KCL and NaCl), and the mixture is then crushed on a steel die to create a pellet. Finally, the pellet is placed in a crucible and covered with a bed of chloride salt. The crucible is heated in an air atmosphere using a muffle furnace. The KCl and NaCl mixture melts at a temperature of about 660 °C, acting as a reactive medium that prevents the reactants from coming into touch with air directly, preventing oxidation at high temperatures. By etching at 1300 °C for 1 h, the Ti_3_AlC_2_ MAX phase is created.

Powder XRD studies confirm the successful production of the Ti_3_AlC_2_ MAX phase at 1300 °C in molten salt. Only when the melts have cooled to 700 °C is CuCl_2_ introduced to enable the Ti_3_AlC_2_ MAX phase's in situ etching to create Ti_3_C_2_T_x_ MXene. According to a previous study, Ti_3_AlC_2_ MAX phase etching happens when Cu2+ ions are reduced into Cu and associated Al is oxidized into the vaporous AlCl_3_ phase, with a boiling point of 181 °C. Once the samples have been cooled to ambient temperature, they undergo a rinsing process using deionized water and a solution containing ammonium persulfate (APS, (NH_4_)2S2O8). This procedure dissolves the crystallized salts and removes any copper residue from the MXene particle's surface.^[^ ^34^ ^]^ Vacuum filtration is used to collect the finished product, which is then dried for 12 h in an oven at 80 °C.

#### 2.1.3 Bottom–Up Methods

The bottom‐up approach uses tiny organic or inorganic molecules to synthesize MXene. This method has an edge over the top‐down method as it facilitates the synthesis in a controlled manner with adequate size and surface terminations.^[^ ^35^ ^]^ The bottom‐up can be applied to prepare MXenes using CVD, atomic layer deposition, and top‐down fabrication methods.^[^ ^26^, ^36^, ^37^, ^38^, ^39^ ^]^

**Figure 3:**
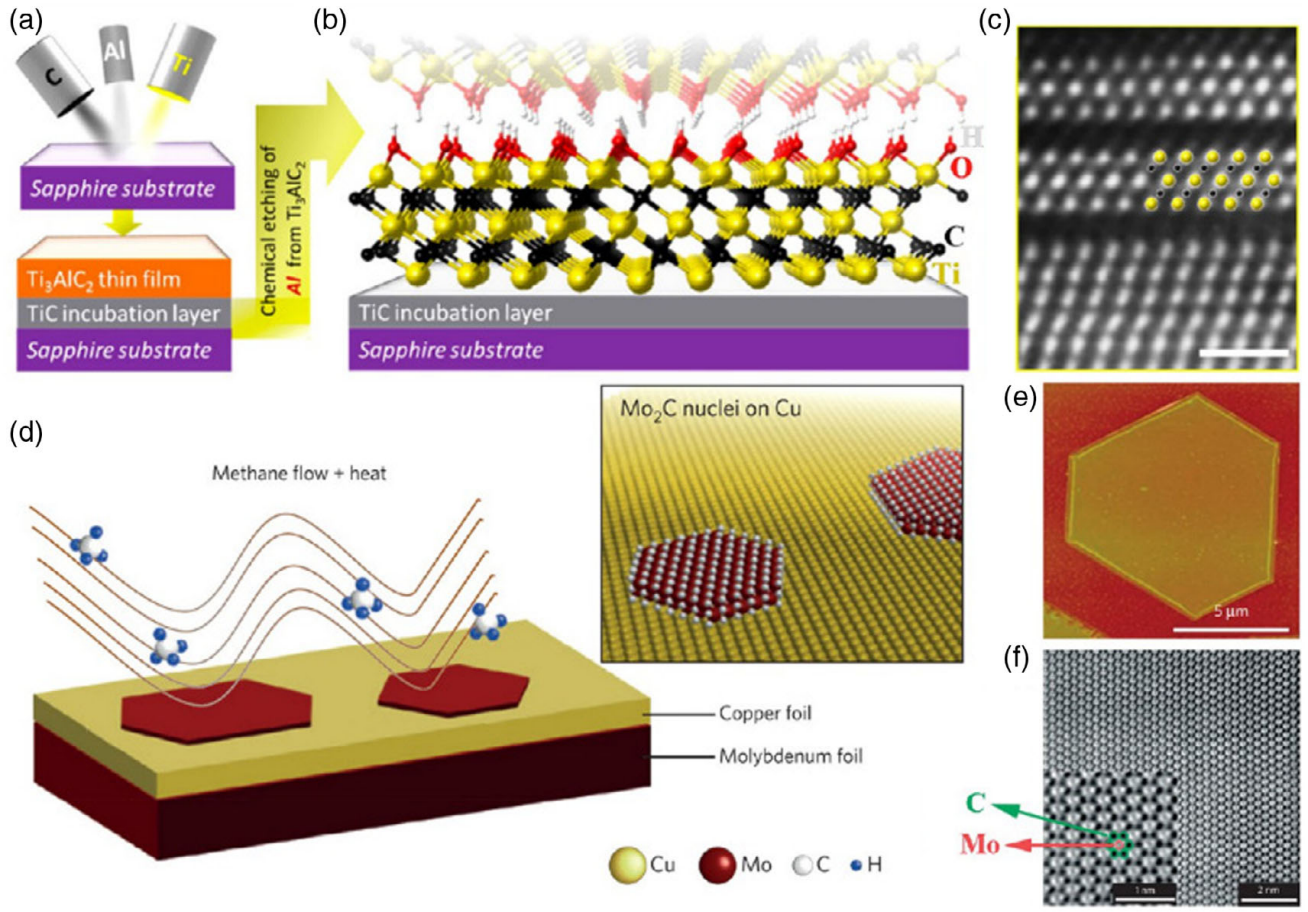
MXenes produced via bottom‐up techniques. Nuclear layer formation: a) Ti, Al, and C sputtering on a sapphire substrate are the steps needed to create a Ti_3_AlC_2_ MAX thin film, b) following selective etching of Al, a schematic of Ti_3_C_2_Tx, and c) obtained Ti_3_C_2_Tx's scanning transmission electron microscopy (STEM) image. CVD approach: d) the schematic of the synthesis process of the Mo_2_C, e) hexagonal ultrathin Mo_2_C crystal AFM image, and f) obtained Mo_2_C sheet's STEM image. (Panels (ac): Reprinted with permission.^[^ ^26^ ^]^ Copyright 2014, ACS. Panel (d): Reprinted with permission.^[^ ^36^ ^]^ Copyright 2015 Springer Nature. Panels (e,f): Reprinted with permission.^[^ ^37^ ^]^ Copyright 2015, Springer Nature. Copyright pending).

By using direct current magnetron sputtering to deposit Ti, C, and Al elemental targets on an insulating substrate of sapphire, Halim et al. created the Ti_3_AlC_2_ MAX thin film (**Figure** **3**a–c).^[^ ^26^ ^]^ Once the Al layer was precisely etched, a 1 by 1 cm^2^ Ti_3_C_2_ thin film (19 nm) was created. Around 90% of visible to infrared light was transmitted through the film, which aided in creating monolayer MXene thin films. Moreover, the physical vapor deposition method can make thin films in either the non‐MAX phase (Mo_2_Ga_2_C) or the MAX phase (Mo_2_GaC). A straightforward etching approach could also create thin epitaxial Mo_2_C films.^[^ ^40^, ^41^ ^]^

At a temperature exceeding 1085 °C (shown in Figure 3d–f), a Cu/Mo alloy surface was utilized to create high‐quality, 2D ultrathin —Mo_2_C material over a large area through CVD, which has been a groundbreaking technique for directly synthesizing MXene‐based materials.^[^ ^36^, ^37^ ^]^ Methane was employed as the carbon source during the CVD process to achieve this.

It was shown that altering the experimental conditions allows one to manipulate the dimensions and thickness of Mo_2_C crystals. It was revealed that lateral size and nucleation density rise with increased growth time and temperature, respectively. The same procedure could be employed for creating crystals of Mo in different shapes, like triangles, rectangles, hexagons, nonagons, octagons, and dodecagons.^[^ ^39^ ^]^ Jia et al. improved this strategy, and by using MoO_2_ NSs as templates and Mo sources, ultrathin —Mo_2_C NSs were quickly and effectively manufactured.^[^ ^42^ ^]^

Contrary to the 2D materials derived from various etchants, MXenes synthesized by CVD contain fewer flaws and no terminations, providing a platform to explore their inherent properties and the effect of the domain boundaries. The bottom‐up synthetic techniques should be researched further to fabricate other types of monolayered MXenes with diverse functionalities. Studies relevant to the inherent electrical and optical characteristics of MXenes will benefit from this.^[^ ^36^, ^43^, ^44^, ^45^ ^]^

The top‐down approach can produce MXenes in large quantities; however, using HF or LiF/HCl solutions during the synthesis results in flaws and an MXene surface ending with various functional groups.^[^ ^12^ ^]^ Furthermore, sonication or mechanical vibration causes MXene to shrink and produce more flaws. Studies focused on the inherent characteristics of 2D TMNs and TMCs have been impeded due to mechanical damage and chemical alterations to MXenes. The parent MAX phase with 3D lattice also restricts the species and MXenes structure. Moreover, every MXene created so far has a structure of the M_2_X, M_3_X_2_, or M_4_X_3_ kind.^[^ ^12^ ^]^ Furthermore, MX structure MXenes cannot be prepared with this method (such as NbN, NbC, MoN, MoC, etc.).

**Figure 4:**
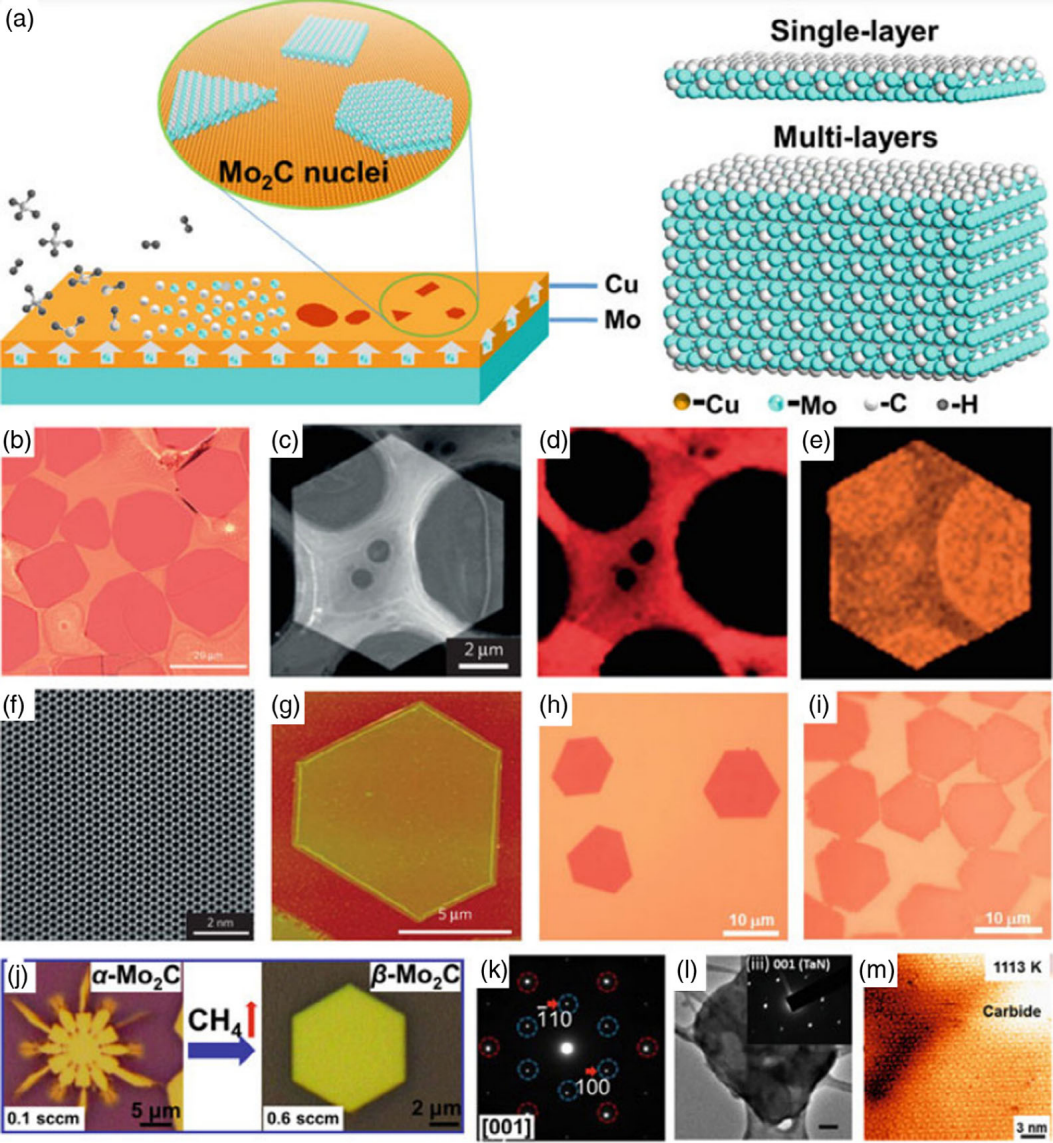
The transition metal nitrides (TMNs) and TMCs 2D thin films were produced using CVD. a) The Schematic diagram shows the formation process and the α‐Mo_2_C crystals’ atomic models with varying thicknesses. b) Ultrathin 2‐dimensional α‐Mo_2_C crystals on the growth substrate are shown in an optical image. c–e) High‐angle annular dark‐field scanning transmission electron microscopy (HAADF‐STEM) image with low‐magnification (c) and the C‐K (d) and MoK's associated EDS elemental mapping. e) The hexagonal 2‐dimensional α‐Mo_2_C crystal having the lines across through them. f) α‐Mo_2_C crystal's HAADF‐STEM image in c. g) Hexagonal ultrathin 2D α‐Mo_2_C crystal's AFM image (having a thickness equal to 8.2 nm). h,i) 2‐dimensional ultrathin WC (h) and TaC (i) crystals optical images. j) Optical images depicting the structure evolution of the Mo_2_C crystal with increment in the flow rate of CH_4_ of a fractal Mo_2_C crystal and a hexagonal α‐Mo_2_C crystal getting transferred over SiO_2_/Si substrates. k) Hexagonal Mo_2_C crystal's SAED pattern. l) 2D TaN crystal transmission electron microscopy (TEM) images at low magnification; the inset displays the SAED pattern. 100 nm wide scale bars are used. m) Periodic “flower‐like” ReC superstructure. Reproduced with permission.^[^ ^37^ ^]^ Springer Nature, panels (b–i) are reprinted. Panels (j) and (k) are adapted from Ref. [46], Institute of Physics. Reproduced with permission.^[^ ^47^ ^]^ Wiley‐VCH, Panel (l) is reprinted with permission,^[^ ^56^ ^]^ ACS copyright pending, panel (m) is reprinted).

Using selective area electron diffraction (SAED), it has been discovered that the crystal had an orthorhombic structure (—Mo_2_C). Energy‐dispersive X‐Ray spectroscopy (EDS) maps in **Figure** **4**c–e showed that the crystal had a consistent chemical makeup. The lack of flaws in Figure 4f indicates the material's superior crystalline quality. Atomic force microscopy (AFM) measurements were utilized to observe the crystals' slick and spotless surface. Notably, this CVD process can be deployed to create a variety of 2D ultrathin TMC crystals with high quality and various structural variations due to its adaptability.

For instance, by substituting W and Ta foil for Mo foil, respectively, Figure 4h,I illustrates the production of ultrathin 2D hexagonal WC and cubic TaC crystals—the identical procedure examined by Xu et al.^[^ ^37^ ^]^ It was used by Geng et al.^[^ ^46^ ^]^ to create 2D Mo_2_C crystals. They conducted their research using copper foil with a minimum thickness of 50 m, at a higher temperature of 1100 °C, and different CH_4_ concentrations (CH_4_ 0.1–0.4 sccm, H_2_ 200 sccm).

The shape of Mo_2_C crystals changed depending on the concentration of CH_4_ observed by the researchers. At a flow rate of 0.1 cm, the resulting crystals had a fractal shape. However, with an increased methane flow rate, the lattice structure transformed to hexagonal from fractals, as illustrated in Figure 4j. The hexagonal Mo_2_C crystal had a more varied crystalline structure than other crystals (as shown in Figure 4k). The most prevalent shapes were rectangular and pentagonal when the methane flow rates were 0.3 and 0.4 cm, respectively. The thickness of the copper foil increased as the thickness of the Mo_2_C crystal decreased.

This is because a thicker layer of copper allows more time for Mo to diffuse, leading to a more minor degree of separation on the surface of the liquid copper.

Wang et al.^[^ ^47^ ^]^ and Xu et al. created a procedure to produce extremely thin TaN using Cu foil/Ta foil as the growth substrate, NH3 as the nitrogen source, and a temperature of 1077 C. This temperature is below the melting point of copper (Cu). This procedure was based on earlier research by Xu et al.^[^ ^37^ ^]^ Copper foil can keep copper in its solid state during the CVD process. At higher temperatures, Ta atoms in the Cu lattice tended to group rather than be evenly dispersed. Lower temperatures are, therefore, preferred for better control over the formation of these crystals.^[^ ^47^ ^]^

##### Template Method

The template method is a versatile approach for synthesizing 2D TMCs and TMNs using TMOs as precursors. In this method, TMOs are templates that undergo specific chemical reactions, such as carbonization or ammoniation, to produce the desired 2D materials. Xiao et al.^[^ ^48^ ^]^ demonstrated using the salt‐templated method, initially developed for 2D TMO synthesis, to fabricate 2D TMNs. In their study, the salt templates facilitated the formation of uniform, thin layers of TMNs, ensuring better control over the morphology and size of the final material. By varying the temperature and reaction atmosphere (e.g., introducing carbon or ammonia gas), the composition and structure of the TMNs could be precisely tailored.

Additionally, the template method offers several advantages: 1) Structural control: It enables the synthesizing of well‐defined 2D structures with high uniformity and tunable thickness. 2) Scalability: The salt‐templated approach can be adapted for large‐scale production, making it more feasible for industrial applications. 3) Versatility: This method allows the synthesis of various 2D materials, including carbides, nitrides, and hybrid systems, by carefully selecting the precursor templates and reaction conditions.

Recent advancements have also combined the template method with molten salt synthesis, where molten salts act as both a template and a reactive medium, further improving the synthesis efficiency and material quality—for instance, Jin et al.^[^ ^49^ ^]^ Successfully synthesized multilayered and few‐layered 2D TMNs and TMCs using molten salts achieve materials with enhanced electronic and catalytic properties—similarly, Xu et al.^[^ ^50^ ^]^ demonstrated using TMO templates to create hybrid systems for catalysis, energy storage, and biomedical engineering applications. Thus, the template method provides a practical and flexible strategy for fabricating 2D MXene‐like materials, expanding their potential applications in diverse fields.

Transition metal oxides (TMOs) are needed for the template approach to fabricate 2D TMCs and TMNs.^[^ ^42^, ^48^, ^51^ ^]^ The template is carbonized or ammoniated to create the TMOs. Xiao et al. used the salt‐templated method of 2D TMO synthesis as previously described^[^ ^48^ ^]^ and evaluated the manufacture of 2D TMNs using salt templates.

##### Plasma‐Enhanced Pulsed Laser Deposition

**Figure 5:**
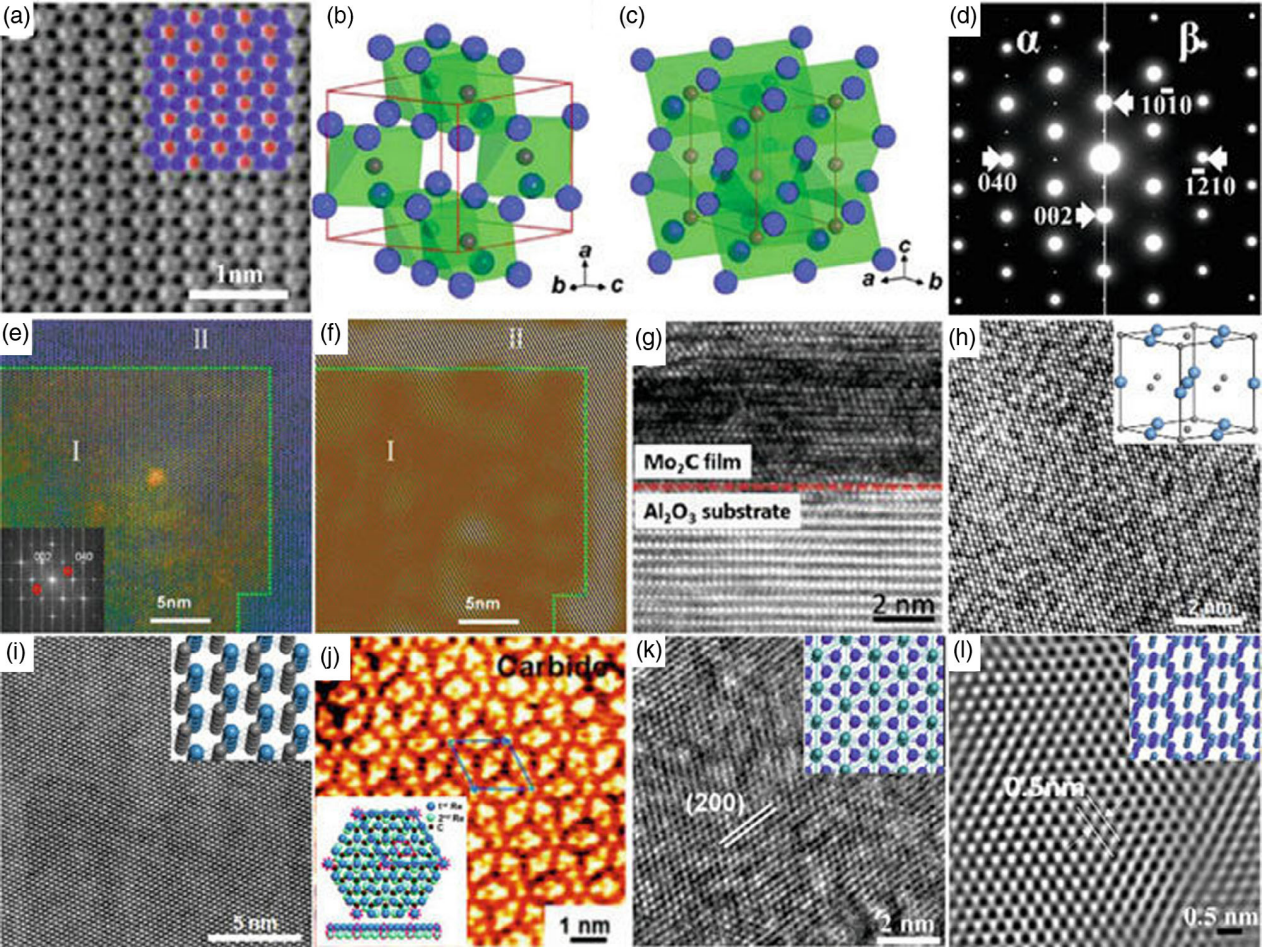
The crystalline nature characterizes the 2D structure of TMNs and TMCs produced using a bottom‐up method. a) An ultrathin α‐Mo_2_C's BF‐STEM image crystal viewed from the [100] direction. b,c) Atomic models of the unit cells of α‐Mo_2_C and β‐Mo_2_C, respectively. d) ED patterns of ultrathin α‐Mo_2_C and β‐Mo_2_C. e,f) The ultrathin Mo_2_C crystal is depicted in two forms: through a HAADF‐STEM image and its corresponding image obtained from the inverse Fourier transform. g) High‐resolution transmission electron microscopy (HRTEM) image of a thin Mo_2_C film, face‐centered cubic (FCC), and epitaxially grown over a sapphire substrate. h) Image of [111]‐oriented HRTEM of a cubic ultrathin TaC crystal, and the inset depicts the TaC unit cell's atomic model. i) Hexagonal ultrathin WC crystal [0001]‐oriented HRTEM image and the inset is shown by the WC atomic model. j) ReC's scanning tunneling microscopy (STM) image on a Re substrate is presented, and the of ReC's atomic structure is showcased by the inset, which consists of a single Re and C atom within a rhombic unit cell. k,l) HRTEM images, oriented along the [0001] direction, are shown for hexagonal ultrathin MoN and TaN crystals, respectively. The insets in these images depict the corresponding atomic models. (Reproduced with permission.^[^ ^39^ ^]^ Copyright 2016, ACS, panel a is reprinted. Reproduced with permission.^[^ ^52^ ^]^ Copyright 2017, Royal Society of Chemistry, panels (b–f) have been adapted. Reproduced with permission.^[^ ^54^ ^]^ Copyright 2017, Royal Society of Chemistry, panel (g) is reprinted. Reproduced with permission.^[^ ^37^ ^]^ Copyright 2015, Springer Nature, the HRTEM images shown in panels (h,i) have been adapted. Reproduced with permission.^[^ ^56^ ^]^ Copyright 2017, ACS, the STM image in panel (j) is reprinted. Reproduced with permission.^[^ ^48^ ^]^ Copyright 2017, ACS, the HRTEM image shown in panel (l) has been adapted. Reproduced with permission.^[^ ^47^ ^]^ Copyright 2017, Wiley‐VCH copyright pending, the HRTEM image shown in panel (k) has been adapted).

**Figure 6:**
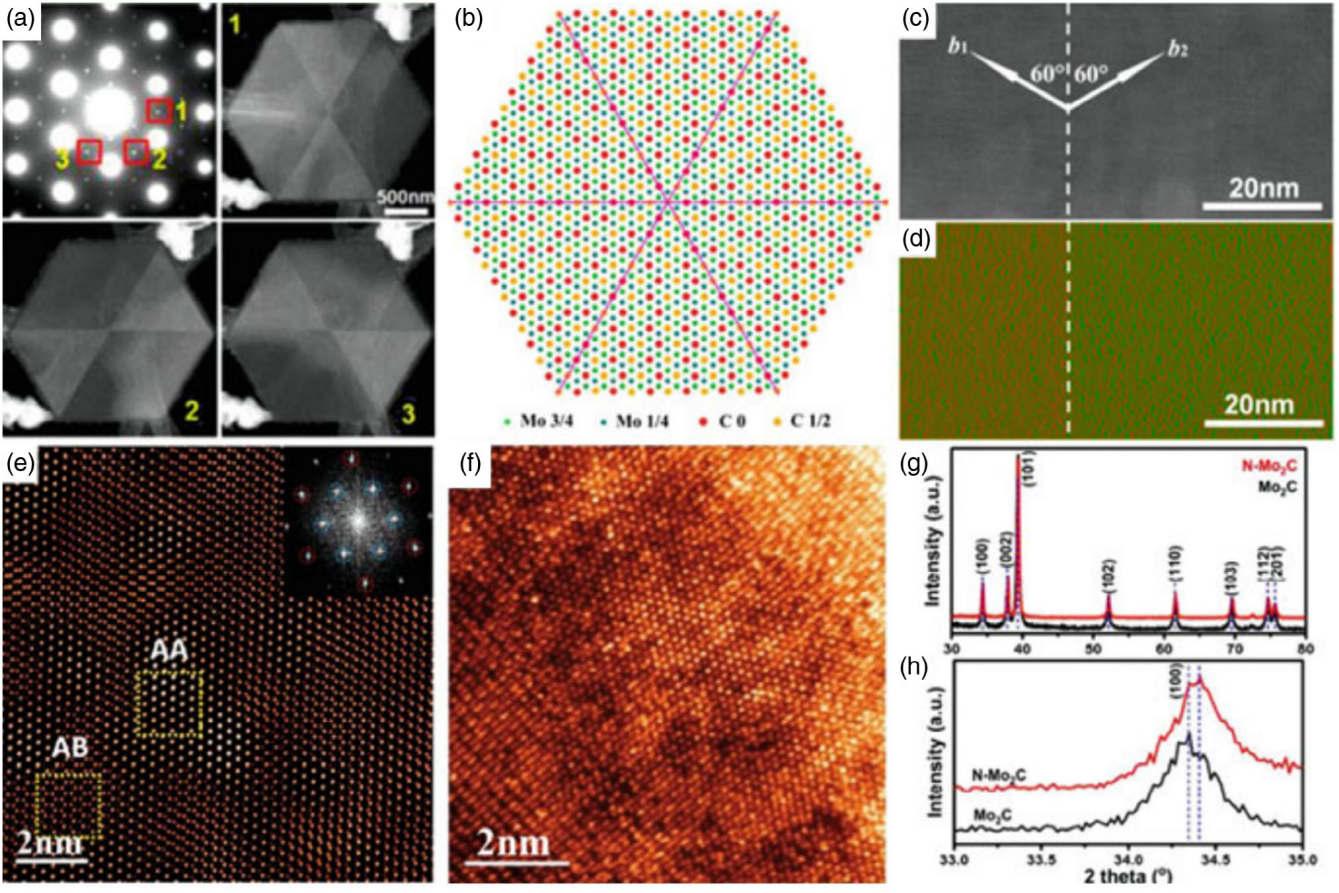
Defect structure of the bottom‐up synthesis‐produced 2‐dimensional TMCs and TMNs. a) Ultrathin α‐Mo_2_C crystal, ED pattern, and DF‐TEM pictures. The DF‐TEM pictures denoted by numbers 1–3 have been attained by choosing the ED spots denoted by the yellow numbers 1–3 in the ED pattern, which were selected from the crystal's center. b) Atomic representation of the hexagonal α‐Mo_2_C crystal demonstrating symmetric carbon distribution concerning domain boundary. c,d) HAADF‐STEM picture (c) and the appropriate mapping of shear strain (d) at the incredibly narrow α‐Mo_2_C domain border. The white dot line indicates the location of the domain boundary. e) An A and AB stacking order is visible in the 2Dβ‐Mo_2_C crystal in this ADF‐STEM image. f) 2D β‐Mo_2_C crystal stacking error. g) 2D β‐Mo_2_C and N‐doped β‐Mo_2_C crystal XRD patterns. h) The g local magnified XRD patterns were obtained between 33° and 35°. (Reproduced with permission.^[^ ^39^ ^]^ Copyright 2016, ACS, panels (a–d) are reprinted with permission.^[^ ^46^ ^]^ Copyright 2016, Institute of Physics, panel (e) is adapted with permission.^[^ ^38^ ^]^ Copyright 2017, Wiley‐VCH, panel (f) is reprinted with permission.^[^ ^42^ ^]^ Copyright 2017, ACS copyright pending, panels (g) and (h) are reprinted).

**Figure** **5**a depicts the image of the aberration‐corrected BF‐STEM of the ultra‐thin Mo_2_C crystals. The Mo six rings, which resemble graphene, and the carbon atoms positioned in their center are visible in this photograph.^[^ ^39^ ^]^ β‐phase anticipated structure is the same as α‐phase (**Figure** **6**b,c). Yet, in β—Mo_2_C, the carbon atoms are randomly scattered, whereas in α—Mo_2_C, they are orderly grouped in Mo octahedrons. As a result, the strong hexagonal symmetry of the Mo sublattice of α—Mo_2_C somewhat gives way to orthorhombic symmetry.

Even though the Mo hexagonal close‐packed (HCP) lattice's distortion in —Mo_2_C is too tiny for the naked eye to see, reciprocal space can be used to locate it. The contrast in electron diffraction (ED) between the α‐ and β—Mo_2_C is seen in Figure 5d, and it can be observed that in the α‐phase, superstructure ED spots are visible.^[^ ^52^ ^]^ It was inferred that α—Mo_2_C can be transformed from β—Mo_2_C. During a phase transition, carbon atoms become ordered due to cooling.^[^ ^53^ ^]^ However, it has been inferred by researchers such as Liu et al.^[^ ^52^ ^]^ The electron beam irradiation (EBI) could lead to the transition from α to β phase in two‐dimensional Mo_2_C crystals. In the presence of EBI, the carbon atoms in an ultrathin α—Mo_2_C crystal move from a zigzag to a random configuration. In contrast, the distorted HCP configuration is relaxed for Mo atoms to a rigorous configuration. This change from α to β phase can be seen in Figure 6f, where only area I of the crystal has been subjected to a high‐dose EBI. The advanced aberration‐corrected STEM use in EBI can also provide a way to create lateral junctions of 2‐dimensional superconducting α/β Mo_2_C having a sharp interface, as well as a platform for investigating vortex physics and the catalytic synergistic effects at the interface between phases—α and β. In addition, Zhang et al.^[^ ^54^ ^]^ have described how PEPLD can synthesize Mo_2_C thin films with an FCC structure.

The Mo atomic planes’ interplanar spacing in the three structures is nearly identical. Although the space group of FCC—Mo_2_C has been determined to be Fm3m with a lattice parameter of 0.415 nm (Figure 6g), the arrangement of carbon atoms within the structure remains uncertain. Through the use of a metal immiscibility route, 2‐dimensional TaC crystals with a rock salt structure were synthesized by Xu et al.^[^ ^37^ ^]^ and Wang et al.^[^ ^47^ ^]^ where the latter researchers noted that the crystals’ morphology suggested normal growth with a pyramidal morphology (Figure 6h). In CVD‐fabricated 2D WC crystals, the tungsten atoms are organized in an AA stacking pattern and form trigonal prismatic structures that can accommodate carbon atoms at their centers (as shown in Figure 6i). This differs from the AB‐stacking arrangement of metal atoms observed in α‐ and β—Mo_2_C. Fermions consisting of three components have been recently discovered in WC‐structured crystals.^[^ ^55^ ^]^

ReC was synthesized on a Re substrate in the study conducted by Qi et al.^[^ ^56^ ^]^ Although ReC has the same planar structure as WC, the Re and carbon atoms in ReC are arranged in a coplanar 2D structure (Figure 6j). So far, two types of 2‐dimensional TMNs, MoN and TaN, have been produced using a bottom‐up synthesis methodology. The resulting 2‐dimensional MoN crystals, obtained through ammoniation of MoO_3_,^[^ ^48^, ^51^ ^]^ exhibit a hexagonal structure with a space group of P63mc. The molybdenum skeleton has an alternating alignment with slightly misaligned atomic columns. N atoms have been located in interstitial sites in a trigonal prismatic arrangement around the Mo atoms. Nevertheless, the nitrogen nuclear layers perpendicular to the [0001] direction are not situated within the expected coincident trigonal prisms (Figure 6k).

The lattice dimensions of the 2D TaN crystals formed are 0.5196 nm for an as well as b and 0.2911 nm for c, with a space group of P6/mmm (Figure 6l).^[^ ^47^ ^]^

According to first‐principle calculations, the interstitial carbon atoms of the α—Mo_2_C lattice in the octahedrons are somewhat off‐center along the *b*‐direction. Yet, the same HCP molybdenum atom sublattice is connected to three identical off‐center carbon atom arrangements.^[^ ^39^ ^]^ Topological α—Mo_2_C domains may develop if identical carbon atom arrangements exist within the same sublattice of Mo atoms. Liu et al.^[^ ^39^ ^]^ stated that 2D α—Mo_2_C crystals with shapes characterized by three, six, nine, and twelve sides consist of several unique crystalline regions that exhibit domain structure and have delineated linear boundaries. Rectangular and octagonal 2D α—Mo_2_C crystals are single crystals.

For instance, the diagonal domains of the α—Mo_2_C NS, which is formed of sixfold rotationally symmetric domains, have the identical carbon atom structure as that in Figure 5a. The symmetric arrangement of atomic arrangement of carbon w.r.t the domain boundary has the lowest energy, according to first‐principles calculations (Figure 5b). The distorted orientations of the HCP Mo sublattice differ because the asymmetric directions of the carbon atoms in neighboring segments differ. As a result, the domain boundary exhibits a slight shear strain (Figure 5c,d). However, the creation of localized phase‐slip events in the boundary causes a considerable impact on the superconductivity.^[^ ^39^ ^]^

Mo atoms in —Mo_2_C should fundamentally be stacked in the “ABAB” configuration, according to Geng et al.^[^ ^38^ ^]^ there were two kinds of Mo stacking order, AA and AB, observed at the narrower portion of the hexagonal‐shaped β—Mo_2_C layer (Figure 5e). They proposed that the presence of different stacking configurations was likely due to partial growth of Mo_2_C at the edge and the comparable energy of formation for the two polytypes. Additionally, Geng et al.'s^[^ ^38^ ^]^ observation of a stacking flaw in the β—Mo_2_C NS. Additional Mo atoms can be seen in the middle of the projected Mo six rings, as seen in Figure 5f.

That is, in addition to the “A” and “B” layers, there is a Mo atomic plane of the local “C” layer. Jia et al. have studied that the C‐atoms substitution with N‐atoms in 2D—Mo_2_C resulted in the formation of N‐doped —Mo_2_C crystals, leading to a reduction in the crystal lattice spacing ratio a/b in β—Mo_2_C crystals (Figure 5g,h).^[^ ^57^ ^]^

#### 2.1.4 MXene Synthesis from Non‐MAX Phase

Posttreatment, any remaining In particles which do not undergo the reaction were eliminated by drenching the resulting rock‐like substance in concentrated HCl (37%) for a period of 24 h. Afterward, the solution underwent filtration and was subsequently subjected to multiple washes with distilled water through centrifugation until reaching a pH level of around 7. The solution was dried for 20 h at 100 °C in a hot air oven to produce the Mo‐In‐C non‐MAX powder. To convert the parent Mo‐In‐C into Mo_2_CTx, the parent material was carefully etched for 3–5 h in conc. H_3_PO_4_ (85%, Sigma Aldrich; 500 mg/50 mL). Centrifugation at 4000 rpm was used to repeatedly wash the solution with distilled water until the pH reached about 7. After being ultrasonically exfoliated for 3–5 h, the solution was centrifuged to get the Mo_2_CTx powder, which was subsequently dried in a hot air oven.

The prepared MXene exhibited both multilayered and monolayered morphologies, with the difference being attributed to the duration of exfoliation. The use of an eco‐friendly methodology for the fabrication of single and multilayered Mo_2_CTx from non‐MAX phases shows promise for electrochemical applications.^[^ ^58^ ^]^

Posttreatment, any remaining In particles that did not undergo the reaction were eliminated by drenching the resulting rock‐like substance in concentrated HCl (37%) for 24 h. Afterward, the solution underwent filtration and was subjected to multiple washes with distilled water through centrifugation until reaching a pH level of around 7. The solution was dried for 20 h at 100 °C in a hot air oven to produce the Mo‐In‐C non‐MAX powder. To convert the parent Mo‐In‐C into Mo_2_CTx, the parent material was carefully etched for 3–5 h in conc. H_3_PO_4_ (85%, Sigma Aldrich; 500 mg/50 mL). Centrifugation at 4000 rpm was used to repeatedly wash the solution with distilled water until the pH reached about 7. After being ultrasonically exfoliated for 3–5 h, the solution was centrifuged to get the Mo_2_CTx powder, which was subsequently dried in a hot air oven.^[^ ^58^ ^]^

#### 2.1.5 Synthesis of MXene‐Integrated Composites

The synthesis of MXene‐integrated composites is a critical area of research, as these composites often exhibit enhanced multifunctional properties compared to standalone MXenes. MXenes are typically combined with polymers, NPs, or other 2D materials through various synthesis strategies, including physical blending, in situ growth, and chemical crosslinking. These techniques improve the stability and biocompatibility of MXenes and provide tunable properties for biomedical applications such as drug delivery, imaging, and PTT.^[^ ^59^, ^60^, ^61^ ^]^

##### Physical Blending

MXene NSs are physically mixed with other materials, such as polymers or NPs, to form composites in this method. Deb and Jain blended Ti_3_C_2_ MXenes with polyvinyl alcohol (PVA), forming biocompatible and pH‐responsive composites demonstrating high photothermal conversion efficiency for tumor‐specific therapy.^[^ ^62^ ^]^ Similarly, He et al. reported that mixing Ti_3_C_2_ MXenes with hyaluronic acid enhanced their targeting efficiency, enabling drug delivery specifically to tumor sites while improving biocompatibility.^[^ ^63^ ^]^

Physical blending with mesoporous silica nanoparticles (MSNs) has also been explored. Wang et al. demonstrated that Ti_3_C_2_ MXene‐MSN composites exhibited pH‐sensitive drug release, achieving targeted delivery in acidic tumor microenvironments.^[^ ^64^ ^]^ Xu et al. developed Ti_2_N MSNs for combined PTT and drug delivery, showing improved stability and thermal efficiency.^[^ ^65^ ^]^

##### In Situ Growth

In situ growth involves synthesizing NPs or growing polymer networks directly on the surface of MXene NSs. Deb and Jain successfully grew Fe_3_O_4_ NPs in situ on Ti_3_C_2_ MXene surfaces, creating composites with enhanced magnetic resonance imaging (MRI) contrast capabilities and therapeutic potential for photothermal cancer therapy.^[^ ^62^ ^]^ Similarly, Yang et al. deposited Au NPs on MXene surfaces via in situ reduction, resulting in multifunctional composites for combined catalytic and photothermal applications.^[^ ^66^ ^]^

Polymers have also been synthesized in situ. Zhang et al. reported the in situ coating of polydopamine on Ti_3_C_2_ MXenes, which significantly improved their stability and drug‐carrying capacity for targeted delivery applications.^[^ ^67^ ^]^ Liang et al. developed MXene‐gelatin hydrogels via in situ polymerization, demonstrating excellent mechanical properties and wound‐healing potential.^[^ ^68^ ^]^

##### Chemical Crosslinking

Chemical crosslinking utilizes covalent bonds or electrostatic interactions to integrate MXenes with other materials. Deb and Jain used catechol‐based crosslinking to tune the interlayer spacing of Ti_3_C_2_ MXenes, enabling controlled drug release for targeted cancer therapy.^[^ ^62^ ^]^ Similarly, Lu et al. functionalized Ti_3_C_2_ MXenes with dopamine, enhancing photothermal stability and enabling selective interactions with cancer cells in vitro.^[^ ^69^ ^]^

PEGylation is another widely applied crosslinking strategy. Deb and Jain demonstrated that PEG‐functionalized Ti_3_C_2_ MXenes significantly improved biocompatibility and circulation time, making them suitable for imaging‐guided cancer therapy.^[^ ^62^ ^]^ PEGylated Ti_2_C MXenes provided excellent photothermal and PDT efficacy, with minimal toxicity in vivo.

##### Layer‐by‐Layer Assembly

This technique involves sequential MXenes and complementary materials deposition to form multilayered composites with precisely controlled architectures. Deb and Jain fabricated Ti_3_C_2_ and graphene oxide (GO) composites using a layer‐by‐layer assembly approach, achieving enhanced mechanical strength and superior photothermal performance for cancer therapy. A combination of Ti_2_C and molybdenum disulfide (MoS_2_) in a similar manner, creating composites with tunable optical properties for imaging‐guided multimodal therapy.^[^ ^62^ ^]^

Additional biomedical applications include antibacterial and drug‐release systems. Yang et al. developed MXene/alginate multilayered films for wound healing, demonstrating high antibacterial activity and biocompatibility.^[^ ^70^ ^]^ Ouyang et al. synthesized MXene‐GO multilayer membranes, showing exceptional ion selectivity and stability for controlled drug release applications.^[^ ^71^ ^]^

## 3 Surface Modification and Functionalization

Surface modification of MXenes plays a pivotal role in enhancing their properties for biomedical applications. These modifications typically rely on interactions such as hydrogen bonding, van der Waals forces, electrostatic adsorption, and covalent attachment. For instance, PEGylation stabilizes MXenes by forming electrostatic bonds with negatively charged surfaces, thereby improving colloidal stability and dispersibility in physiological environments. Similarly, covalent modifications, such as catechol functionalization, increase interlayer spacing and enhance drug‐loading capabilities, while NP coatings improve surface roughness and photothermal conversion efficiency. These modifications significantly alter MXene properties, including zeta potential, hydrophilicity, and interlayer spacing, which are critical for their applications in biomedical systems. Surface modifications further expand the multifunctionality of MXenes, enabling applications such as imaging, drug delivery, and multimodal therapies. Beyond Ti_3_C_2_, functionalizing other MXenes, such as Nb_2_C with PEG for improved dispersibility and Mo_2_C with polyvinyl alcohol (PVA) for pH‐responsive behavior, has expanded their applicability.

The reported MXenes often have specific surface terminations, like O, OH, or F, which make their surfaces hydrophilic. Due to the high negative zeta potential (above 40 mV), these MXenes are very stable in water‐based colloidal solutions without surfactants.^[^ ^12^, ^16^ ^]^

However, ultra‐thin MXenes that have undergone delamination are usually unstable in challenging biological environments and do not possess multifunctional properties. This is because they quickly aggregate and deposit in biological environments, similar to many other NPs employed in medical applications.^[^ ^72^, ^73^ ^]^ As a result, surface engineering is essential to give these nanosystems exceptional stability and size distribution in a physiological environment. Surface engineering can also decorate other functional materials for multiple purposes.

**Figure 7:**
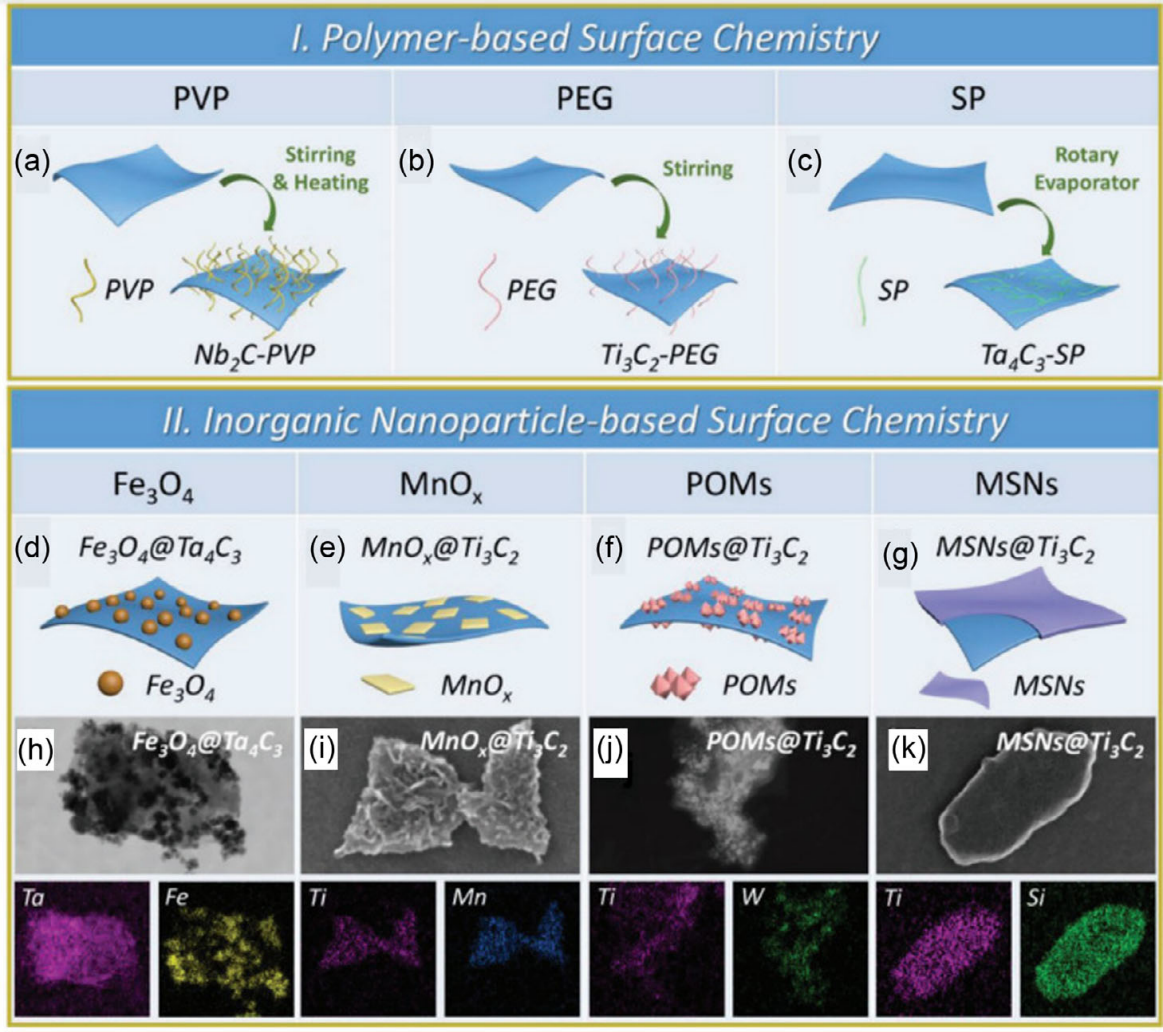
MXenes’ surface chemistry for biomedical applications. MXenes' polymer‐based surface chemistry is depicted schematically. a) M2X alteration using PVP (for instance, Nb_2_C NSs). b) PEGylation of M_3_X_2_ (e.g., Ti_3_C_2_ NSs). c) SP modification M_4_X_3_‐based MXenes. MXenes' inorganic NP‐based surface chemistry is shown schematically. d) An in situ redox reaction caused superparamagnetic iron oxide (Fe_3_O_4_) NPs to accumulate on the Ta_4_C_3_ MXene surface. It has been reproduced with the permission. e) There is a growth of in situ on the Ti_3_C_2_ surface's small MnOx NSs using a simple redox reaction. f) GdW10‐based polyoxometalates (POMs) integration over the Ti_3_C_2_ MXene's surface via an amide bond. g) Silica (SiO_2_) surface nanoporous engineered on the 2‐dimensional Ti_3_C_2_ MXene, which is a sol–gel chemistry‐based process. h) Fe_3_O_4_@Ta_4_C_3_, i) MnOx@Ti_3_C_2_, j) POMs@Ti_3_C_2_, and k) SiO_2_@Ti_3_C_2_ composite MXenes. (Panel (a–d) is reprinted with permission.^[^ ^77^ ^]^ Copyright 2018, Ivy Spring International Publisher. Reproduced with permission.^[^ ^78^ ^]^Copyright 2017, ACS, and panels are adapted with permission.^[^ ^79^ ^]^ Copyright 2018, Springer, panel (f) is reprinted with permission.^[^ ^80^ ^]^ Copyright 2018, Wiley‐VCH, panel (g) is reprinted with permission.^[^ ^31^ ^]^ Copyright 2018, Advance Science. Copyright pending; the STEM images and the corresponding element mappings in panels of (h–k) are adapted).

The following two strategies have generally been the main emphasis of MXene surface engineering: the polymer‐based surface chemistry method modifies the surface using specific macromolecules or polymers through noncovalent contact. For example, polyvinylpyrrolidone (PVP) molecules were used to adorn the Nb_2_C NSs (**Figure** **7**a).^[^ ^74^ ^]^ Soybean phospholipid (SP) was successfully utilized for the Ta_4_C_3_ NSs surface's modification through electrostatic interactions, while Ti_3_C_2_ NSs were stabilized in physiological conditions by electrostatically adsorbing PEGylation (Figure 7b,c).^[^ ^75^, ^76^ ^]^ The other is a surface chemistry based on inorganic NPs, which involves dotting MXenes with multifunctional inorganic NPs to expand the possibilities for their uses. Examples of standard methodologies for combining therapeutic agents with MRI contrast agents (CAs) for simultaneous therapy and imaging include the combination of Ta_4_C_3_ NSs with Fe_3_O_4_ NPs (Figure 7d,h) and Ti_3_C_2_ and MnOx NPs (Figure 7e,i), which gave them the ability to perform simultaneous treatment and imaging capabilities.^[^ ^77^, ^78^ ^]^ Similarly, combining 2D Ti_3_C_2_ MXene NSs with polyoxometalates (POMs) based on GdW10 offers a multimodal phototherapy platform with CT and/or MR imaging guidance for transplanted tumors. (Figure 7f,j).^[^ ^79^ ^]^ Integration of Ti_3_C_2_ MXene with the MSNs, a traditional therapeutic drug carrier, is the other typical example of a drug delivery system (DDS). However, replacing MXene with MSNs can also be a viable option for a DDS. Materials, such as appropriate mesoporous structure with controlled drug loading capacity (DOX), improved biocompatibility/dispersity/hydrophilicity (PEGylation), and the ability to attach RGD molecules to the surface for targeted drug delivery relative to conventional 2D MXenes used for targeted cancer treatment using light therapy (Figure 7g,k).^[^ ^80^ ^]^ Due to the complexity of their synthesis and integration, these two multifunctionalization techniques are still in their infancy in the graphene/GO family. The development and proliferation of various 2D MXene‐based multipurpose NP platforms for healthcare applications are anticipated to be accelerated by the rapid expansion of 2D MXene synthesis and applications.^[^ ^31^ ^]^

Future advancements in surface modification techniques will likely focus on tailoring MXene surfaces for precise interactions with biological systems. For example, incorporating bioactive ligands or antibodies could enable highly targeted therapies, while dynamic surface functionalization could allow MXenes to adapt to changing physiological environments. Achieving a balance between hydrophilicity, stability, and multifunctionality will be critical for expanding MXene applications in tissue engineering and wearable biosensors.

### 3.1 Biodegradability and Biocompatibility

MXenes exhibit remarkable biodegradability and biocompatibility, making them ideal for biomedical applications. For example, Mo_2_C‐PVA nanoflakes degrade rapidly at pH 9.4 but remain stable in mildly acidic tumor environments (pH 5.4–6.4), ensuring durability for PTT in tumor microenvironments. Comparatively, MXenes degrade faster in basic conditions than GO, which shows slower biodegradation. This pH‐responsive degradation behavior of MXenes allows for prolonged therapeutic efficacy in acidic tumor sites while ensuring rapid clearance in everyday physiological environments. High renal and fecal clearance rates and low cytotoxicity have been demonstrated for surface‐functionalized MXenes like Nb_2_C‐PVP. For example, studies show minimal toxicity in vitro for Ta_4_C_3_ and Nb_2_C MXenes, even at 400 μg mL^−1^ concentrations. In vivo tests confirm excellent biosafety, with no adverse effects observed in significant organs or hematological parameters following MXene administration. These results highlight the potential of MXenes as biocompatible and biodegradable materials for clinical translation.

**Figure 8:**
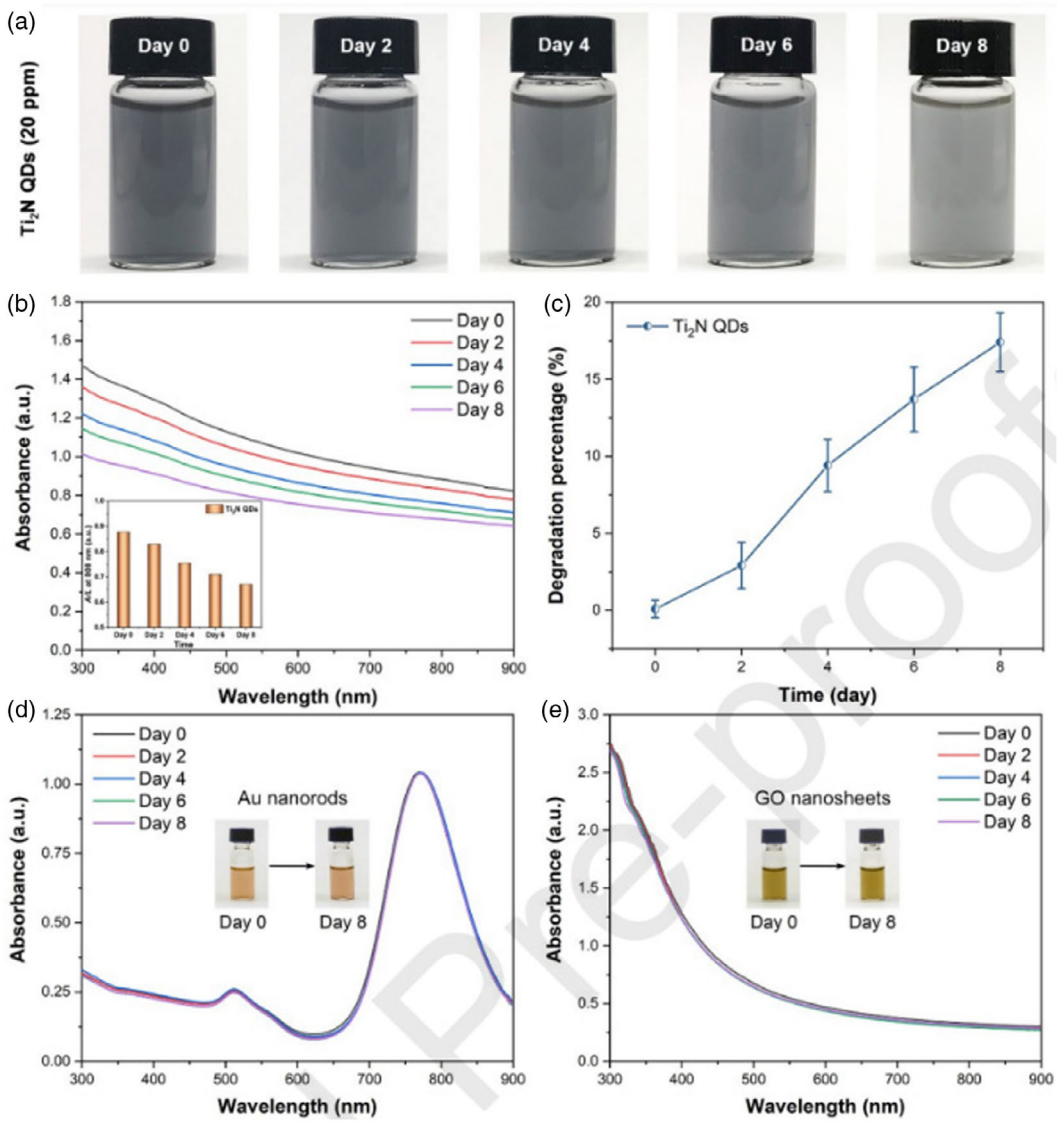
a) Images captured; b) Inset shows the intensity of absorption at 808 nm (A/L) and absorption spectra; c) The percentage of degradation in water of Ti_2_N QDs (20 ppm) over a period of 0, 2, 4, 6, and 8 days; d,e) The images (insets) and absorption spectra of degraded gold nanorods and GO NSs in H_2_O have been depicted. Reproduced with permission.^[^ ^81^ ^]^ Copyright 2020, Elsevier copyright pending.

Ti_2_N quantum dots (QDs) were evaluated for their ability to degrade in an aqueous solution, which is otherwise prone to instability due to their nitride‐based MXenes, using a concentrated dispersion of Ti_2_N QDs (20 ppm) which was constantly shaken on a horizontal shaker with air exposure at 37 °C for different durations of time (such as 0, 2, 4, 6, and 8 days). **Figure** **8** indicates that the solution gradually becomes lighter in color over time, suggesting significant degradation of Ti_2_N QDs. To examine the mechanism of biodegradation of Ti_2_N QDs, Raman analysis was conducted both before and postdegradation, which revealed that hydration and oxidation reactions during biodegradation led to the formation of hydrogen titanium oxide and titanium oxide as the end products. Compared to commonly utilized inorganic PTT agents with low biodegradability, Ti_2_N QDs have high biodegradability, making them more appropriate for PTT in medical uses. Ti_2_N QDs can maintain suitable structural stability applications like imaging and therapeutic usages during initial circulation. They could be easily eliminated posttreatment from the body, as evidenced by the appropriate excretion rate of Ti_2_N QDs from the body. This surface nanopore engineering on Ti_3_C_2_ MXene combines multiple properties conducive to expanding biomedical engineering using MXene‐based nanocomposites (**Table** **2**).^[^ ^81^ ^]^

**Table 2:** Surface modification and functionalization.

MXene/Group crafted onto the surface.	Process deployed	Chemical modifier used	Chemical modification process	Characterization	Effect of surface modification on the product	References
Ti_3_C_2_T_x_/4‐nitrophenyl	Surface‐covalent functionalization via diazonium chemistry	4‐nitrobenzene‐diazonium (4‐NBD) tetrafluoroborate salts	Ti_3_C_2_T_x_ solution is applied onto SiO_2_/Si substrates using spin coating, then treated with 4‐NBD/CH_3_CN solution in presence of N_2_	AFM, TEM	Increase in work function	[190]
Ti_3_C_2_T_x_/Polyethylene glycol PEG6	Esterification reaction	ω‐PEG6‐COOH (ω is −NH_2_, −N_3_, −CHCH)	Ti_3_C_2_T_x_ is transferred from H_2_O to DMF using solvent exchange, and then pristine Ti_3_C_2_ in DMF is mixed with PEG6‐COOH and DMAP.	Four‐point probe method, SEM, and TGA	Reduced electrical conductivity; Improved solubility in nonpolar organic solvents	[191]
Ti_3_C_2_T_x_/octyl phosphonic acid	Octyl phosphonic acid via hydrogen bonding	Octyl phosphonic acid	MXene NSs are ultrasonicated in xylene solvent, mixed with octyl phosphonic acid, and then ultrasonicated and centrifuged.	TEM, XRD, and X‐ray photoelectron Spectroscopy (XPS)	High ON/OFF rate; longer retention time; good ternary yield; better flexibility.	[192]
Ti_3_C_2_T_x_/catechols	Dehydrative condensation	Dopamine(DA) and pyrocatechol (PCAT)	A colloidal solution was prepared using the minimally intensive layer delamination method and then subjected to high‐speed centrifugation followed by catechol functionalization.	Density functional theory, UV−vis, XRD, Raman and AFM‐IR	Increased interlayer spacing; presence of nitrogen on the surface; change in particle morphology and roughness	[193]
W1.33C i‐MXene/Bovine serum albumin (BSA)	BSA as a bioactive macromolecule via H‐bonding and/or attractive van der Waals interactions	BSA	BSA solution is blended with NSs; the result is collected via centrifugation, washed with degassed ddH_2_O, and the final sediment is redispersed in degassed DI water	STEM and AFM	Biocompatible and pH‐responsive; high photothermal‐conversion efficiency; rapid degradation; selective accumulation in tumor sites.	[82]
Mo_2_C/Polyvinyl alcohol (PVA)	PVA for surface engineering via H‐bond and van der Waals interaction	Polyvinyl alcohol (PVA)	Mo_2_C‐PVA nanoflakes were combined with PVA solution, centrifuged to collect them, and then re‐dispersed in Ar‐deaerated DI water.	HRTEM, HAADF in STEM and XPS	biocompatibility; faster degradation, high photothermal‐conversion efficiency; high dispersity in physiological solutions without agglomeration	[83]
Ti_3_C_2_T_x_/Highly Reliable Superhydrophobic Protection	Selective etching with LiF/HCl aqueous solution	Fluoroalkylsilane	Ti_3_C_2_T_x_ powder is dispersed in the precursors; colloidal solution is collected after adding ethanol.	SEM, TEM, HAADF‐STEM, XPS, and BET	More stable, improved gas sensing performance	[194]

Rapid biodegradation of photothermal agents (PTAs) can lead to high in vivo biosafety and reduced long‐term toxicity. The ability of W1.33CBSA NSs to degrade depends on the pH level, with basic conditions causing rapid degradation, while they remain relatively stable under acidic conditions. This suggests that W1.33C‐BSA NSs could be more effective in delivering therapeutic results in the tumor microenvironment, which has low pH levels and a hypoxic state. The gauged pH inside 4T1 breast cancer tumors was around 6.0–6.5, indicating that W1.33C‐BSA NSs will break slowly in tumors compared to normal tissue. Degradation of W1.33C‐BSA NSs in blood is time‐dependent and increases with time.

During the initial stage, partial NSs experienced significant surface oxidation, while severe oxidation caused a slight decrease in the planar dimensions of W1.33C‐BSA NSs. Ultimately, the complete decomposition of planar dimensions occurred in the final stage. W1.33C‐BSA NSs were effectively endocytosed into tumors initially, and surface oxidation occurred in one day. After three days, prolonged incubation led to severe surface oxidation and near‐complete disintegration of W1.33C‐BSA NSs in planar dimensions, showing high biodegradation. After one hour, the PA signal intensity in muscles was only about 30% of the original. In contrast, 60% of the original PA signals were still detected in the tumor, suggesting slower degradation of W1.33C‐BSA NSs in the cancer. High levels of tungsten were found in urine and feces within 48 h after injection, indicating that the NSs were rapidly eliminated through the renal and fecal routes. Therefore, the W1.33C‐BSA NSs with high biodegradability can remain in the tumor longer and effectively degrade while exerting therapeutic effects.^[^ ^82^ ^]^

It has been revealed that Mo_2_CPVA nanoflakes display a degradation effect dependent on pH. There is rapid degradation of Mo_2_C‐PVA in basic conditions with pH values of 11.4 and 9.4. In contrast, the material has comparatively higher resistance to degradation in a low‐pH environment with pH values of 6.4, 5.4, and 3.4. This shows that the therapeutic performance of Mo_2_C‐PVA nanoflakes, which is coherent with the DLS results, would be more durable in the mildly acidic tumor microenvironment. Notably, in the cell culture medium, the decomposition rate of Mo_2_C‐PVA nanoflakes was slower than that at pH 7.4 in phosphate‐buffered saline (PBS), owing to the formation of protein corona around nanoflakes, which slowed down the degradation of Mo_2_C. Additionally, only a slight change in particle size was observed after 48 h in the nanoflakes of Mo_2_C‐PVA incorporated in a cell culture medium with 10% FBS.^[^ ^83^ ^]^

MXenes that have been evaluated for various biomedical and biological uses are confined to titanium carbide MXene (Ti_3_C_2_T_x_, Ti_2_CTx), niobium carbide MXene (Nb_2_CTx), and tantalum carbide MXene (Ta_4_C_3_Tx). Owing to the simple fabrication process of Ti_3_C_2_T_x_, it is the most extensively assessed MXene among the MXene group. Good absorption of NIR radiation by MXene makes it promising for PTT, theragnostic, and synergistic therapy for cancer treatment. Also, MXenes could be used in filter membranes due to their antimicrobial properties. MXenes were also found to be used as biosensors and in bioimaging after conversion into quantum dots.

So far, the biomedical application of 2D MXenes is still in its initial stage. The research on the cytotoxicity of MXene has been limited thus far despite the spiking interest in varied applications of MXenes. Extensive evaluation of the cytotoxicity and biocompatibility of MXene is indispensable to transforming the small‐scale lab experiments of MXenes into biomedical applications on a commercial level.^[^ ^84^ ^]^

The effects of vital characteristics of 2D nanomaterials, like dispersibility, solubility, biodegradation, and protracted toxicity, on biomedicine are not known up till now. Recently, to unveil indirectly the detrimental effects of toxicity which can impact human health, an evaluation was carried out on the ecotoxicological potential of nanomaterials based on MXene on aquatic fauna in vivo, using an embryo model of a zebrafish.

**Figure 9:**
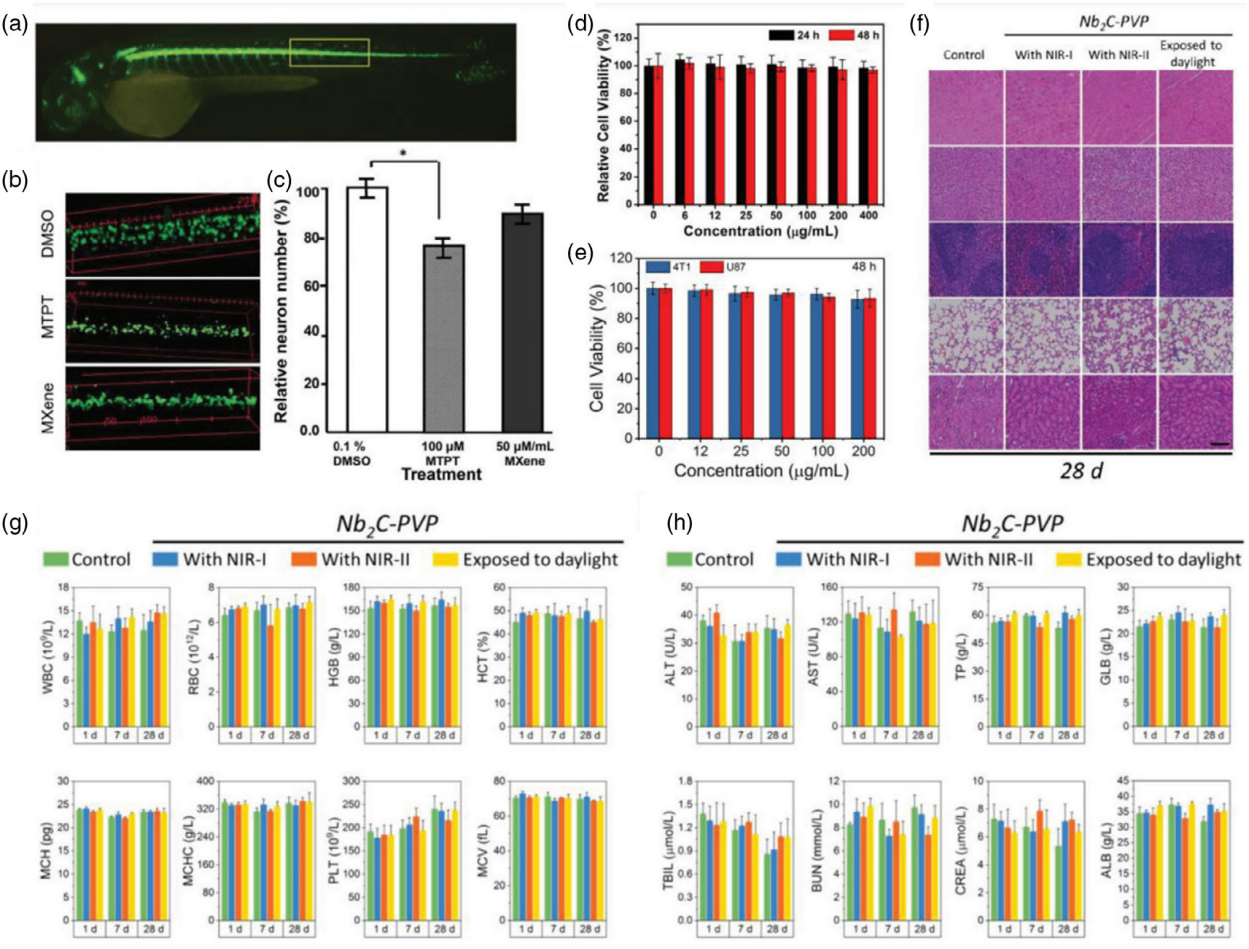
Evaluations of MXenes’ biocompatibility, ecotoxicological effects, and biosafety. a) Imaging of an unaltered embryo via confocal laser scanning microscopy. b) Spinal cord near the region of somites 14–17 where different treatments were given to sample embryos. c) The mean number of proportional neurons in untreated embryos (*P 0.05, *n* = 14). Reproduced with permission Copyright 2018, Royal Society of Chemistry. d) The comparative viability of the cell line of 4T1 following incubation with different Ta_4_C_3_‐SP NS concentrations. Reproduced with permission. Copyright 2018, Wiley‐VCH. e) The comparative viability of 4T1 as well as U87 cells following exposure to various amounts of Nb_2_C‐PVP NSs. f) Histological details (H&E stained) were taken from the key organs (spleen, liver, heart, and kidney) of mice treated with Nb_2_C‐PVP 28 days after injection under various settings (control, exposure to NIR‐I, NIR‐II, and daylight). Scale bars: 100 μm. g) Hematological parameters and h) the mice's biochemical blood indexes treated with Nb_2_C‐PVP in 1, 7, and 28 d after injection while subjected to varying treatments (control, exposure to daylight, NIR‐I, and NIR‐II). Reproduced taking the permission.^[^ ^74^ ^]^ Copyright 2017, ACS copyright pending.

The results of neurotoxicity and locomotion studies indicated that neurons present in the zebrafish embryos’ spinal cord area treated with Ti_3_C_2_T_x_ were comparable to that of the negative control group treated with dimethyl sulfoxide (DMSO), suggesting the highest nontoxic concentration of 50 μg mL^−1^. These findings, shown in **Figure** **9**a, demonstrate that Ti_3_C_2_T_x_ MXene does not have any adverse effects on muscle activity or neurons in the zebrafish embryo model, as depicted in Figure 9b,c. Furthermore, the LC50 of Ti_3_C_2_T_x_ was found to be greater than 100 μg mL^−1^, which places it in the “practically nontoxic” group according to the Fish and Wildlife Service's Acute Toxicity Rating Scale. Low cytotoxicity toward cells has been found for both MXenes and associated nanocomposites.

For example, at a concentration value equal to 400 μg mL^−1^, minimal impact was shown by the Ti_3_C_2_‐SP NSs on the 4T1 cell line viability during a 48 h coincubation period.^[^ ^85^ ^]^ In Ta_4_C_3_‐SP's vitro toxicity has been evaluated using a standard Cell Counting Kit‐8 (CCK‐8) assay. It has been observed that the 4T1 cell line survival was not significantly affected by Ta_4_C_3_‐SP, even at high concentrations values equal to 400 μg mL^−1^ (Figure 9d). Moreover, when Nb_2_C‐PVP was incubated with breast 4T1 and glioma U87 cancer cells at a concentration value of 200 μg mL^−1^, it didn't exhibit any noticeable inhibitory effect on either cell line (Figure 9e).^[^ ^74^ ^]^ Infinitesimal cytotoxicity was observed at 160 μg mL^−1^ in Mn_x_O/Ti_3_C_2_‐SP nanocomposites post 48 h coincubation, implying good biocompatibility of Mn_x_O/Ti_3_C_2_‐SP.^[^ ^78^ ^]^

Extensive investigations have been carried out in vivo to evaluate the potential biocompatibility and biosafety of surface‐modified MXene NSs in mice. For example, on 1, 7, and 28 days, healthy mice were given three doses of MXenes with surface modifications (Nb_2_C‐PVP dosage of 20 mg kg^−1^).^[^ ^75^ ^]^

The results demonstrated little variation in mouse behavior across the three treatment groups (exposed to NIR‐I, NIR‐II, and daylight) as well as the control group. Moreover, there were no fluctuations observed in mouse body weight during the three observation periods. Evaluation of major organs (liver, kidney, heart, spleen, and lung) using hematoxylin and eosin (H&E) staining after a 28‐day dietary intervention showed no indications of adverse effects or any signs of acute or chronic pathological damage on comparison of the control group with the treatment groups (Figure 9f). The blood tests, which included hematological indices and biochemical markers, were all within normal limits (Figure 9g,h). Our findings suggest that Nb_2_C‐PVP is biocompatible for in vivo tumor theragnostic treatment methods. Similar tests were carried out on Ti_3_C_2_ and Ta_4_C_3_ MXenes, which had low cytotoxicity and good in vivo biocompatibility.^[^ ^31^ ^]^

Long‐term studies on the biodegradability and cytotoxicity of MXenes are crucial for their clinical translation. While preliminary studies indicate promising biosafety profiles, understanding the degradation pathways and potential byproducts under physiological conditions will help mitigate concerns regarding long‐term toxicity. Leveraging computational tools and machine learning could accelerate the prediction of MXene biocompatibility and optimize their properties for specific biomedical applications.

## 4 Biomedical Applications of MXenes

**Figure 10:**
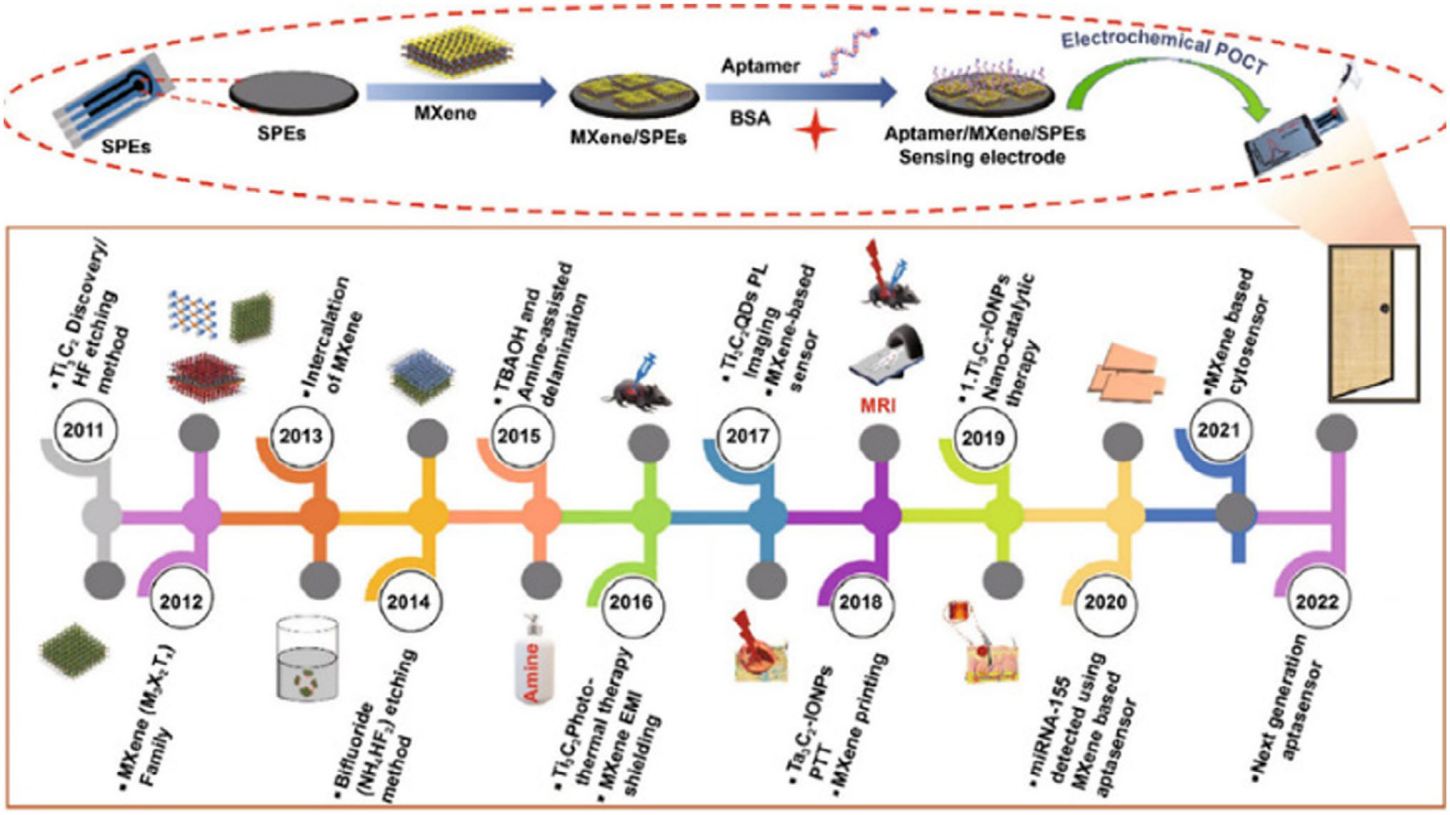
Timeline showcasing the evolution of MXene‐based materials from their discovery to advanced biomedical applications, including synthesis, functionalization, PTT, MRI imaging, and biosensing platforms.

MXene‐based materials have emerged as a promising class of 2D nanomaterials for biomedical applications due to their unique properties, including high conductivity, tunable surface chemistry, and excellent biocompatibility. Over the past decade, MXenes have transitioned from their initial discovery to multifunctional platforms for imaging, therapy, and diagnostics. **Figure** **10** highlights the progress of MXene‐based materials in biomedical applications, charting advancements from their discovery in 2011 to present‐day innovations. Starting with the HF etching method for Ti_3_C_2_ synthesis, MXenes have undergone significant development, including surface functionalization, PTT, MRI imaging, and biosensing platforms. Over the years, MXene research has expanded into next‐generation technologies such as wearable sensors, aptamer‐based diagnostics, and electrochemical point‐of‐care testing, showcasing their versatility and increasing impact in healthcare. This timeline reflects how MXene discovery has evolved from basic synthesis to multifunctional biomedical applications

### 4.1 Cancer Diagnosis and Therapy

Surface‐engineered MXenes enable multimodal therapies, such as the combination of PTT and PDT. For instance, Ti_3_C_2_ MXenes modified with ICG function as both PTAs and photosensitizers (PS), achieving simultaneous tumor ablation and ROS generation. Similarly, composites like MXene‐Fe_3_O_4_ and MXene‐MnOx integrate therapeutic capabilities with MRI contrast imaging for precise tumor localization and treatment monitoring. Mo_2_C‐PVA nanoflakes have demonstrated pH‐responsive photothermal performance, making them effective in tumor‐specific therapies with minimal off‐target effects. These surface modifications improve targeting, stability, and therapeutic efficacy, making MXene‐based composites a promising platform for cancer treatment.

Recently, research has shown that MXene exhibits both photothermal and antibacterial properties,^[^ ^86^, ^87^, ^88^ ^]^ indicating that MXene could be used as a PTA for fighting bacteria without relying on antibiotics. Furthermore, since MXene belongs to the 2D nanomaterial, it could potentially be able to load drugs similarly to GO. MXene has the potential to serve as a proficient carrier for PS in PDT and a highly effective PTA for PTT. Because of this property, MXene surpasses other nanomaterials in terms of producing a synergistic effect between PDT and PTT. However, for achieving a synergistic effect between PTT and PDT, no research has yet looked into the MXene's potential use as the PTA and the photosensitizer carrier.

**Figure 11:**
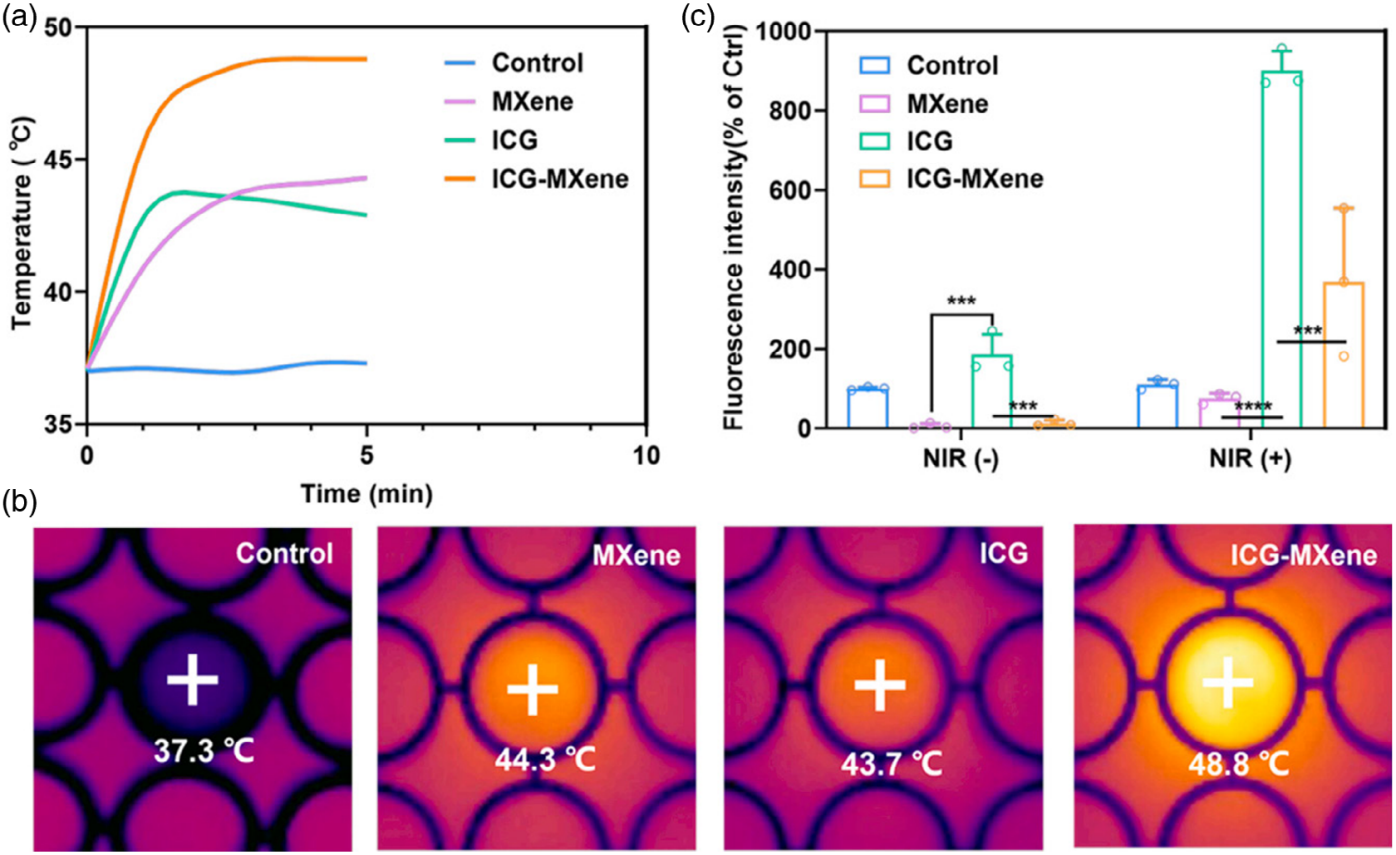
ICG‐MXene (5–20 μg mL^−1^), ICG (5 μg mL^−1^), and MXene (20 μg mL^−1^) photodynamic and photothermal effects were evaluated under NIR irradiation (808 nm, 1 W cm^−2^, 5 min). a) Temperature–time curves illustrate the temperature fluctuations during irradiation. b) Thermal images were captured at the location with the highest temperature. c) The level of ROS was assessed by quantifying the fluorescence intensity of the ROS fluorescent probe DCFH‐DA. The data represent the mean ± standard deviation (*n* = 3). Statistical significance was denoted as ***p < 0.001 and ****p < 0.0001. Reproduced with permission.^[^ ^89^ ^]^ Copyright 2022, Elsevier copyright pending.

Yu and colleagues^[^ ^89^ ^]^ utilized Ti_3_C_2_T_x_ MXene NSs with a size of 454 nm to incorporate ICG and create a hybrid effect of MXene's photothermal effect and ICG's photodynamic effect. In **Figure** **11**a, it can be observed that NIR irradiation had no impact on the temperature of water, but it caused a gradual rise in temperature to 44.3 °C of MXene suspension. The temperature of ICG rose quickly within 1–2 min under NIR radiation, but gradually decreased afterward. This behavior of ICG may be attributed to its instability, which has been previously noted in other studies.^[^ ^90^, ^91^ ^]^ In contrast, ICG‐MXene exhibited the swiftest heating (reaching a temperature of 48 °C in just 2 min) and the greatest temperature level (maintaining it at 48.8 °C for 5 min) as a result of its combination of ICG's ability to heat up quickly and MXene's ability to sustain the heat. The thermal images (shown in Figure 11b) displayed the maximum temperature of each sample. The results indicate that ICG‐MXene demonstrates a significantly enhanced photothermal effect in comparison to ICG or MXene alone. To assess the influence of NIR irradiation on the photodynamic effect of water (control), ICG‐MXene, ICG, and MXene the level of ROS was quantified using the fluorescent probe DCFH‐DA.^[^ ^92^ ^]^ When NIR was not present, the ROS level was low for all groups as shown in Figure 11c. The results of the antibacterial test indicate that at a low concentration, ICG‐MXene has minimal activity against methicillin‐resistant Staphylococcus aureus (MRSA) without NIR exposure. However, with only 5 min of NIR irradiation, the combined PTT/PDT effects lead to complete inhibition of MRSA growth.

The versatility of MXenes positions them as promising candidates for emerging biomedical technologies. For instance, MXene‐based theragnostic platforms could integrate real‐time diagnostic imaging with precise therapeutic delivery, advancing personalized medicine. Moreover, the use of MXenes in regenerative medicine, such as for bone and cartilage repair, and in developing antibacterial coatings for implants and wound dressings, represents exciting frontiers. Collaboration across materials science, biology, and clinical research will be essential to harness the full potential of MXenes in these fields.

#### 4.1.1 PDT

PDT is a medical treatment that includes the systemic administration of a photosensitizer, followed by the targeted irradiation of the affected area. This minimally invasive approach shows potential as a cancer therapy by releasing cytotoxic ROS that can directly destroy local tumor cells. To enhance the effectiveness of PDT, researchers have suggested the use of MXenes as a promising solution. MXenes can help overcome some of the limitations of traditional PS used in PDT, making them a viable option to improve medical technique efficiency. The utilization of PS like Chlorin e6 (Ce6) presents certain challenges due to their poor solubility, vulnerability to photodegradation, low efficiency of delivery, and inability to be absorbed in the most transparent regions of the skin. However, by using two‐dimensional nanomaterials (2D NMs), specifically GO, these PS can achieve better water dispersibility while maintaining superior biocompatibility. This provides a solution to the aforementioned issues.

It is essential to develop antibacterial approaches that are not reliant on antibiotics, particularly in the case of infections caused by drug‐resistant bacteria. One such approach involves synthesizing ICG‐MXene, which combines photothermal and photodynamic effects through a physical mixing process between ICG solution and MXene suspension. Although ICG‐MXene at low concentrations does not exhibit significant anti‐MRSA activity when NIR is not used, it can entirely inhibit the growth of MRSA when irradiated with NIR for a short duration of 5 min, owing to the synergistic effects of PTT/PDT. ICG‐MXene shows promise as a viable nanomaterial for phototherapy against bacteria that are resistant to antibiotics due to its low concentration and the ability to adjust irradiation parameters.

**Figure 12:**
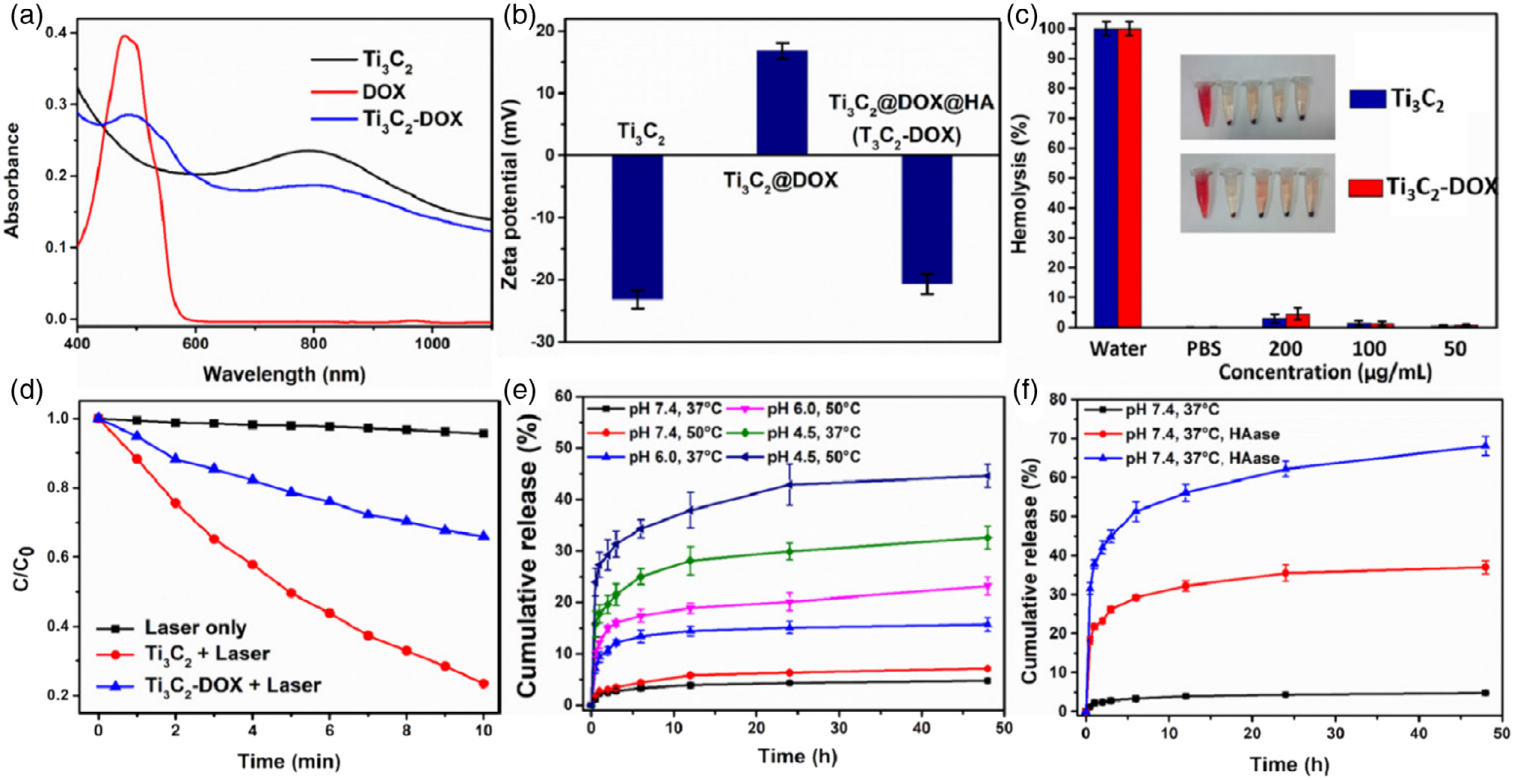
a) Ti_3_C_2_, DOX, and Ti_3_C_2_‐DOX's UV–vis absorption spectra. b) Ti_3_C_2_, Ti_3_C_2_@DOX, and Ti_3_C_2_@DOX@HA (Ti_3_C_2_‐DOX) zeta potentials. c) Ti_3_C_2_ and Ti_3_C_2_‐DOX will not both produce hemolysis (less than 5% in the tested range) in the hemolysis experiment at various doses. d) Normalized absorbance of DPBF when exposed to 808 nm light and Ti_3_C_2_ or Ti_3_C_2_Ti_3_C_2_‐DOX (0.8 W cm^−2^). e) Drug release profiles at pH 4.5, 6.0, and 7.4 with and without irradiation (808 nm, 0.8 W cm^−2^). f) Release patterns of drugs that are HAase‐responsive at pHs 4.5 and 6.0 both with and without HAase (0.5 mg mL^−1^). Reproduced with permission.^[^ ^114^ ^]^ Copyright 2017, ACS copyright pending.

The generation of singlet oxygen (1O_2_) is because of the energy transfer from photoexcited electrons in Ti_3_C_2_ to the triplet oxygen (3O_2_), which is similar to BP and graphene QD's photodynamic behavior.^[^ ^93^, ^94^ ^]^ Additionally, the localized surface plasmon resonance (LSPR) effect of Ti_3_C_2_ may contribute to oxygen generation. PDT activated by NIR laser is ideal for deep tissue penetration, and Ti_3_C_2_ NSs show promise as a new photosensitizer for the effective PDT. However, the energy exchange between oxygen and Ti_3_C_2_ may be hindered by the polymeric HA shell, resulting in a decrease in oxygen generation after HA capping. Ti_3_C_2_‐DOX NSs, prepared using sequential adsorption technique were loaded with DOX at a high loading capacity. Subsequently, HA was applied as a coating on the NSs to enhance their biocompatibility and target tumor cells that overexpress CD44^+^.^[^ ^95^, ^96^ ^]^ The effective incorporation of DOX was verified by examining the UV‐vis spectra, revealing a slight shift of the absorption peak of DOX toward longer wavelengths, specifically from 487 to 505 nm (**Figure** **12**a).^[^ ^97^ ^]^

The hydrodynamic diameters and zeta potentials of Ti_3_C_2_, Ti_3_C_2_@DOX, and Ti_3_C_2_@DOX@HA (also known as Ti_3_C_2_‐DOX) were measured. After loading DOX onto Ti_3_C_2_, its negative charge was changed to a positive charge of +13.25 mV from −23.18 mV, and then reduced again to a negative charge of −20.71 mV after being capped with HA, which was expected, as shown in Figure 12b.

In the biocompatibility tests of Ti_3_C_2_ NSs, it was observed that neither unmodified Ti_3_C_2_ NSs nor Ti_3_C_2_‐DOX caused any significant hemolysis (less than 5%) even when present in a higher conc. of 200 μg mL^−1^, as illustrated in Figure 12c. To verify the generation of singlet oxygen (1O_2_), which is the most prominent ROS, 1,3‐diphenylisobenzofuran (DPBF) was used as the detector. A decrease in DPBF absorbance demonstrates that the generated 1O2 by Ti_3_C_2_ NSs was absorbed by DPBF when it gets an exposure to NIR light, as depicted in Figure 12d.

Figure 12e,f shows Ti_3_C_2_‐DOX's stimuli‐responsive activity at various pH levels, with or without laser irradiation, or with or without HAase. In neutral environments (such as blood and interstitial fluids in healthy tissues), the HA shell successfully inhibits DOX release. The electrostatic connection in the Ti_3_C_2_‐DOX system is broken by the of Ti_3_C_2_'s laser‐induced photothermal effect (heating up to around 50 °C), which results in the release of DOX. This sensitivity to pH points to a better DDS in the acidic tumor environment. Moreover, under the influence of HAase, a significant release of up to 70% of the drug can occur within a span of 48 h at pH 4.5. These results highlight the remarkable responsiveness of Ti_3_C_2_‐DOX to pH, photothermal, and enzyme stimuli, demonstrating its capability for targeted drug release.

**Figure 13:**
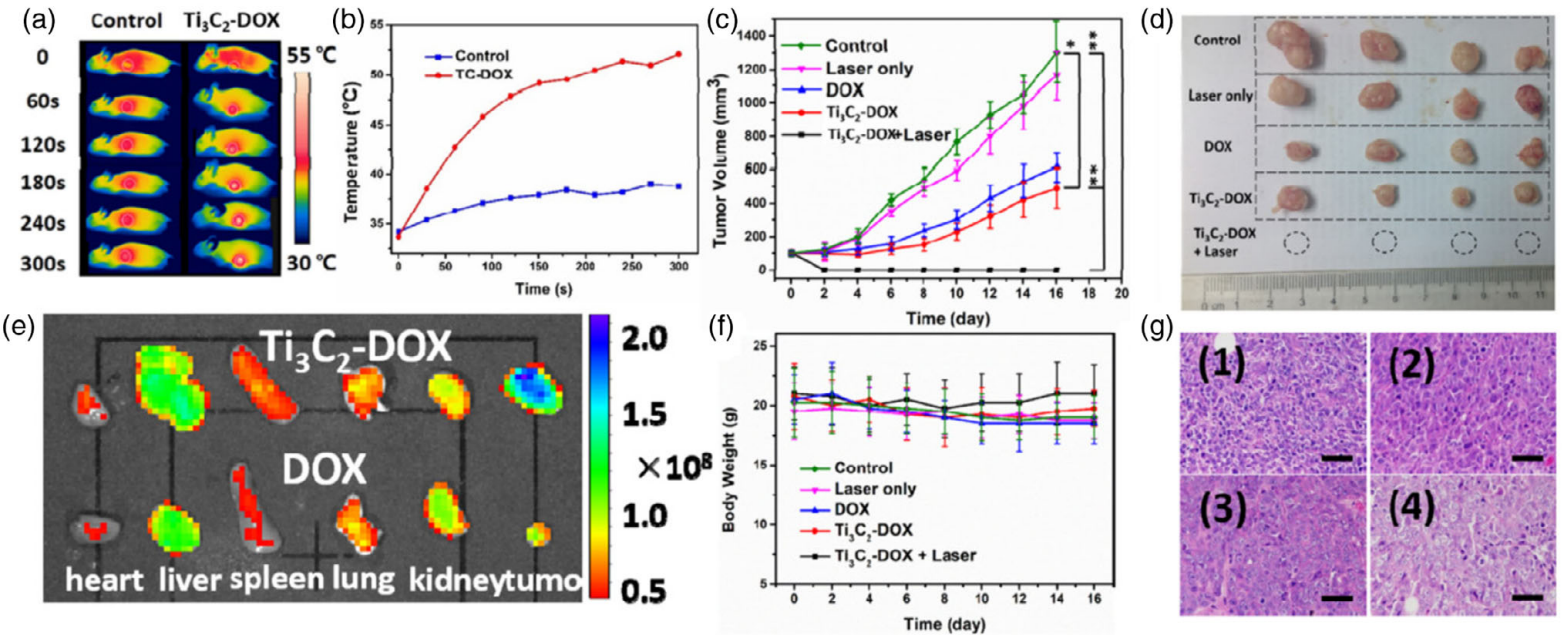
a) Infrared thermal images showing mice 6 hours post‐injection of PBS or Ti_3_C_2_‐DOX under varying irradiation durations. b) Temperature variations observed in the tumor region. c) Tumor growth trends (*n* = 4, mean ± SD, *P < 0.05, **P < 0.01) recorded throughout the treatment period. d) Digital images of tumors post‐treatment. e) Ex vivo fluorescence imaging of tumors and major organs from mice injected with DOX or Ti_3_C_2_‐DOX. f) Body weight fluctuation curves of nude mice over the treatment duration. g) Histological microscopy images of tumors following different treatments: 1) control, 2) laser irradiation alone, 3) DOX, and 4) Ti_3_C_2_‐DOX (after 16 days, scale bar: 50 μm). Reproduced with permission.^[^ ^114^ ^]^ Copyright 2017, ACS. Copyright pending.

Ti_3_C_2_‐DOX exhibits stable properties during storage at 4 °C, with no UV–vis decay or aggregation, suggesting its potential for use as a storable anticancer drug (**Figure** **13**a).

In addition, Ti_3_C_2_‐DOX's biocompatibility was considerably increased under biological circumstances after being capped with HA. Without illumination, Figure 13c compares the Ti_3_C_2_‐DOX and DOX cytotoxicity at the same dose. Figure 13b,c shows that after exposure to 808 nm laser irradiation (0.8 W cm^−2^), a considerable increment in cell death has been noticed, demonstrating cancer cells’ effective synergistic killing. Figure 13d(i,ii) shows that after laser irradiation, the tumor's temperature rose from 34.0 to 53.1 °C in about 5 min, which was enough to cause tumor ablation.^[^ ^98^, ^99^, ^100^ ^]^ treating with the Ti_3_C_2_‐DOX or free DOX in the absence of laser irradiation only resulted in partial inhibition of tumor growth. However, complete eradication of the tumor without recurrence was achieved through the combined strategy of PDT/PTT/chemotherapy utilizing Ti_3_C_2_‐DOX in the presence of laser irradiation, as depicted in Figure 13d(iii). The developed nanoplatform exhibited better biocompatibility and stimuli‐responsive drug release, along with high efficacy in tumor ablation at a low dosage when utilized with a lower power density NIR laser. The specific parameters employed were titanium carbide at a dosage of 2 mg kg^−1^, DOX loaded at 1.6 mg kg^−1^, laser wavelength of 808 nm, and a power density of 0.8 W cm^−2^. MXenes possess properties that render them suitable as PS in PDT, a noninvasive and efficacious approach to cancer treatment. PDT involves generating ROS through photochemical reactions, leading to tumor cell damage. While the majority of research on MXenes has concentrated on their application in PTT, there is growing interest in PDT, frequently in conjunction with PTT. Although the precise mechanism of ROS generation by MXenes is still not fully understood, it is thought that 1O_2_ production may happen as a result of the photo‐excited electrons transfer from Ti_3_C_2_ to triplet oxygen (3O_2_). Furthermore, ROS generation may be aided by the LSPR effect of MXenes containing transition metals, which is comparable to the capacity of gold NPs to produce ROS when exposed to visible light. The LSPR effect could potentially be improved by the MXene NSs' wide surface area.

Korupalli et al.^[^ ^101^ ^]^ synthesized a technique to improve the stability of MXene (Ti_3_C_2_) NSs under oxidative and hydration environments by modifying their surface with sodium ascorbate and dopamine. To generate multifunctional nanosystems having glucose starvation as well as photodynamic therapeutic effects, the modified NSs were then coupled with the glucose oxidase and the photosensitizer Ce6. When subjected to laser energy at 808 and 671 nm, these nanosystems showed outstanding photothermal effects with a conversion efficiency of about 45.1%) and potential for photodynamic treatment (ROS generation). The MXene‐based nanosystems were highly effective at killing cancer cells (490%) in the glucose presence and under laser irradiation, thanks to phototherapy and starvation therapy's synergistic effects.^[^ ^101^ ^]^ The MXenes usage in PDT for cancer treatment has shown promise, but more study is required to fully understand their potential. One of the main concerns is assessing their long‐term toxicity to ensure safe clinical use. In vitro and in vivo studies have been conducted to assess their cyto‐ and biocompatibility. Aside from toxicity, there are other areas where more study is required to fully explore the potential of MXenes for PDT. One of these areas is understanding the mechanisms leading to the ROS generation by these materials, which is essential for effective PDT. Although sufficient studies have been made on using MXenes for cancer treatment, certain aspects require careful consideration. These include potential nontargeted toxicity, selecting the appropriate physical‐chemical characteristics before assessing them for cancer therapy, proper characterization, and evaluating their potential biodegradability. In general, more study is required to fully understand the safety profile of these materials before they can be widely used for commercial purposes.

#### 4.1.2 Photoacoustic Imaging (PAI) (in Photothermal)

PAI is a type of biomedical imaging that is gaining popularity nowadays due to its noninvasive and nonionizing nature. This technique involves using a pulsed laser that is directed onto biological tissues, where it is absorbed by light‐absorbing domains. This absorption leads to the production of ultrasound waves, known as photoacoustic signals. An ultrasonic transducer receives these signals, which contain information about the tissue's light absorption properties, and uses them to create an image of the tissue's distribution of light absorption. Because PAI is less impacted by light scattering and has a higher spatial resolution than traditional optical imaging, it is better suited for imaging deeper tissues in living things.^[^ ^102^, ^103^ ^]^ To be an effective contrast agent in PAI, a substance must be able to convert light into heat very efficiently so that it produces a strong signal that contrasts with the PA signal produced by the surrounding tissue. NSs of MXene exhibiting the LSPR effect are considered highly desirable as contrast agents for PAI.^[^ ^104^ ^]^ Several MXene materials, such as Nb_2_CTx,^[^ ^74^ ^]^ Ti_3_C_2_T_x_^[^ ^78^, ^105^ ^]^ and Ta_4_C_3_Tx,^[^ ^106^ ^]^ have been shown to possess excellent photothermal conversion properties.

The impressive photothermal conversion efficiency and excellent biocompatibility of Ta_4_C_3_Tx‐SP distinguish it from other candidates, positioning it as suitable enough for contrast agents in PAI. The photothermal efficacy of a photothermal converter is predominantly governed by two key factors: the photothermal conversion efficiency and the extinction coefficient. The converter's ability to absorb light is indicated by its extinction coefficient, while its performance is indicated by its photothermal conversion efficiency. Ta_4_C_3_Tx NSs with outstanding NIR photothermal properties were produced by the solvent‐assisted exfoliation technique using HF etching and sonification. These NSs, which are extremely thin and two‐dimensional in shape, exhibited favorable photothermal characteristics, including a high extinction coefficient, good stability under photothermal conditions, and better photothermal conversion efficiency.^[^ ^107^ ^]^

**Figure 14:**
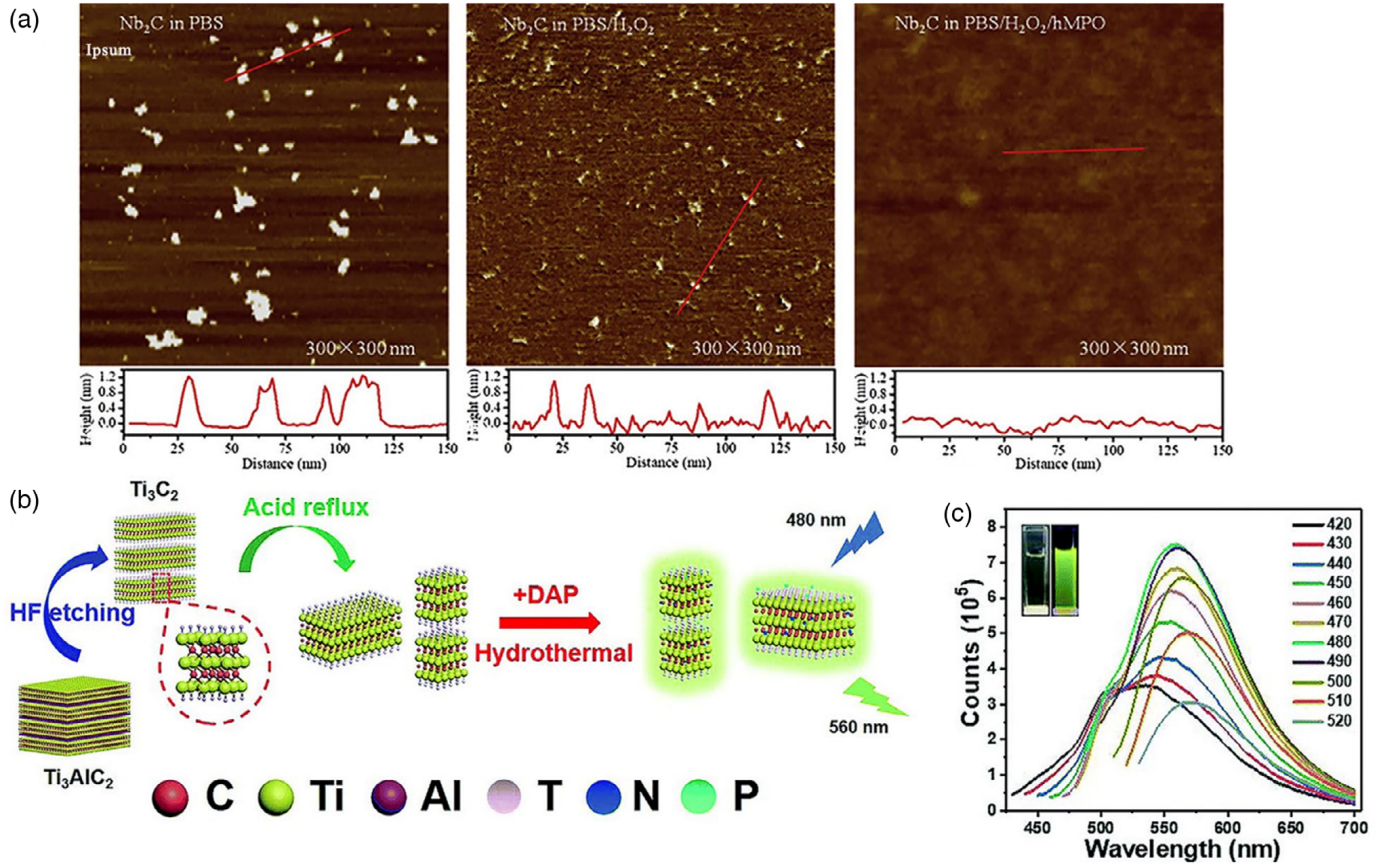
a) AFM pictures of Nb_2_CTx QDs subjected to various biodegradation treatments for 24 h, along with their corresponding height distributions. With permission from Reference, this data has been reproduced, which was published by Elsevier in 2020. b) The process for creating N, *P*‐MQD in a schematic diagram. c) The spectra that show the emission of fluorescence from *N*, *P*‐MQDs, which were created at a temperature of 120 °C, were observed at various wavelengths of excitation. Additionally, a photo taken under UV light with 365 nm wavelength was included in the analysis. Reproduced with permission.^[^ ^107^ ^]^ Copyright 2023, Journal of Nanobiotechnology. Copyright pending.

Furthermore, the Ta_4_C_3_T_x_ nanoflakes’ biocompatibility and physiological stability were greatly improved by surface modification with soybean phospholipids, and tests conducted in vivo and in vitro revealed no discernible toxicity.^[^ ^106^ ^]^ Lin et al. showed that Nb_2_CTx‐PVP (polyvinylpyrrolidone), which has potential applications in PAI, exhibited remarkably high photothermal conversion efficiency of 36.4% at NIR‐I and 45.65% at NIR‐II^[^ ^108^ ^]^ Furthermore, it demonstrated superior photothermal stability. Given their strong and broad NIR absorption characteristics, metal quantum dots (MQDs) are highly effective as imaging agents for tumor PAI, as well as fluorescence imaging Figure 14c. An MQD was created in 2019 using a fluorine‐free technique. The QDs displayed larger and more potent NIR absorption capabilities, with an extinction coefficient of up to 52.8 Lg^−1^ cm^−1^ at 808 nm and a photoconversion efficiency of up to 52.2%, as a result of the variations of AlO anions in significant quantity on their surface (**Figure** **14**). Both photothermal treatment (PTT) and PAI on tumors were simultaneously impacted by the produced MXene QDs.^[^ ^109^ ^]^ Overall, MXene‐based PAI is a good potential imaging tool for deep tissue imaging because it has a low tissue attenuation coefficient, which has the capability of overcoming the limited depth of imaging restrictions of conventional visible light imaging methods.

##### Drug Delivery Systems

MXenes are ideal for creating gene/DDSs because of their distinctive structure, which lowers drug toxicity, enables targeted drug delivery, and improves the pharmacokinetics of the molecules of the drug. The MXene materials’ nanoscale size makes it easier for them to travel through the blood system and build up where the disease is. Furthermore, the MXenes' two‐dimensional planar structure provides a sizable specific surface area that enables therapeutic molecules to adhere to the laminar structure's surface. Every year, countless people die from the common disease of cancer, which is becoming a serious threat to the health of humans. By enhancing the cellular uptake of medications and allowing the controlled release of drugs, MXene‐based materials have demonstrated promise in the fight against cancer cells.^[^ ^110^, ^111^, ^112^, ^113^ ^]^ MXenes are potentially potent anticancer agents, according to preliminary research. For instance, by sequential surface modification of hyaluronic acid (HA) and doxorubicin (DOX), Chen et al. created a multifunctional Ti_3_C_2_‐based nanoplatform (Ti_3_C_2_T_x_‐DOX). The surface of Ti_3_C_2_T_x_, the tumor‐targeted HA's negative charge, and the DOX's positive charge^[^ ^114^ ^]^ made this possible. Using tetra propylammonium hydroxide (TPAOH) intercalation, Ti_3_C_2_T_x_ was created, and after being functionalized with hydroxyl groups, its photothermal effectiveness and light harvesting ability in the NIR region were further enhanced.

The NSs’ outer layer was coated with HA to improve biocompatibility and enable active targeting of tumor cells utilizing CD44^+^ upregulation on the cancer cell membranes.^[^ ^107^ ^]^ The resulting Ti_3_C_2_T_x_‐DOX exhibited notable drug loading capacity (up to 84.2%), remarkable biocompatibility, efficient pH‐responsiveness, and responsive drug release under NIR laser stimulation, as demonstrated by in vivo and in vitro experiments. Similar to this, large specific surface area SP‐modified Ti_3_C_2_ NSs (Ti_3_C_2_T_x_‐S_P_) can also be stuffed with anticancer drugs (DOX) for effective tumor removal. High drug loading capacity, pH sensitivity, and NIR sensor drug release are all characteristics of this novel drug‐delivery nanosystem.^[^ ^115^ ^]^ However, due to their lack of confined areas for high drug loading, MXenes may encounter difficulties as drug delivery carriers. In a recent study, sol–gel chemistry was used to create surface nanopores on Ti_3_C_2_T_x_ to improve drug loading capabilities and broaden MXenes' applications in biomedicine.^[^ ^80^ ^]^ Employing tetraethylorthosilicate (TEOS) as a precursor and cetanecyltrimethylammonium chloride (CTAC) as a mesoporous guide under alkaline synthesis conditions, a thin mesoporous silica shell layer (Ti_3_C_2_T_x_@mMSNs) was applied to the surface of Ti_3_C_2_T_x_ By merging the advantages of both substances as medication transporters—a mesoporous construction that saves space, augmented water‐solubility, appropriate surface chemistry, and dispersibility—the interfacial characteristics of Ti_3_C_2_T_x_ were improved.

RGD has been covalently bounded to the Ti_3_C_2_T_x_@mMSNs modified with PEG to accomplish targeted drug delivery to the tumor site. A uniform pore size of 3.1 nm, a high pore volume of 0.96 cm^3^ g^−1^, and a sizable specific surface area of 772 m^2^ g^−1^ were all present in the prepared Ti_3_C_2_T_x_@mMSNs. These novel composite nanosystems based on MXene have been evaluated in vitro and in vivo, and the results showed that they can effectively eliminate tumors without significant recurrence when used in conjunction with standard cancer treatment modalities involving chemotherapy and localized heat therapy.

In addition, a new material called MBene has been developed for usage in post‐MXene applications. Using a chemical etching method that employs microwaves, He et al. was able to successfully synthesize the 2D NS ZBN made of zirconium boride. This material demonstrates exceptional photothermal properties in the NIR spectrum, with a high conversion efficiency equal to 76.8% in the NIRII window at a wavelength of 1060 nm.^[^ ^107^ ^]^ In addition, the material was surface‐modified using borate esterification of hyaluronic acid (HA) to achieve good dispersion, which is important for its use as a platform for multifunctional nanodelivery.

The scientists employed borate esterification to load the chemotherapy drug doxorubicin (DOX) and a nitric oxide (NO) prodrug called Gal‐NO onto the ZBN‐HA's surface, leading to significant drug loading of ZBN‐HA/DOX and ZBN‐HA/NO. Through photo‐pyrolysis, the ZBN‐HA/DOX and ZBN‐HA/NO materials underwent deconjugation of HA and aggregation of ZBN. This process facilitated photo‐controlled release of the drugs within the tumor and enabled their retention in the tumor site.^[^ ^116^ ^]^ Overall, due to their small size and flat, two‐dimensional structure, MXenes are an excellent material for using as a drug carrier.

#### 4.1.3 PTT

The strong light absorption within the NIR ranges, specifically NIR‐I (750–1000 nm) and NIR‐II (1000–1350 nm), renders MXenes highly suitable for biological applications and makes them well‐suited for applications like PTT and PAI.^[^ ^117^ ^]^ With very little impact on the viability of normal cells over a wider range measured concentrations wider range, their “light‐to‐heat” transition capacity is highly effective for the ablation of malignant cells. This may be because of the synergistic photodynamic/photothermal action and the ROS that MXenes cause to form in cancer cells. MXenes are safe for use in nanomedicine because of their photothermal conversion efficiency and high optical absorption, hydrophilicity, and composition of either essential or inert biological components.

MXenes' planar form makes it hard to accomplish high drug molecule loading, and their photothermal efficiency is still somewhat poor. With the potential for combination therapeutic effects, surface modification of MXenes can increase their photothermal stability,^[^ ^69^, ^111^ ^]^ water solubility,^[^ ^118^, ^119^, ^120^ ^]^ and drug‐loading capacity, making them suitable photothermal conversing agents and anticancer drug delivery carriers. A fluorine‐free technique can be utilized as a substitute for HF etching agents, which may represent significant toxicity hazards in MXene production procedures.

**Figure 15:**
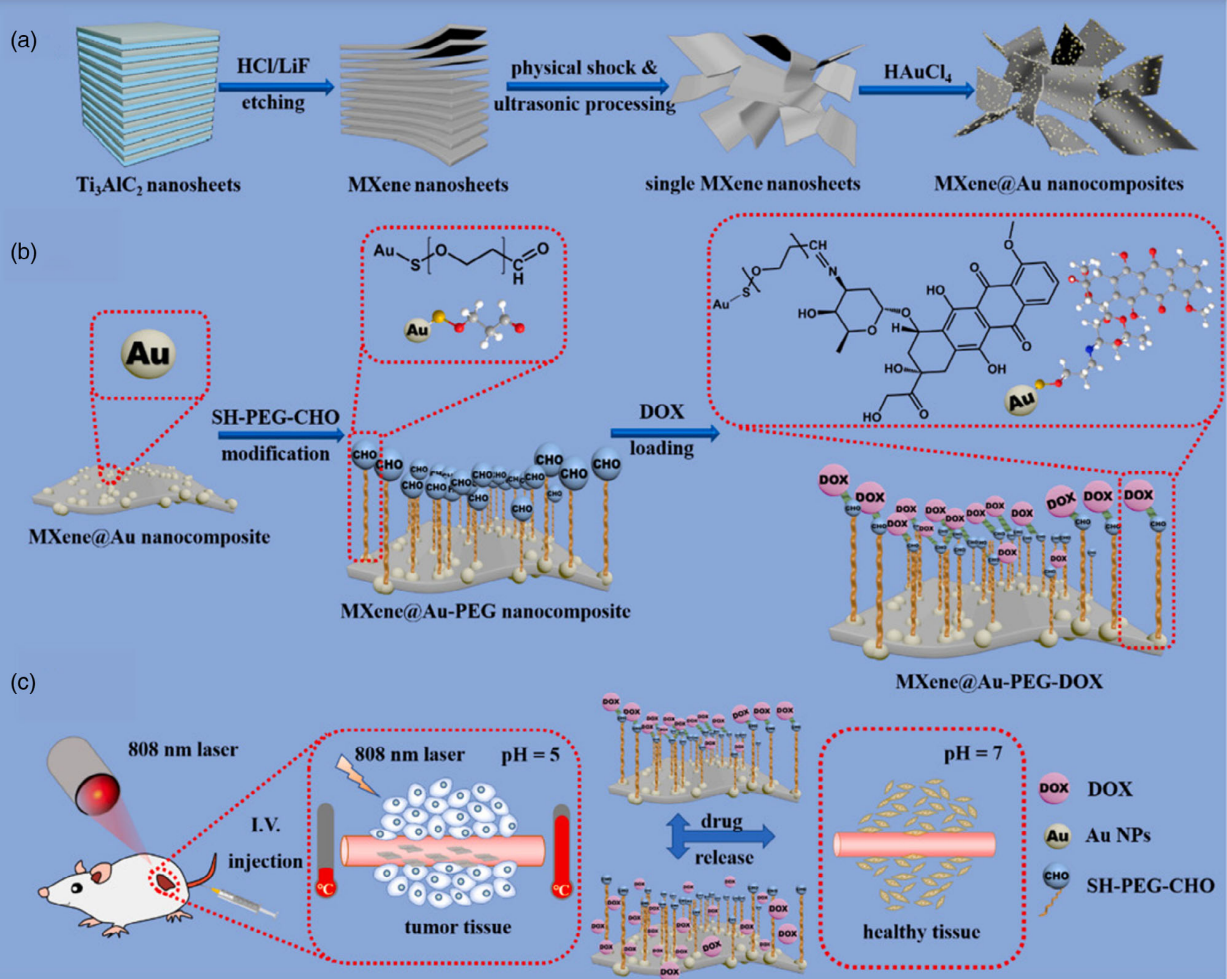
The schematic illustrates the MXene production process and its application in cancer therapy. a) The sequential steps for synthesizing MXene and MXene@Au nanosheets. b) The surface functionalization of MXene@Au with thiol polyethylene glycol aldehyde (SH‐PEG‐CHO) chains, followed by doxorubicin (DOX) loading. c) A schematic representation of the MXene‐based drug delivery system, which is pH‐responsive and activated by near‐infrared (NIR) laser irradiation for targeted tumor treatment. Reproduced with permission.^[^ ^78^ ^]^ Copyright 2017, ACS. Copyright pending.

The current advancement in MXene materials for PTT involves the fabrication of Ta_4_C_3_T_x_‐SP NSs. This enables the dual‐mode imaging of live tumors with the help of computed tomography (CT) and photoacoustic (PA) imaging techniques. Nb_2_C has an extraordinarily high photothermal conversion efficiency in NIR‐I as well as NIR‐II regions, in contrast to MQDs, which show a noticeably high photothermal conversion efficiency.^[^ ^109^ ^]^ Dai et al. used rational design to create Ti_3_C_2_ material, with the (MnO)x composition fixed to the surface of Ti_3_C_2_ via a redox reaction.^[^ ^105^ ^]^ To improve stability, the surface of the (MnO)x/Ti_3_C_2_T_x_ was further modified with soy phospholipids (SP). MnOx/Ti_3_C_2_T_x_‐SP composites were experimentally validated and demonstrated much higher photothermal stability (**Figure** **15**). Furthermore, a photothermal conversion efficiency value equal to 22.9% was achieved, which is on par with the conventional Cu_2_‐xSe carbon nanotubes (22%) and Au nanorods (21%).^[^ ^105^ ^]^ Because of the strict spatiotemporal management of local heat, PTT has less negative effects than conventional cancer treatment procedures. PTT is commonly heated beyond 50 °C for overcoming heat shock protein‐induced thermal resistance, which may cause thermal injury to vital organs near the tumor.^[^ ^121^ ^]^

A previous study found that the second biological window in the NIR (NIR‐II) range (1000–1350 nm) is more advantageous for deep tissue penetration in comparison with the first biological window (NIR‐I) (750–1000 nm).^[^ ^78^ ^]^ Cao et al. proposed a method that uses tiny fluorescent V_2_CTx QDs. It was found out that they possess better photothermal effects in the NIR‐II region for cryogenic nuclear‐targeted PTT.^[^ ^122^ ^]^ They also developed a multipurpose thermal therapy platform modified with RGD called V_2_CTx‐TAT@Ex‐RGD. Additionally, Shao et al. found that both nitride‐based MXenes and Ti2NTx QDs have particularly higher photothermal conversion efficiencies in first as well as second biological windows of the NIR spectrum (NIR‐I showing value of 48.62% at 808 nm; NIR‐II showing value of 45.51% at 1064 nm).^[^ ^81^ ^]^

**Figure 16:**
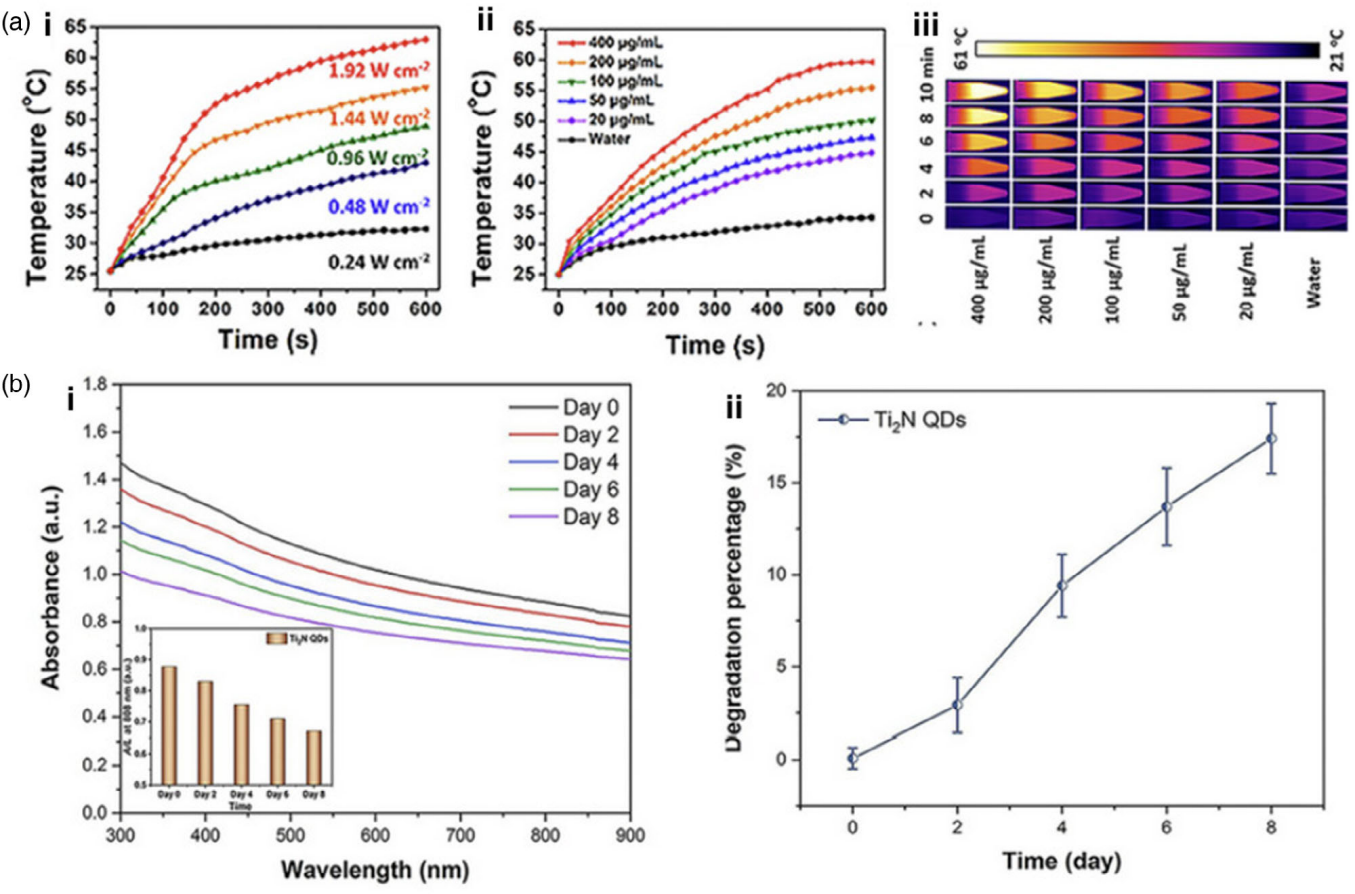
a) V_2_CTx‐TAT@Ex‐RGD (V_2_CTx‐TAT, 100 g/mL) soln. photothermal heating curves under various power densities of 1064 nanometers (NIR‐II) laser irradiation. Under 1064 nm laser illumination at various concentration values with a power density value equal to 0.96 W cm^−2^, the V_2_CTx‐TAT@Ex‐RGD solution produced photothermal heating curves (ii) and accompanying thermal pictures (iii). ACS 2019, reproduced with permission. b) Ti_2_NTx QDs' absorption spectra, including their absorption intensity at 808 nanometers (A/L) and percentage degradation following deterioration in water for a time period of 0, 2, 4, 6, and 8 days. (Panel (a) is reproduced with permission.^[^ ^122^ ^]^ Copyright 2019, ACS Nano. Panel (b) is reproduced.^[^ ^107^ ^]^ Copyright 2023, Journal of Nanobiotechnology. Copyright pending.

Ti_2_NTx QDs, besides their high photothermal therapeutic efficacy and strong biocompatibility, have great degrading properties and an efficient excretion rate in vivo, allowing for a seamless elimination from the body (**Figure** **16**b). Because of its exceptional photothermal conversion efficiency and NIR absorption properties, MXenes is showing promise as an outstanding agent for deep tissues PTT. Additionally, various surface alterations and alternative plans could enhance the photothermal properties of MXenes and facilitate the efficient eradication of cancer cells Figure 16.

PTT is a therapeutic method which had been proposed and frequently used for the treatment of cancer. The method involves the induction of localized heat in the area of the tumor cells, which results in the elimination of the tumor.

**Figure 17:**
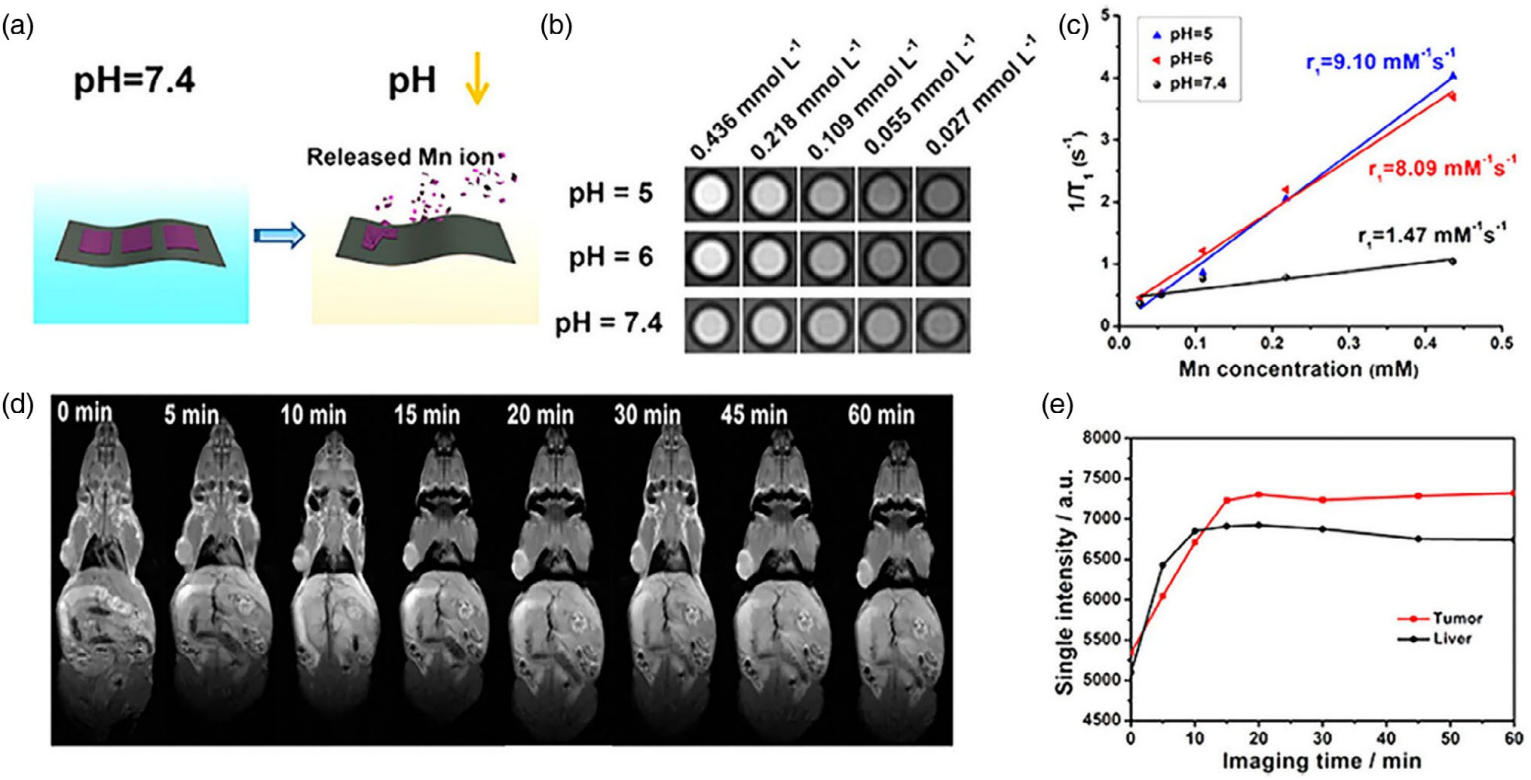
The schematic illustrates the MRI of MnOx/Ti_3_C_2_T_x_‐SP nanosheets both in vitro and in vivo, highlighting their pH‐responsive contrast enhancement. In panel (a), the diagram depicts the breakdown of the MnOx fraction under weakly acidic conditions. Panel (b) presents T1‐weighted MRI images of MnOx/Ti_3_C_2_T_x_‐SP nanosheets after being immersed in buffered solutions with different pH levels for three hours. Panel (c) displays a plot of 1/T1 versus Mn concentration for the nanosheets subjected to these varying pH conditions. In vivo experiments are shown in Panels (d) and (e), where mice were intravenously injected with the MnOx/Ti_3_C_2_T_x_‐SP nanosheets, capturing the T1‐weighted MRI images and signal intensity at different time points. The findings demonstrate that weakly acidic environments facilitate the decomposition of MnOx/Ti_3_C_2_T_x_‐SP nanosheets, leading to enhanced T1‐weighted MRI signals. Furthermore, the in vivo results confirm the nanosheets' excellent biocompatibility and their potential as effective MRI contrast agents for tumor imaging. Reproduced with permission.^[^ ^123^ ^]^ Copyright 2022, Elsevier. V copyright pending.

Liu et al. introduced a drug delivery platform using MXene@Au‐PEG, incorporating both NIR laser‐triggered and pH‐responsive drug release mechanisms for effective loading of the chemotherapeutic drug DOX (**Figure** **17**c). The Ti_3_C_2_T_x_ MXene‐based DDS was constructed by incorporating Au nanoparticles (Au‐NPs) through in situ growth (Figure 17a). For the enhancement of the electrostatic interaction between MXene and Au seeds, the zeta potential of MXene NSs was modified by introducing a positively charged polymer (PAH).^[^ ^123^ ^]^ This made MXene NSs’ surface modification easier. The reduction of HAuCl_4_ on top of the MXene by the reducing agent HCHO resulted in the formation of Au NPs in situ via seed action.

The surface of MXene@Au was changed using a PEG polymer having —SH and —CHO groups at both ends to improve drug loading capacity as well as biocompatibility and stability in physiological circulation (as shown in Figure 17b). DOX was loaded onto the MXene@Au‐PEG drug delivery platform using Schiff base bonds.^[^ ^124^ ^]^

The resulting MXene@Au‐PEG‐DOX system showed drug release modes that were responsive to both pH conditions and NIR laser stimulation. Moreover, the incorporation of Au and PEG into MXene enhanced the DDS's photothermal stability and the nanocomposites biocompatibility as well as facilitated the formation of Schiff base bonds. This improved control over drug release and enabled targeted therapeutic effects.^[^ ^16^ ^]^ Furthermore, the MXene@Au NSs were capable of generating localized photothermal conversion for tumor thermotherapy under external NIR irradiation. As a result, as shown in Figure 17c, the drug‐loaded MXene‐based DDS seems promising for efficient combined chemotherapy and PTT.^[^ ^123^ ^]^

A co‐operative anticancer nanotransporter with magnetic regulating potential and chemo‐PTT with dual stimuli‐receptive drug release was created using heterostructured titanium carbide‐cobalt (Co) nanowires. The drug packing ability of this system was notably high (225.05%), and it demonstrated strong photothermal translation efficiency when illuminated with laser (808 nm) and significant mortality for cancer cells in comparison to chemotherapy or PTT alone.

By employing ultrathin Ti_3_C_2_‐MXene NSs, a detection range from 0.00012000 ng mL^−1^ and a sensitivity of 37.9 A ng^−1^ mL cm^2^ decade^−1^ were successfully attained. These biofunctionalized MXenes and MXene‐based materials offer promising prospects for the development of highly sensitive biosensors, capable of drug and gene delivery, as well as detecting aptamers, antibodies, cells, DNA, and enzymes. By incorporating GdW10‐centered polyoxometalates, surface‐engineered and functionalized 2D Ti_3_C_2_ MXene NSs with excellent biocompatibility were produced, showing promise for treatment of hyperthermia with MR/CT imaging management for xenografts or tumor cells. During the observation phase, tumor tissue was successfully removed with no recurrence. Liu et al. created superparamagnetic 2D Ti_3_C_2_ MXenes with excellent biocompatibility, displaying high T2 relaxivity of 394.2 mM^−1^ s^−1^ and acceptable contrast‐augmented MRI of malignancies, indicating a promising future in medicinal research. In both in vitro and in vivo experiments, they showcased a significant photothermal conversion efficiency of ≈48.6% for the targeted eradication of tumors and cancer cells using photothermal treatment.

##### Mo_2_C MXenes

The study focused on ultrathin 2D molybdenum carbide (Mo_2_C) MXenes, which showed strong NIR absorption capabilities, spanning both the 1st and 2nd biological transparency windows. NIR types I and II. To improve biocompatibility and biodegradability even further, the MXenes were treated with poly(vinyl alcohol) (PVA) to form Mo_2_C‐PVA nanoflakes. The resulting nanoflakes have a broad absorption band that covers the NIR‐I as well as NIR‐II regions, as well as suitable photothermal‐translation proficiency (equal to 43.3% for NIR‐II and 24.5% for NIR‐I).^[^ ^42^ ^]^

**Figure 18:**
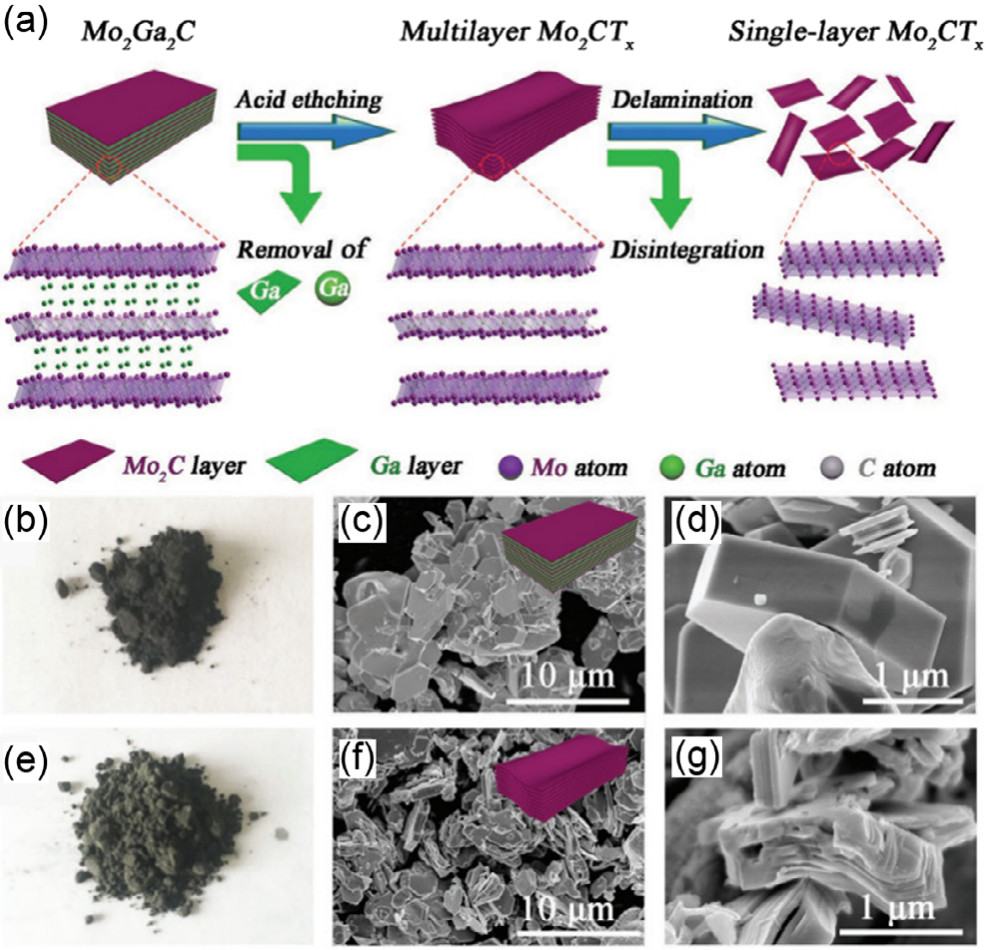
a) Mo_2_C MXene preparation and delamination are depicted schematically using a 3‐dimensional layer structure (top) and ball‐and‐stick model (bottom). b) Digital image of the bulk Mo_2_Ga_2_C ceramic and c,d) FESEM images demonstrating a typical compact layer structure. Mo_2_Ga_2_C after being treated with a hydrofluoric solution to create multilayer Mo_2_C MXenes is shown in the following images: e–g) displaying a structure of exfoliated layers. Reproduced with permission.^[^ ^83^ ^]^ Copyright 2019, Wiley Online Library. Copyright pending.

**Figure** **18**a depicts a flowchart of the technique used to generate Mo_2_C MXene. The bulk ternary layered carbide precursor powders, called as Mo_2_Ga_2_C, are immersed in an acidic etching solution containing fluoride ions. This technique eliminates the Ga atoms’ interlaced layers and dissolves the metallic connections between Ga and Mo in the bulk ceramic structure.^[^ ^12^ ^]^ The resulting layer‐structured greyish Mo_2_Ga_2_C solid bulk is sinter‐processed at 850 °C under vacuum, then immersed in a 12 m HCl solution for removing any excess Ga. (Figure 18b).^[^ ^83^ ^]^ Field‐emission scanning electron microscopy (FESEM) was employed for preliminary testing of the Mo_2_Ga_2_C powders. The images obtained showed a typical layered platelet structure with a 136.7 nm thickness (Figure 18c,d). After successful exfoliation in HF solution, the tightly bound layers grew, neatly stacked, and formed a multilayered accordion structure that produced 2D Mo_2_C flakes. (Figure 18e–g).

##### Nb_2_C MXenes

Yin et al. have made a composite NS with a 2D core/shell structure for photonic thermos‐gaseous therapy. To create the composite NSs, Nb_2_C MXene NSs were coated with a mesoporous silica layer using sol–gel chemistry. This coating improved both the biocompatibility and chemical composition, enabling surface modifications and loading of nitric oxide (NO) donors. The resulting Nb_2_C‐MSNs‐SNO composite NSs exhibited high photothermal conversion efficiency in NIR‐I (45.65%) as well as NIR‐II (36.4%) regions. Additionally, these NSs displayed variable NO release upon thermal shock.^[^ ^125^ ^]^ They also showed good PA‐imaging capabilities, allowing for real‐time monitoring of the therapeutic process.^[^ ^126^ ^]^ In vitro and in vivo tests revealed that these composite NSs have the potential to be a versatile nanomedicine for photonic‐triggered thermos‐gaseous therapy, providing a promising new cancer therapeutic approach.

##### TiCN MXenes QDs

**Figure 19:**
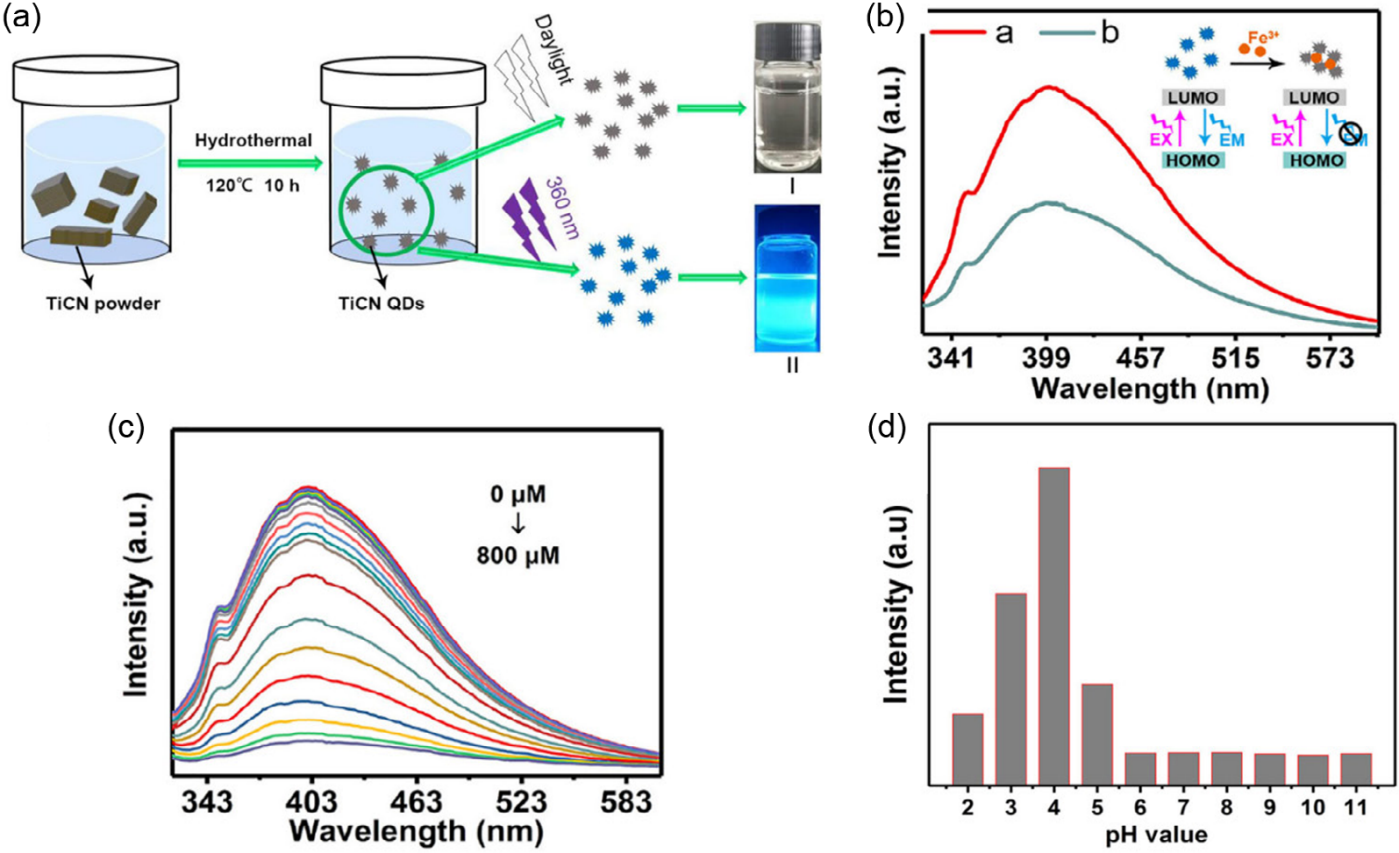
a) A schematic representation of the synthesis of TiCN quantum dots (QDs) with blue fluorescence emission using the hydrothermal method. b) Fluorescence spectra of (a) TiCN QDs and (b) TiCN QDs after the addition of Fe^3+^ at a concentration of 200 μM (The inset illustrates the mechanism of fluorescence quenching of TiCN QDs by Fe^3+^). c) Fluorescence emission spectra of TiCN QDs in the presence of varying concentrations of Fe^3+^ ions. d) Fluorescence spectra of TiCN QDs at different pH levels. Reproduced with permission.^[^ ^127^ ^]^ Copyright 2019, Elsevier. Copyright pending.

MXene QDs were synthesized utilizing a hydrothermal process with TiCN powder as the precursor, which was a straightforward and ecologically friendly procedure. (**Figure** **19**a). The MXene QDs showed exceptional fluorescence and water dispersibility, making them suitable for the selective and sensitive detection of ferric ions (Fe^3+^) and also for potential use in cancer treatment applications. This suggests investigating novel ecologically friendly and sustainable MXene production processes, and also functional modification for sensitive and selective cancer treatments.

Kong et al.^[^ ^127^ ^]^ synthesized TiCN QDs utilizing TiCN powder as a precursor in an eco‐friendly and simple hydrothermal procedure. The process involved combining TiCN powder and deionized water, followed by high‐temperature heating, cooling, and centrifugation. The generated TiCN QDs exhibited exceptional fluorescence capabilities as well as good water dispersibility. The fluorescence lifetime of these QDs was 6.33 ns, with an excitation‐wavelength‐dependent fluorescence characteristic. TiCN QD fluorescence intensity was shown to be pH‐dependent, with a maximum intensity at pH 4.0. The TiCN QDs were very sensitive to Fe^3+^ and displayed a gradual decrement in fluorescence intensity at 400 ns as the Fe^3+^ concentration increased. Adding 200 m Fe^3+^ resulted in a 42% reduction in fluorescence quenching.^[^ ^128^ ^]^ TiCN QDs could be used in biomedical applications, particularly cancer therapy, due to their superior fluorescence features and sensitive ferric ion (Fe^3+^) detection capabilities.

##### V_2_C MXenes

Cao et al. synthesized an ultrasmall 2‐dimensional vanadium carbide quantum dot (V_2_C QDs) possessing strong photothermal effects in the NIR window, as well as good fluorescence, photoacoustic, and MRI capabilities.^[^ ^122^ ^]^ A dual‐target system for cancer cell membrane and nucleus organelle was developed using small fluorescent V_2_C QDs. These QDs were modified with TAT peptides for cell nucleus targeting (V_2_C‐TAT) and have been encapsulated within endogenous exosomes. Additionally, a cell‐targeting Arg‐Gly‐Asp (RDG) peptide has been incorporated for the construction of the dual‐target system (**Table** **3**).^[^ ^129^ ^]^

**Table 3:** MXenes with diversely appealing cancer treatment and diagnosis potential.

MXenes	Strategy	Advantages	References
Mo_2_C	Cancer synergistic phototherapy utilizing the 2 multimodal imaging‐guided technique	low tissue toxicity and hematotoxicity; substantial biocompatibility^[^ ^195^ ^]^	[195]
Ti_3_C_2_	photothermal cancer therapy	In vivo/in vitro toxicity not found; There are no evident implanted components that leak into the bloodstream.^[^ ^85^ ^]^	[85]
Ti_2_C	Photothermal cancer therapy	High biocompatibility, good photothermal conversion efficiency, and high NIR‐induced potential for cancer cell death^[^ ^196^ ^]^	[196]
Nb_2_C	Photo‐/chemothermal cancer therapy	Increased photothermal hyperthermia of tumors, improved targeted chemotherapy, and low/noncytotoxicity (at 320 ng mL^−1^); a respectable photothermal conversion efficiency of 28.6%^[^ ^197^ ^]^	[197]
Nb_2_C	Photothermal cancer therapy	Higher photothermal conversion efficiency (values equal to 36.4% and 45.65%), noticeable photo thermal stability, exceptional biocompatibility, negligible phototoxicity, and side effects; specialized biodegradability with properties that respond to human myeloperoxidase enzyme^[^ ^74^ ^]^	[74]
Ti_3_C_2_	Photothermal cancer therapy	Significant photothermal conversion efficiency (52.2%); minimal in vivo/in vitro toxicity; excellent biocompatibility; obvious therapeutic use potentials imaging audiovisual^[^ ^109^ ^]^	[109]
MnOx/Ti_3_C_2_	MR/PA guided photothermal cancer therapy	Excellent biocompatibility, no toxicity that can be detected, and a good photothermal conversion efficiency (22.9%)^[^ ^78^ ^]^	[78]
MnOx/Ta_4_C_3_	Photoacoustic imaging showing the better contrast characteristics; photothermal cancer therapy	Outstanding effectiveness in halting tumor growth; little side effects; absence of notable histological lesions or abnormalities in the major organs; a decent photothermal conversion efficiency of 34.9%^[^ ^105^ ^]^	[105]
Ta_4_C_3_ with surface superparamagnetic iron oxide functionalization composites	Photothermal breast cancer therapy; MRI/CT imaging	Exceptional breast tumor photoablation efficacy, negligible toxicity, great biocompatibility, and great photothermal conversion efficiency (≈32.5%)^[^ ^77^ ^]^	[77]
Ta_4_C_3_	Photothermal cancer therapy	Great biocompatibility, no side effects, and a high rate of photothermal conversion (≈44.7%)^[^ ^198^ ^]^	[198]
Ti_3_C_2_ with GdW10 composites	Photothermal cancer therapy; MRI/CT imaging	Excellent biocompatibility, no observable cell necrosis, and a reasonable photothermal conversion efficiency of about 21.9%^[^ ^109^ ^]^	[109]
Ti_3_C_2_	Chemo‐/photothermal cancer therapy	No observable side effects, strong biocompatibility, a significant specificity/selectivity, and an acceptable photothermal conversion efficiency (58.3%).^[^ ^31^ ^]^	[31]
V_2_C	MR/PA guided photothermal cancer 2 therapy	Decent photothermal conversion efficiency (45.05%), strong tumor‐killing potency, excellent biocompatibility, and low temperature^[^ ^122^ ^]^	[122]
V_2_C	MR/PA guided photothermal cancer 2 therapy	Lower cytotoxicity, effective biodistribution, and high photothermal conversion efficiency (48%)^[^ ^199^ ^]^	[199]
Mo_2_C	Photothermal cancer therapy	Among its many benefits are its quick biodegradation, higher photothermal conversion efficiency equal to 24.5% for NIR‐I and 43.3% for NIR‐II, great biocompatibility, minimal toxicity, and effective photothermal tumor eradication.^[^ ^200^ ^]^	[200]
Nb_2_C	Photothermal cancer therapy	Photothermal conversion efficiency is satisfactory (39.09%); no acute or chronic effects are seen. (in vivo)^[^ ^125^ ^]^	[125]
Ti_3_C_2_ @Au	PA‐/CT‐guided photothermal cancer 2 therapy	Increased biocompatibility and stability; minimal long‐term toxicity;^[^ ^144^ ^]^	[144]
Ti_3_C_2_/doxorubicin hydrochloride@cellulose	Chemophotothermal cancer therapy	Substantial effectiveness for immediate tumor eradication; tailored medication release^[^ ^201^ ^]^	[201]

When subjected to a 1064 nanometers laser at a value of power density equal to 0.96 W cm2, the V_2_C‐TAT@Ex‐RGD dramatically boosted cell necrosis in vitro. In terms of in vivo cancer treatment, the extended period of blood circulation and the ability to target cancer cells of the V_2_CTAT@Ex‐effective RGD enabled tumor accumulation, which resulted in appealing antitumor therapeutic efficiency. In vivo, multimodal tumor imaging revealed that the photoacoustic (PA) intensity at the mouse's tumor site treated with V_2_C‐TAT@Ex‐RGD was found to be equal to 2.11 times higher compared to the mouse treated with V2C‐PEG‐TAT.^[^ ^122^ ^]^ When compared to the control group, the V_2_C‐PEG‐TAT‐treated mouse likewise showed a high PA signal in the tumor site. Under low‐temperature conditions, the synthesized V_2_C‐TAT@Ex‐RGD exhibited targeted PTT toward cell nuclei within the NIR‐II bio window. These findings suggest a bright prospect for future biomedical applications and novel treatments.^[^ ^74^ ^]^

### 4.2 DDSs

MXenes are ideal for creating gene/DDSs because of their distinctive structure, which lowers drug toxicity, enables targeted drug delivery, and improves the pharmacokinetics of the molecules of the drug. The MXene materials’ nanoscale size makes it easier for them to travel through the blood system and build up where the disease is. Furthermore, the MXenes' two‐dimensional planar structure provides a sizable specific surface area that enables therapeutic molecules to adhere to the laminar structure's surface. Every year, countless people die from the common disease of cancer, which is becoming a serious threat to the health of humans. By enhancing the cellular uptake of medications and allowing the controlled release of drugs, MXene‐based materials have demonstrated promise in the fight against cancer cells.^[^ ^110^, ^111^, ^112^, ^113^ ^]^ MXenes are potentially potent anticancer agents, according to preliminary research. For instance, by sequential surface modification of hyaluronic acid (HA) and doxorubicin (DOX), Chen et al. created a multifunctional Ti_3_C_2_‐based nanoplatform (Ti_3_C_2_T_x_‐DOX). The surface of Ti_3_C_2_T_x_, the tumor‐targeted HA's negative charge, and the DOX's positive charge^[^ ^114^ ^]^ made this possible. Using tetrapropylammonium hydroxide (TPAOH) intercalation, Ti_3_C_2_T_x_ was created, and after being functionalized with hydroxyl groups, its photothermal effectiveness and light harvesting ability in the NIR region were further enhanced.

The NSs’ outer layer was coated with HA to improve biocompatibility and enable active targeting of tumor cells utilizing CD44^+^ upregulation on the cancer cells membranes.^[^ ^107^ ^]^ The resulting Ti_3_C_2_T_x_‐DOX exhibited notable drug loading capacity (up to 84.2%), remarkable biocompatibility, efficient pH‐responsiveness, and responsive drug release under NIR laser stimulation, as demonstrated by in vivo and in vitro experiments." Similar to this, large specific surface area SP‐modified Ti_3_C_2_ NSs (Ti_3_C_2_T_x_‐SP) can also be stuffed with anticancer drugs (DOX) for effective tumor removal. High drug loading capacity, pH sensitivity, and NIR sensor drug release are all characteristics of this novel drug‐delivery nanosystem.^[^ ^115^ ^]^ However, due to their lack of confined areas for high drug loading, MXenes may encounter difficulties as drug delivery carriers. In a recent study, sol–gel chemistry was used to create surface nanopores on Ti_3_C_2_T_x_ to improve drug loading capabilities and broaden applications of MXenes in biomedicine.^[^ ^80^ ^]^ Employing TEOS as a precursor and CTAC as a mesoporous guide under alkaline synthesis conditions, a thin mesoporous silica shell layer (Ti_3_C_2_T_x_@mMSNs) was applied to the surface of Ti_3_C_2_T_x_ By merging the advantages of both substances as medication transporters—a mesoporous construction that saves space, augmented water‐solubility, appropriate surface chemistry, and dispersibility—the interfacial characteristics of Ti_3_C_2_T_x_ were improved.

RGD has been covalently bounded to the Ti_3_C_2_T_x_@mMSNs modified with PEG to accomplish targeted drug delivery to the tumor site. A uniform pore size of 3.1 nm, a high pore volume of 0.96 cm^3^ g^−1^, and a sizable specific surface area of 772 m^2^ g^−1^ were all present in the prepared Ti_3_C_2_T_x_@mMSNs. These novel composite nanosystems based on MXene have been evaluated in vitro and in vivo, and the results showed that they could effectively eliminate tumors without significant recurrence when used in conjunction with standard cancer treatment modalities involving chemotherapy and localized heat therapy.

In addition, a new material called MBene has been developed for usage in post‐MXene applications. Using a chemical etching method that employs microwaves, He et al. were able to successfully synthesize the 2D NS ZBN made of zirconium boride. This material demonstrates exceptional photothermal properties in the NIR spectrum, with a high conversion efficiency equal to 76.8% in the NIRII window at a wavelength of 1060 nm.^[^ ^107^ ^]^ In addition, the material was surface‐modified using borate esterification of hyaluronic acid (HA) to achieve good dispersion, which is important for its use as a platform for multifunctional nanodelivery.

The scientists employed borate esterification to load the chemotherapy drug doxorubicin (DOX) and a nitric oxide (NO) prodrug called Gal‐NO onto the ZBN‐HA's surface, leading to significant drug loading of ZBN‐HA/DOX and ZBN‐HA/NO. Through photo‐pyrolysis, the ZBN‐HA/DOX and ZBN‐HA/NO materials underwent deconjugation of HA and aggregation of ZBN. This process facilitated the photo‐controlled release of the drugs within the tumor and enabled their retention in the tumor site.^[^ ^116^ ^]^ Overall, due to their small size and flat, two‐dimensional structure, MXenes are an excellent material for using as a drug carrier.

### 4.3 MXene‐Integrated Nanocomposites

**Figure 20:**
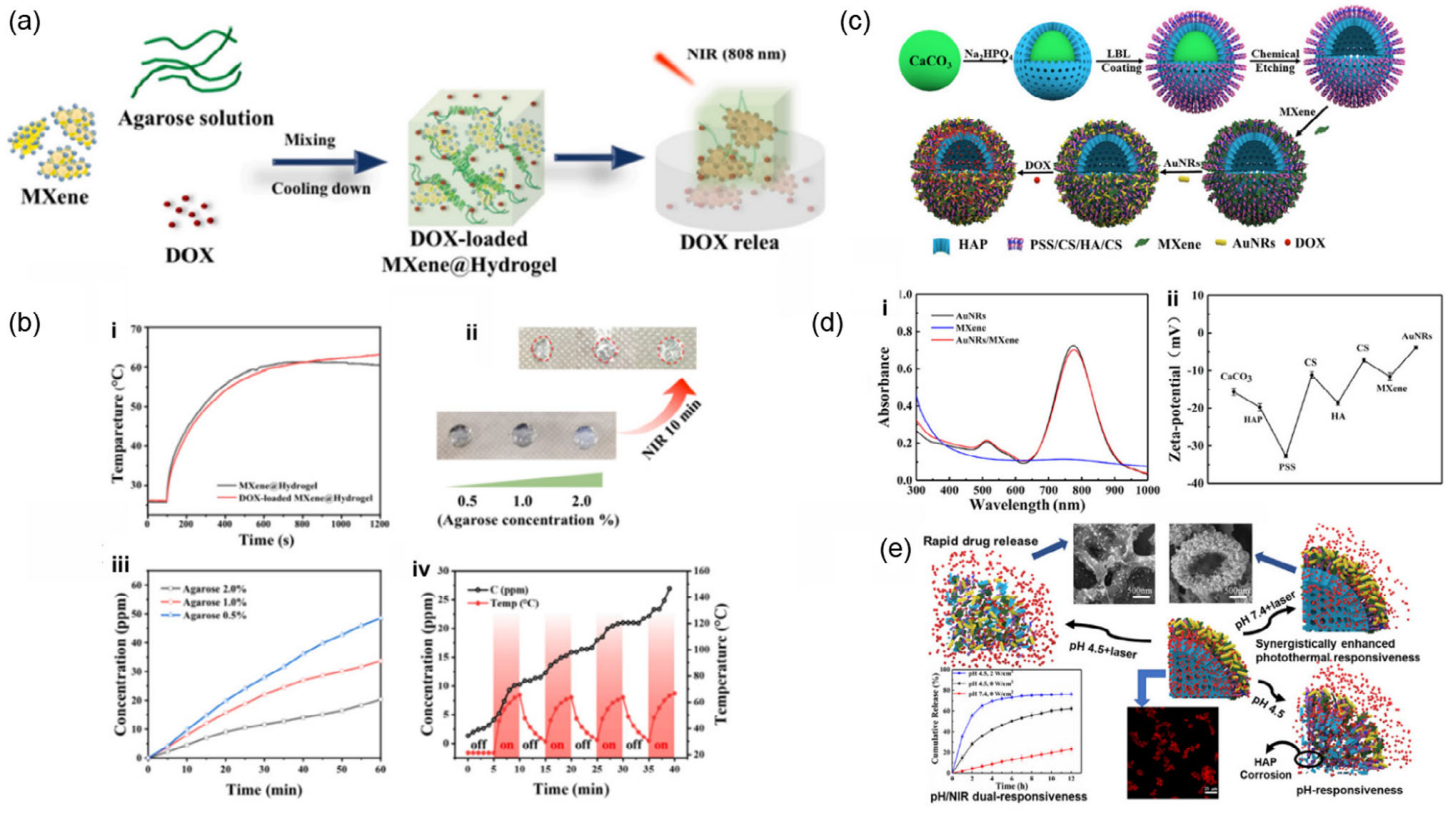
a) Creating a Mxene@Hydrogel loaded with DOX for NIR‐controlled drug release using a schematic. b) (i) MXene@Hydrogels photothermal heating curves before and after DOX injection. (ii) The MXene@Hydrogels' melting picture exposed to a 1.5 W cm^2^ laser at an 808 nm wavelength and various agarose concentrations. (iii) MXene@Hydrogels' drug release patterns that were DOX‐loaded and had various agarose concentrations. (iv) The temperature alteration and release of DOX from DOX‐loaded MXene@Hydrogel were assessed throughout four cycles of laser irradiation and subsequent cessation. c) Making CS, HAP, HA, MXene, and AuNRs microcapsules. d) AuNRs, MXene, and the combination of AuNRs and MXene UV–vis–NIR spectra; (i) zeta‐potential evolution during HAP/CS/HA/MXene/AuNRs microcapsule preparation; (ii) HAP/CS/HA and HAP/CS/HA/MXene/AuNRs microparticle XRD patterns. e) Schematic representation of the drug release process of HAP/CS/HA/MXene/AuNRs microcapsules in response to pH and NIR. Panels (a,b) are reproduced with permission.^[^ ^111^ ^]^ Copyright 2021, Elsevier. Panels (c–e) are reproduced with permission.^[^ ^136^ ^]^ Copyright 2021, Elsevier. Copyright pending.

Dong et al.^[^ ^111^ ^]^ synthesized a new DDS on the basis of MXene NSs and agarose^[^ ^130^, ^131^ ^]^ for the controlled anticancer medicine doxorubicin (DOX) release. The MXene or agarose hydrogel loaded with the drug was created by combining MXene NSs^[^ ^28^ ^]^ and DOX with agarose solution (**Figure** **20**a). The MXene@Hydrogel's photothermal heating curves before and after DOX loading were depicted in Figure 20b(i), and the MXene@Hydrogel's maximum temperature after DOX loading remained unaffected at around temperature of 60 °C, which indicates that the drug loading causes no significant impact on the MXene@Hydrogel's photothermal performance. In Figure 20b(ii), the melting behavior of MXene@Hydrogels with various agarose concentrations is depicted. The hydrogels with 0.5% and 1.0% agarose showed noticeable shrinkage, however, the hydrogel with 2.0% agarose did not display any discernible deformation. the MXene@Hydrogel loaded with DOX (20 ppm MXene, 200 ppm DOX) was exposed to NIR irradiation while employing different concentrations of agarose. The results depicted in Figure 20b(iii) indicated that higher agarose concentrations (2.0%) led to delayed and reduced drug release, whereas lower agarose concentrations (0.5%) resulted in rapid drug release. Based on these discoveries, a MXene@Hydrogel formulation consisting of 200 μg mL^−1^ DOX, 20 ppm MXene, and 1% agarose was chosen for conducting experiments related to drug release. The MXene@Hydrogel loaded with DOX demonstrated a reversible temperature increment and decrement, as depicted in Figure 20b(iv). Significantly, there was rapid release of DOX during the irradiation period (referred to as the ‘on’ stage), while minimal DOX release occurred in the absence of irradiation (the ‘off’ stage). This highlights the MXene@Hydrogel's capability to function as a NIR responsive switch. The findings show that intelligent NIR‐responsive hydrogels have huge potential for targeted cancer therapy with controlled drug delivery.

A new pH/NIR multiresponsive drug delivery platform on the basis of HAP,^[^ ^132^, ^133^ ^]^ natural polysaccharide (CS/HA), MXene, and AuNRs^[^ ^134^, ^135^ ^]^ was created by Wu et al.^[^ ^136^ ^]^ through the LbL self‐assembly technique.^[^ ^137^, ^138^ ^]^ Natural polyelectrolytes (CS/HA)^[^ ^135^, ^139^ ^]^ had been alternately coated on the HAP surface^[^ ^140^, ^141^ ^]^ for minimizing the first burst release^[^ ^142^, ^143^ ^]^ of HAP. The multilayer microcapsules were subsequently coated with NIR‐responsive MXene and AuNRs, resulting in an observable NIR reaction in the HAP‐based vehicles.^[^ ^144^, ^145^ ^]^ Finally, DOX^[^ ^146^ ^]^ was loaded into the hybrid HAP/CS/HA/MXene/AuNRs microcapsules to test their performance as smart drug carriers. To investigate the in vitro photothermal properties of hybrid microcapsules generated by NIR irradiation, photothermal conversion studies were carried out. The temperature (*T*) of the suspension containing HAP/CS/HA/MXene/AuNRs rises from 8.5 °C to a value of 38 °C within 60 min as the laser power was elevated from 1.0 W cm^−2^ to a value of 4.0 W cm^−2^, demonstrating exceptional photothermal responsiveness. At pH value of 4.5, the HAP/CS/HA/MXene/AuNRs microcapsules’ cumulative release reached 62.24% within time period of 12 h, whereas at pH 7.4, it was only 23.22%. Furthermore, the temperature increase induced by NIR irradiation enhanced the diffusion of DOX, resulting in an increased release from 23.22% (at 0 W cm^−2^ for 12 h) to 47.05% (at 2 W cm^−2^ for 12 h) at pH value of 7.4.^[^ ^147^ ^]^

**Figure 21:**
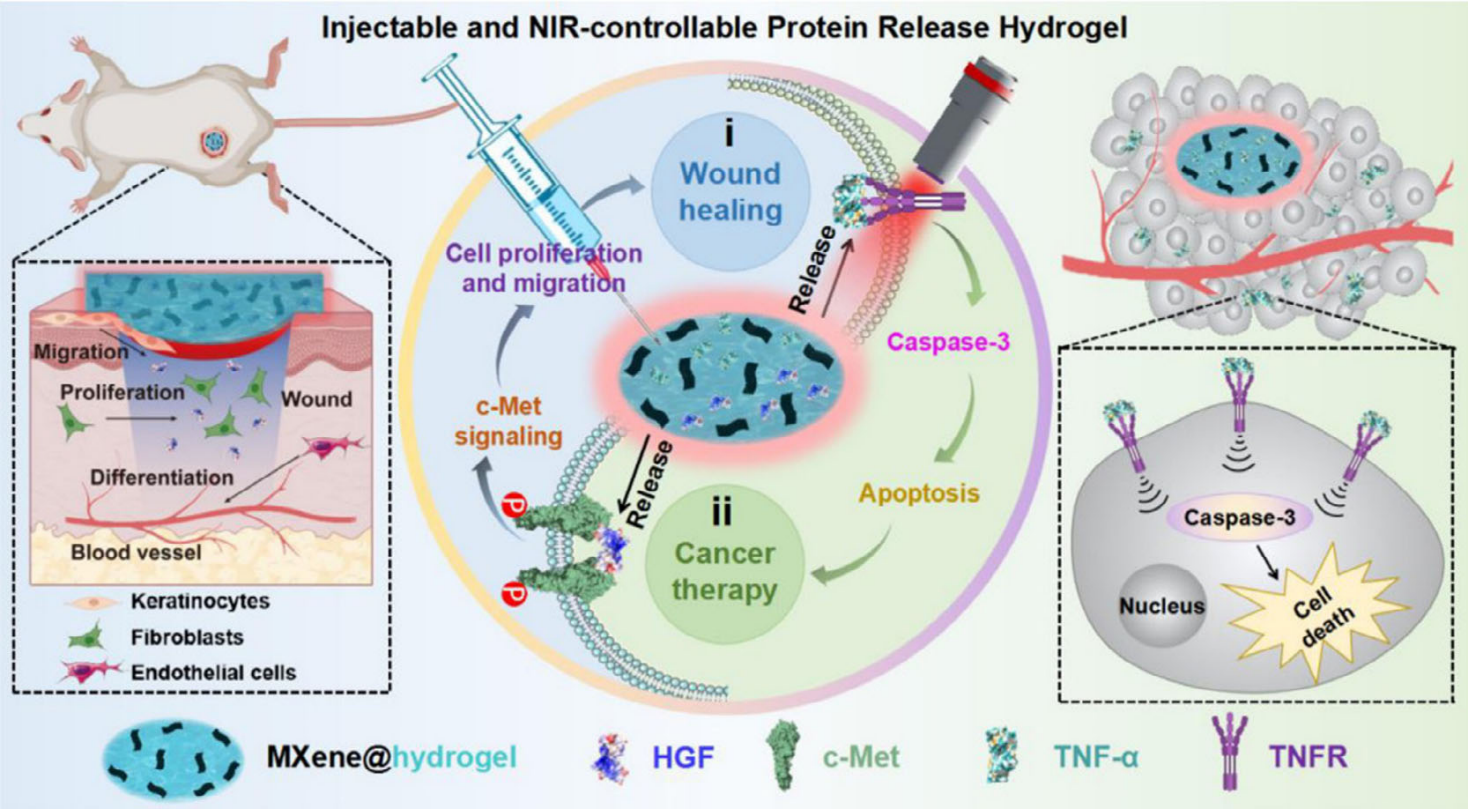
A schematic representation of how the implantable and light protein‐releasing system which can be controlled by NIR light works. This technology permits the therapeutic protein's spatiotemporally precise on‐demand discharge from an agarose hydrogel coated with Ti_3_C_2_ MXene. (i) Use a NIR light‐controllable MXene@agarose/HGF system to remotely trigger the c‐Met‐mediated signaling to control cell migration, proliferation, and further increase angiogenesis and wound repair in vivo. (ii) Exogenously eradicate the deep‐seated malignancies in a xenograft model by passively inducing receptor‐mediated programmed cell death using the MXene@agarose/TNF‐ system which can be controlled by NIR light. Reproduced with permission.^[^ ^148^ ^]^ Copyright 2021, Elsevier. Copyright pending.

Wang et al.^[^ ^148^ ^]^ developed an implantable, NIR light‐responsive composite hydrogel device that can release functional proteins on demand^[^ ^149^, ^150^ ^]^ to modulate receptor‐mediated signaling for specific cellular behaviors (**Figure** **21**). An agarose hydrogel matrix incorporates Ti_3_C_2_ MXene and therapeutic proteins in the system. The NIR light energy could be converted into heat by MXene, causing the matrix to go through a reversible phase transition and release the preloaded protein therapeutics. To precisely regulate cellular behaviors and desired cell fates, protein treatments' pattern and release profile may be altered by both internal components, such as agarose concentration, and exterior variables, such as NIR light intensity and irradiation period.

Moreover, this platform offers the potential to deliver diverse protein therapeutics into specific receptor‐mediated cell signaling pathways, thereby enhancing tumor eradication and promoting skin wound healing. In contrast to relying on unpredictable disease indicators such as pH value, enzymes, and redox environment, this system relies on precise and controlled external stimuli, like NIR light, for activation. Furthermore, alterations in patient illness indicators have no substantial impact on the system, ensuring therapy efficacy. Because of the depth at which NIR light penetrates tissue, the MXene@hydrogel/protein platform offers more benefits and universality than UV light‐responsive hydrogels and can be used to treat deep‐tissue cancers in vivo in addition to the therapeutic healing of epidermal wounds. Overall, the work shows that the MXene@hydrogel/protein system has great potential as a smart drug delivery method for precision therapeutics in both cancer therapy and regenerative medicine.

**Figure 22:**
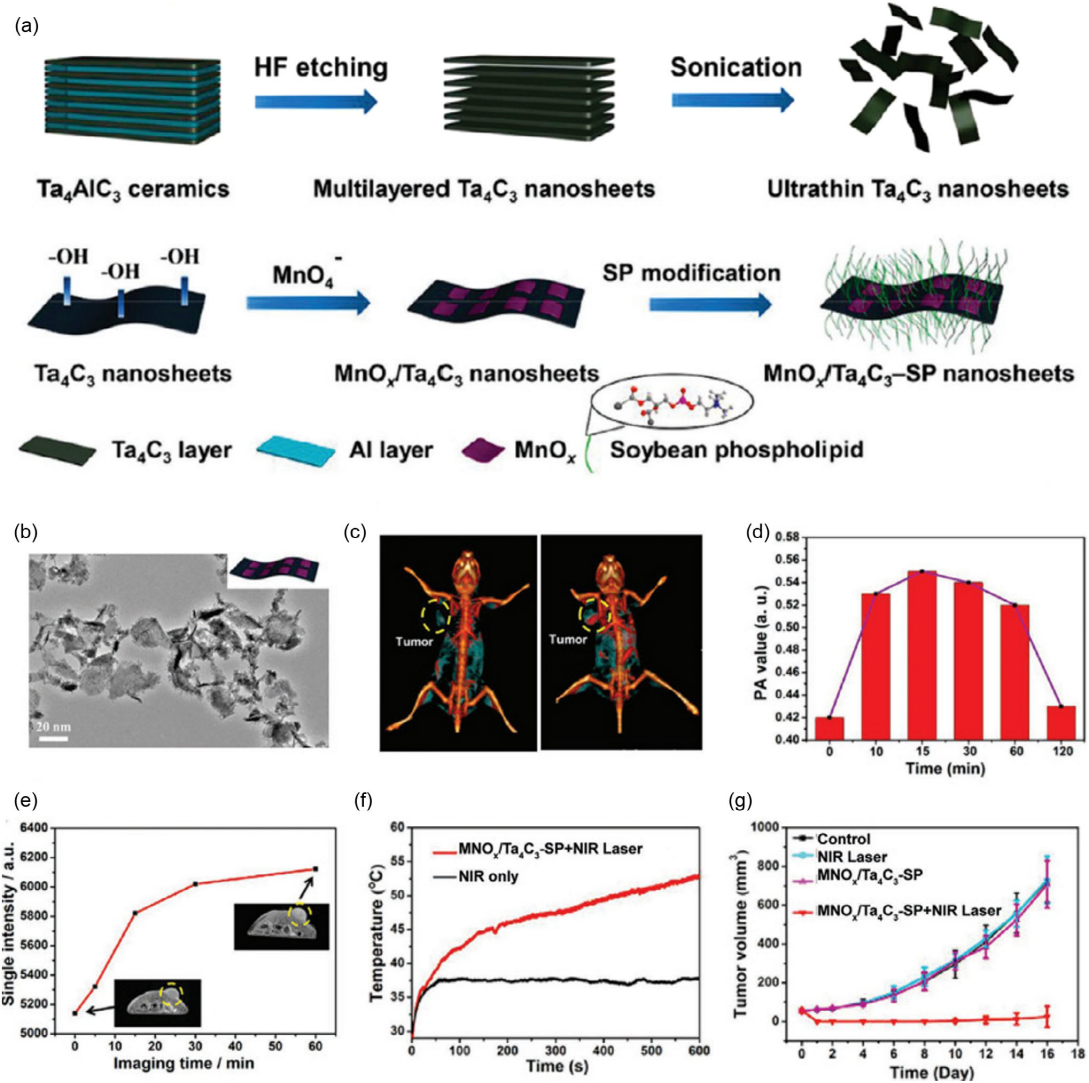
The layout, synthesis, testing, and use of MnOx/Ta_4_C_3_‐SP composite membranes for the photothermal hyperthermia of cancer in vitro and in vivo. a) Schematic representation of the MnOx/Ta_4_C_3_‐SP nanocomposite's fabrication process. b) TEM pictures of NSs made of MnOx/Ta_4_C_3_. c) Mice in vivo 3D tomography scans taken both prior to and postreceiving MnOx/Ta_4_C_3_‐SP thin sheets intravenously. After receiving the nanocomposites intravenously for extended periods of time, 4T1 tumor‐bearing animals showed changes in their d) PA signal values, and e) MRI signal intensity. f) IR thermal raised temperature curves under 808 nanometers laser irradiation at the tumor locations of nude mice implanted with 4T1 breast cancer cells. g) Variations in the relative tumor volume following various administrations. Reproduced with permission.^[^ ^105^ ^]^ Copyright 2017, ACS. Copyright pending.

Dai et al.^[^ ^105^ ^]^ studied the Ta_4_C_3_‐based composite MXene (MnOx/Ta_4_C_3_) for its potential therapeutic and diagnostic uses against cancer.^[^ ^151^, ^152^ ^]^ The ultrathin 2‐dimensional Ti_3_C_2_ MXene NSs were created using a 2‐step exfoliation of bulk Ta_4_AlC_3_ MAX‐phase ceramic^[^ ^153^ ^]^ utilizing HF etching and TPAOH intercalation. (**Figure** **22**a). The —OH groups on the surface of MXene, have reducing characteristic,^[^ ^29^, ^154^ ^]^ were discovered to react with KMnO_4_, which has strong oxidizing characteristics. As a result of the redox process, paramagnetic MnOx species formed embedded on the Ta_4_C_3_ NSs surface. For enhancing stability, composite NSs surface has been altered with the soybean phospholipid (SP), resulting in the formation of MnOx/Ta_4_C_3_‐SP. Due to tumors' enhanced permeability and retention (EPR), these precisely made composite NSs display a structure‐property correlation that is advantageous for cancer theragnostic. For MR imaging of malignancies, the pH‐responsive contrast agents are the surface‐anchored paramagnetic MnOx components. TEM images revealed a strong bonding between the sheet‐like structure of MnOx and the Ta_4_C_3_ NSs’ surface, achieved through a redox interaction between reducing —OH groups and MnO_4_‐ions (Figure 22b). The CT imaging of tumor‐bearing mice demonstrated a notable enhancement in contrast upon MnOx/Ta_4_C_3_‐SP composite NSs’ injection, as evidenced by the increase in Hounsfield Unit (HU) value (as depicted in Figure 22c). The researchers took mice's MR images with 4T1 tumors before and after administering MnOx/Ta_4_C_3_‐SP composite NSs (equal to 20 mg kg^−1^, 100 L) intravenously. They noticed a clear augmentation of the MRI signal in the tumor tissues over the next 60 min following the injection, as well as comparable increases in T1‐weighted MR images. (as shown in Figure 22e). Also, to gauge the effect of MnOx/Ta_4_C_3_–SP composite NSs in the photothermal treatment of tumors in 4T1 breast tumors, mice were split into four groups and given different treatments before being exposed to NIR laser for 10 min. After injecting MnOx/Ta_4_C_3_SP NSs, the tumors' surface temperature measurement was done utilizing an infrared thermal imaging technique. Within 10 min, the tumor's surface temperature rose to roughly 55 °C, which was sufficient to kill the tumor. The mice's weights were consistently recorded after various treatments (as shown in Figure 22f,g), and it was discovered that mice which have been treated with the MnOx/Ta_4_C_3_–SP NSs and 808 nm laser radiation had substantially inhibited tumor growth. In contrast, other three groups did not exhibit any significant inhibitory effects on tumor growth. These results indicate that these NSs can be extremely useful for further PTT guiding and monitoring.

Zhang et al.^[^ ^155^ ^]^ fabricated Ti_3_C_2_/g‐C_3_N_4_^[^ ^156^, ^157^, ^158^, ^159^ ^]^ nanocomposite films that integrate PTT and oxygen‐generating improved PDT in situ. The Al layer was eliminated from 2D Ti_3_C_2_ NSs using HF aqueous solution,^[^ ^114^, ^160^, ^161^ ^]^ and the resulting Ti_3_C_2_ sheets were then intercalated with tetrapropylammonium hydroxide (TPAOH) solution to yield ultrathin Ti_3_C_2_ NSs. From bulk g‐C_3_N_4_ that was produced using the thermal decomposition process, liquid‐phase stripping was utilized to generate few‐layer‐structured g‐C_3_N_4_ NSs. The electrostatic self‐assembly method was used to produce the heterostructured Ti_3_C_2_/g‐C_3_N_4_ NSs. The Ti_3_C_2_ incorporation to g‐C_3_N_4_ may vastly improve the material's NIR absorption, which will then enhance the light‐activated catalytic action of the resulting nanomaterials to produce higher levels of ROS. Triphenylphosphonium bromide was utilized to further alter Ti_3_C_2_/g‐C_3_N_4_ (Ti_3_C_2_/g‐C_3_N_4_‐TPP) to produce a mitochondria‐targeted and ROS‐augmented nanomedicine to work in tandem with PTT to fight cancer. Under NIR irradiation, the hybrid PDT can be realized. Ti_3_C_2_/g‐C_3_N_4_ NSs could split intrinsic water in type II PDT under low lighting to generate large amounts of oxygen. As a result, energy transfer from O_2_ to 1 O_2_ starts the production of cytotoxic 1 O_2_. To produce photoactivated holes and electrons, g‐C_3_N_4_'s valence band electrons (VB) excited the conduction band (CB) using the type I PDT. Water molecules and the excited holes interacted to form OH species. Furthermore, the Schottky junction between g‐C_3_N_4_ and Ti_3_C_2_ enables energized electrons from g‐C_3_N_4_ to transfer to Ti_3_C_2_, resulting in the production of ROS as O_2_ is reduced.^[^ ^162^, ^163^, ^164^ ^]^

Ti_3_C_2_/g‐C_3_N_4_ nanocomposites at different concentrations demonstrated a potent photothermal action under 808 nm laser irradiance. In comparison to unmodified Ti_3_C_2_ NSs, the Ti_3_C_2_/g‐C_3_N_4_ nanocomposite exhibited a significant increase in zeta potential, rising from −4.5 to −22.4 mV during in vitro experiments. Additionally, following surface modification with TPP and PEGNH2, the zeta potential rose more than 2.5 times, from −3.0 to 7.6 mV. With this modification, the nanocomposites gained excellent physiological stability in the crucial in vivo study mediums of PBS (pH value equal to 7.4 at 10 mm), FBS, and DMEM. The Ti_3_C_2_/g‐C_3_N_4_‐TPP nanocomposites demonstrated potent antitumor activity in vivo through the use of multimode PDT and PTT on mice. The Ti_3_C_2_/g‐C_3_N_4_‐TPP‐treated group did not exhibit any abnormal blood biochemical indexes, indicating that the composite did not significantly cause renal and hepatic cytotoxicity. Examination of the vital tissues via H&E staining showed no major inflammation or pathological toxicity upon intravenous administration of the NSs after two weeks. These results validated that Ti_3_C_2_/g‐C_3_N_4_‐TPP nanocomposites are biocompatible. The research conducted has expanded the potential uses of g‐C_3_N_4_ in PDT as a means of combating cancer. Additionally, it has contributed significantly to the development of PDT PS that are adaptable and capable of overcoming the limitations associated with tumor hypoxia, which has been made possible through the utilization of photocatalytic nanosystems.

**Figure 23:**
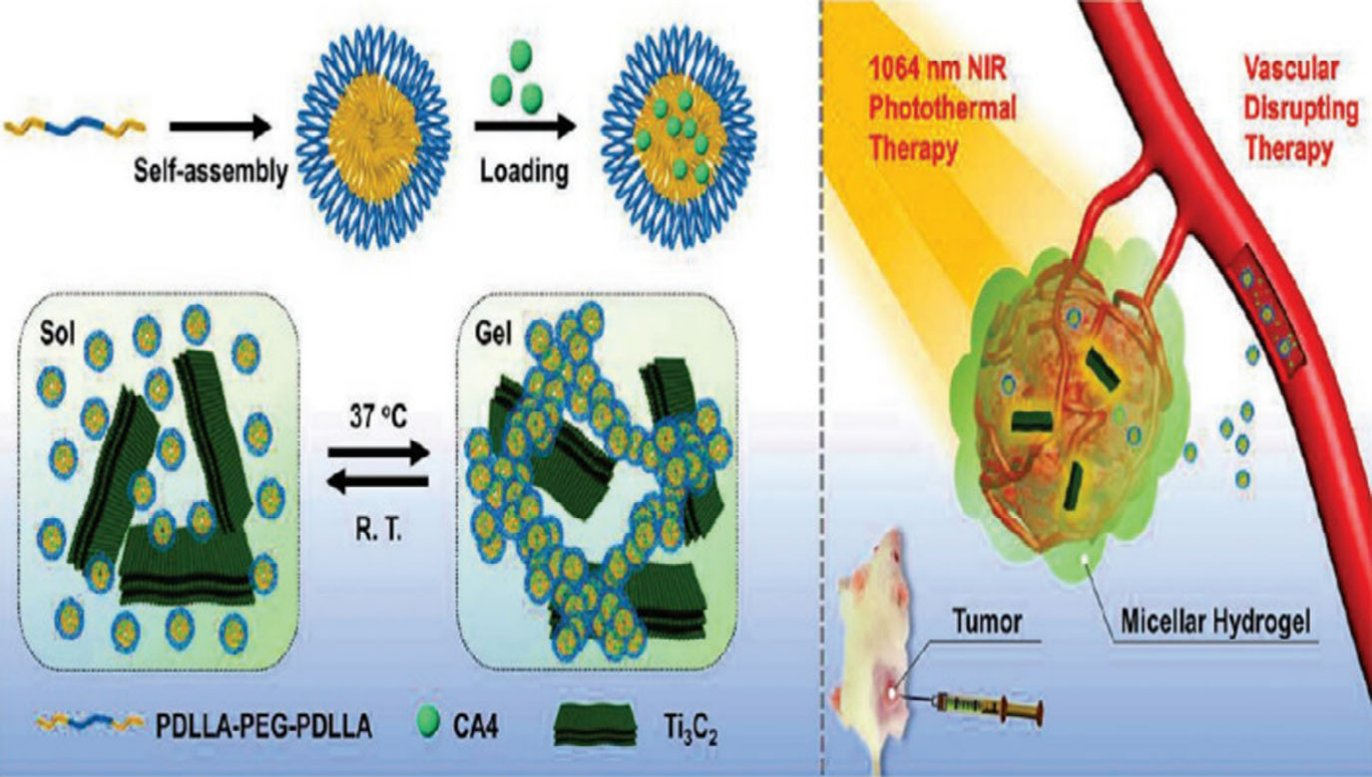
PLEL‐based implantable micellar hydrogels containing CA4 and Ti_3_C_2_ MXene are being developed for PTT and vascular disruption therapy. Reproduced with permission.^[^ ^171^ ^]^ Copyright 2020, Wiley‐VCH. Copyright pending.

Tao et al.^[^ ^165^ ^]^ created a self‐assembled polymer micelles for simultaneous loading of Combretastatin A‐4 (CA4)^[^ ^166^ ^]^ and Ti_3_C_2_ to provide combined treatment for tumor cells. (**Figure** **23**). In DI water, poly(d,l‐lactide) (PDLLA)‐PEG‐PDLLA^[^ ^97^, ^167^, ^168^ ^]^ was distributed, and the copolymer spontaneously self‐assembled to form poly(d,l‐lactide)‐poly(ethyleneglycol)‐poly(d,l‐lactide) (PLEL) micelles. When loading effectiveness was around 99.6%, the hydrophobic drug was then added to the copolymer micelle's hydrophobic core to improve solubility. PLEL micelle structures were present in the solution used to dissolve CA4, and Ti_3_C_2_ NSs were added to this solution to form the Ti_3_C_2_/CA4@copolymer micelles. At a laser power value equal to 1 W cm^−2^, the synthetic micellar hydrogel demonstrated a potential photothermal effectiveness of 41.4%. It could change at body temperature from sol to polymer. The impact of PTT was observed to be significantly affected by the extremely thin MXene structure and the use of thermal therapy. The efficacy of the hydrogel was examined in 4T1 tumor‐implanted rodents. After being subjected to 1.0 W cm^−2^ of radiation from a 1064 nm NIR laser, the tumor's temperature rose to 51.3 °C in less than five minutes. Using the Live/Dead staining method (Figure 23), Calcein AM and PI, which have green and red luminescence, respectively, were used to evaluate the ability of Ti_3_C_2_ NSs to induce hyperthermia. Nearly all of the 4T1 cells were killed when treated with Ti_3_C_2_ and subjected to 1064 nm irradiance, while the other groups did not show significant levels of cell damage. Free CA4 has been demonstrated to have a rapid clearance rate and an unfavorable dispersion in the ecological environment. However, the hydrogel encapsulation enhanced its absorption by cells.^[^ ^169^, ^170^ ^]^

Also, when the fluorescent probe Cy5 was enclosed inside the micelles, the rate of cellular internalization of the micelles gradually increased. Due to augmented retention and prolonged release of the drug, the CA4@copolymermicellar hydrogel had a stronger anticancer action than that of the unbound medication. In contrast to other groups treated, the Ti_3_C_2_/CA4@PLEL hydrogel + NIR group had higher levels of apoptosis, 5 days after the start of the therapy, based on the analysis of apoptosis/necrosis in 4T1 cancers. CD31 staining was additionally utilized to track angiogenesis. The study revealed that there were fewer vessels in the CA4‐treated groups than in the control groups (Figure 23). Ultimately, this study discovered that PTT and interruption of blood supply together had a complementary effect on cancer eradication.

**Figure 24:**
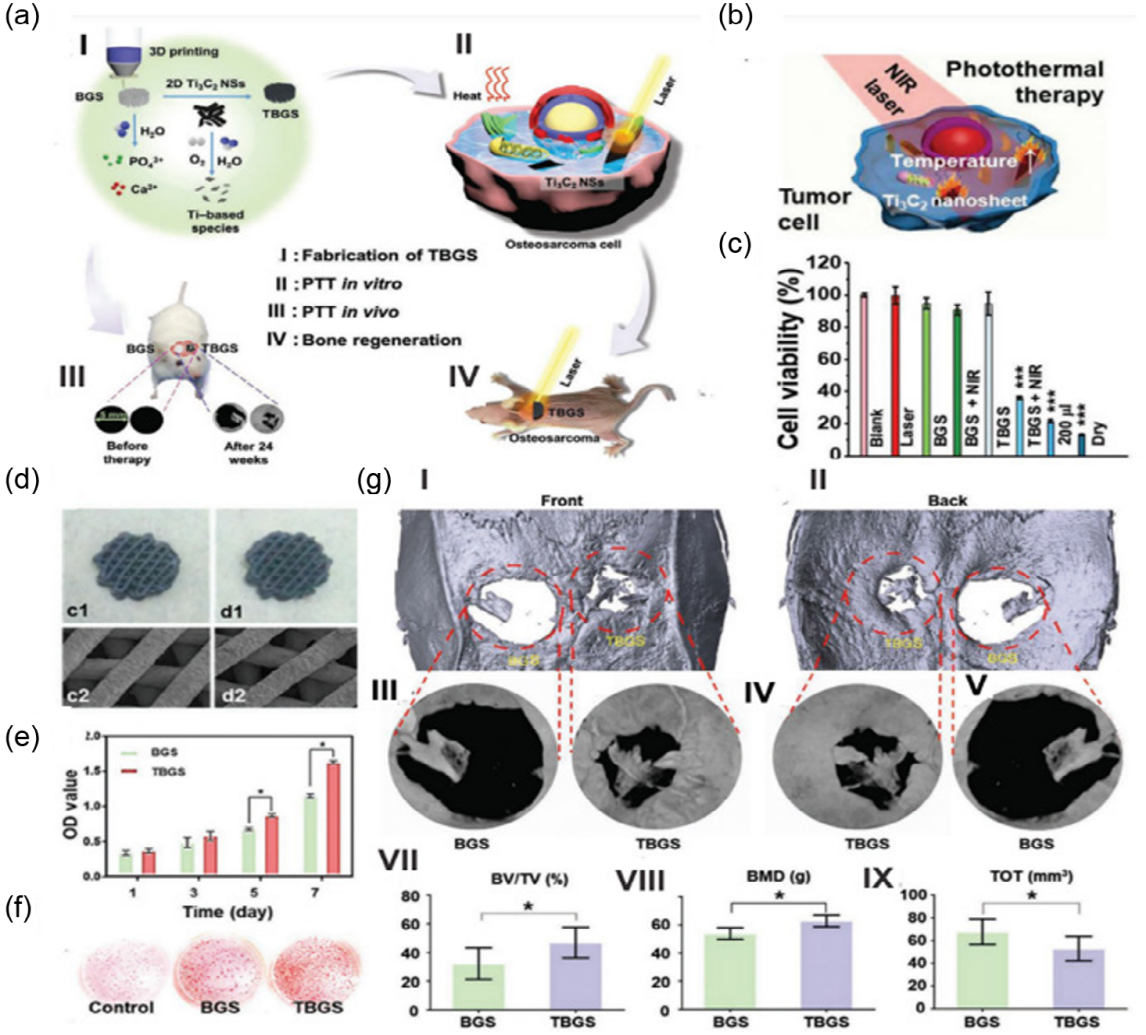
a) Ti_3_C_2_ MXene‐integrated bioactive glass scaffold (TBGS) was designed and created with the intention of destroying bone cancer and regenerating bone tissue. I) The process for making TBGS, which includes 3‐dimensional printing a pure bioactive glass scaffold (BGS), integrating Ti_3_C_2_ MXenes, and degrading those materials on the BGS. By PTT, osteosarcoma cells were eliminated using TBGS in vitro (II) as well as in vivo (III) (III). IV) Bone tissue repair following TBGS and BGS implantation. b) Diagram showing the removal of malignant cells by Ti_3_C_2_ NSs present on TBGS. c) The relative cell viability of Saos‐2 bone tumor cells following therapy under various circumstances. The “200 L” and “Dry” groups refer to the TBGS + NIR group in a dry environment (no DMEM) and 200 L Dulbecco's modified Eagle's medium, respectively. 400 L of DMEM were used for the other groups' experiments. d) Pure BGS (c1, c2) and TBGS pictures captured in photos and SEM scans (d1, d2). Scale bars for images are 3 mm and 500 m, respectively. e) The growth rate of human bone marrow mesenchymal stem cells (hBMSCs) was assessed using the widely used CCK‐8 assay, with three replicates performed. f) The deposition of mineralized calcium during osteogenic differentiation has been assessed using Alizarin Red S getting stained at day 21 for the control group, the BGS group, and the TBGS group. The prevalence of red calcium nodules in the TBGS suggests that hBMSCs are capable of becoming osteoblasts in vitro. g) The effectiveness of BGS and TBGS for in vivo osteogenesis. (I,II) 24 weeks after scaffold implantation, 3D‐reconstructed circular cranial bone deficiencies. (III‐VI) Micro‐CT pictures of 5 mm‐diameter cranial defect locations at week 24 following surgery. VII) The neonatal osseous tissue volume percentage to the total volume of the defect (BV/TV) (n equals 6). Figures VIII and XI display the measurements of BMD and total porosity (TOT) in newborn osseous tissue, with a sample size of six (*n* = 6). *p < 0.05, **p < 0.01, ***p < 0.001. Reproduced with permission.^[^ ^171^ ^]^ Copyright 2020, Wiley‐VCH. Copyright pending.

Pan et al.^[^ ^171^ ^]^ devised biomaterials with various uses for the simultaneous obliteration of bone tumor cells^[^ ^172^ ^]^ and the bone cell regeneration by assimilating Ti_3_C_2_ MXenes^[^ ^74^, ^173^, ^174^ ^]^ into 3D‐printed bioactive glass scaffolds (TBGS).^[^ ^173^, ^175^, ^176^ ^]^ The initial large‐scale Ti_3_AlC_2_ ceramics were subjected to HF etching, and then exfoliated using tetrapropylammonium hydroxide (TPAOH) to produce these 2D ultrathin Ti_3_C_2_ NSs. BGSs were incorporated with Ti_3_C_2_ NSs at high initial concentrations to produce a suitable amount for photothermal ablation (**Figure** **24**a,b,d). The viability of cells in the laser only, BGS, BGS + laser, and TBGS groups ranged from ≈90 to 100%, exhibiting no noticeable difference compared to the control group (blank) showing the produced TBGSs’ outstanding biocompatibility (Figure 24c). Particularly, the cells on BGSs had significantly lesser filopodia than the 2hBMSCs^[^ ^177^ ^]^ on TBGSs. TBGSs also were more biocompatible and proficient in facilitating cell growth, as quantified by the standard CCK‐8 assay (Figure 24e). Alizarin red assay^[^ ^178^ ^]^ was utilized to evaluate the extracellular matrix mineralization of hBMSCs in the TBGSs, BGSs, and control groups. The findings show the presence of significantly more calcium nodules in the TBGS group, revealing that TBGS augmented the osteogenic capacity of hBMSCs in vitro (in Figure 24f). Moreover, the biodegradation of Ti_3_C_2_ MXenes into bioactive minerals such as PO_4_^3+^ and Ca^2+^ resulted in the formation of Ti‐based species, which facilitated the acceleration of new bone formation. After implantation for 24 weeks, microcomputed tomography (micro‐CT) analysis of the posterior and anterior aspects of the skull demonstrated that the TBGS exhibited a more effective bone regeneration effect in comparison to the bioactive glass (BGS) without MXenes (Figure 24g(I–VI)).^[^ ^64^ ^]^ The exceptional osteogenic efficiency of TBGS is highlighted by an elevated level of bone mineral density (BMD) and a greater proportion of newly formed bone cells to volume of the bone defect (BV/TV) in TBGS compared to BGS (Figure 24g (VII‐IX)). With the ability to both eradicate tumors and grow new bone cells, these composite scaffolds that contain Ti_3_C_2_ MXene have enormous potential to treat bone cancers. Additionally, this broadens the scope of 2‐dimensional MXenes’ tissue engineering applications in biomedicine.

**Figure 25:**
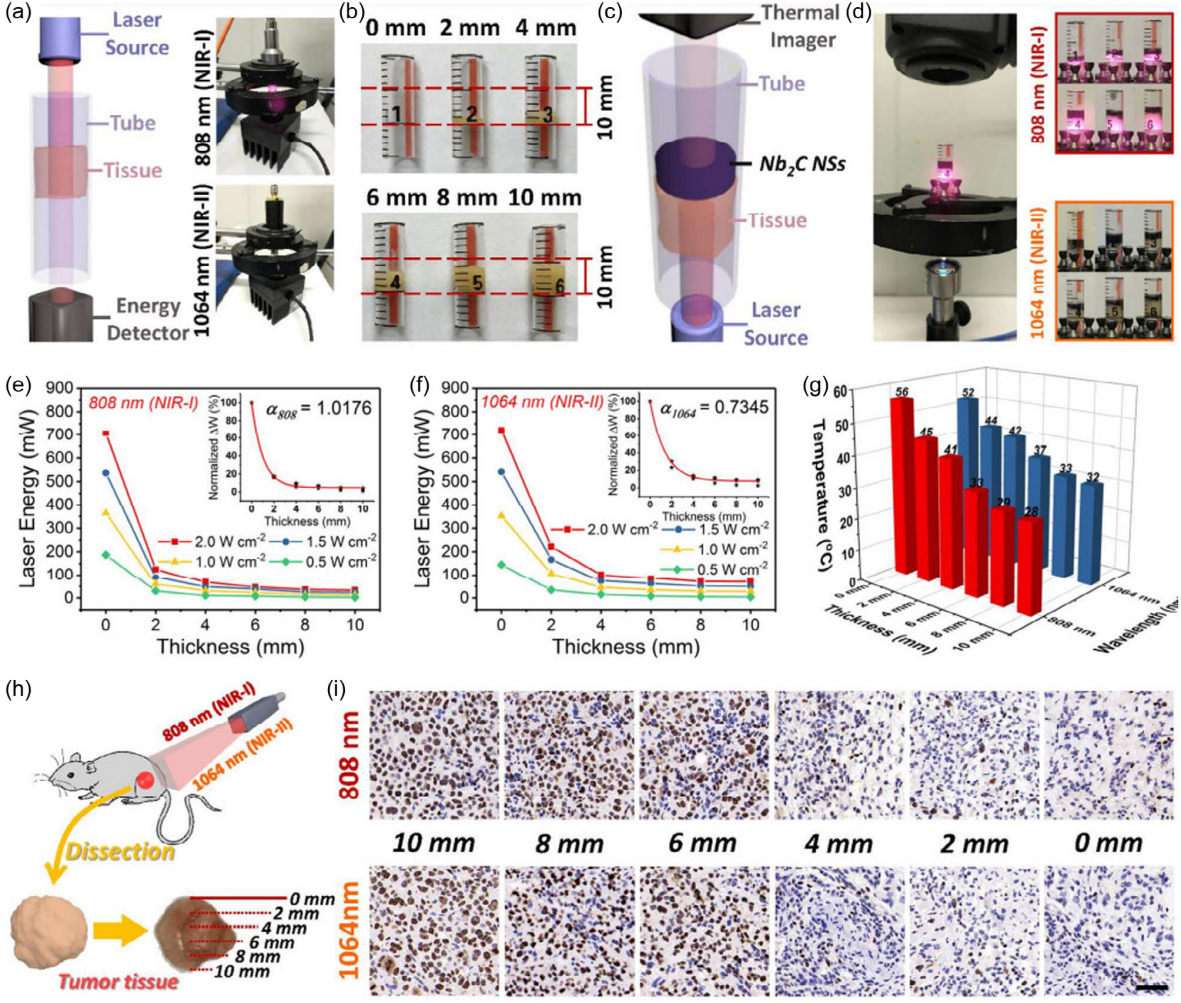
Assessment of In Vitro and In Vivo Tissue Penetration Depth for NIR‐I and NIR‐II Photothermal Conversion. a) A schematic representation and the experimental setup used to evaluate the tissue penetration capability of NIR lasers at 808 nm and 1064 nm. b) Chicken breast tissues of varying thicknesses (0, 2, 4, 6, 8, and 10 mm) placed in transparent tubes for analysis. c) Schematic illustration and d) corresponding equipment for demonstrating the photothermal conversion of Nb_2_C NSs aqueous suspensions upon exposure to tissue‐penetrated NIR laser (1.0 W cm^−2^, 80 μg mL^−1^). e) Energy intensities of NIR‐I (808 nm) laser transmitted through tissues of different thicknesses, with an inset showing normalized penetrated NIR‐I energy at varying depths. The attenuation coefficient (α808) for 808 nm NIR‐I laser is indicated. f) Energy intensities of NIR‐II (1064 nm) laser transmitted through tissues of varying thicknesses, with an inset displaying normalized penetrated NIR‐II energy at different depths. The attenuation coefficient (α1064) for 1064 nm NIR‐II laser is noted. g) Temperature increase in Nb_2_C NSs aqueous suspensions following exposure to tissue‐penetrated NIR‐I and NIR‐II lasers, demonstrating photothermal conversion. h) Schematic representation of in vivo tumor tissue penetration for photothermal conversion using NIR‐I and NIR‐II. i) Immunofluorescence staining of the Ki‐67 antigen to assess cellular proliferation at different depths (0, 2, 4, 6, 8, and 10 mm) within dissected tumor tissues. All images maintain a consistent scale bar of 50 μm. Reproduced with permission.^[^ ^74^ ^]^ Copyright 2017, ACS. Copyright pending.

Lin et al.^[^ ^74^ ^]^ showcased the remarkable effectiveness of 2‐dimensional niobium carbide (Nb_2_C) MXene in vivo for the eradication of mouse cancer grafts using photothermal ablation, employing NIR‐I as well as NIR‐II wavelengths. MXene was produced employing a simple and extensible liquid exfoliation method involving two sequential steps that combines sequential layer separation and insertion processes. To assess the depth of tissue penetration within the NIR‐I and NIR‐II biowindows, the measurement of laser energy intensity was performed following irradiation of chicken breast muscles. These muscle samples served as models for biological tissues (**Figure** **25**a,b). The study assessed the effectiveness of Nb_2_C NSs in harnessing deep‐tissue photothermal energy within the NIR‐I and NIR‐II ranges. This evaluation involved exposing chicken breast muscle to laser irradiation (see Figure 25c). An infrared camera was used for recording the infrared thermographs following 5 min of 808 and 1064 nm laser irradiation (at a value of 1 W cm^−2^) on 100 L of aq. solutions of dispersed Nb_2_C NSs (see Figure 25d). When the energy remains of the NIR‐I and NIR‐II lasers were evaluated after passing via the tissues with varied thickness, the NIR‐I laser with a value of 808 nanometers showed a greater absorption propensity than the 1064 nm NIR‐II laser. (Figure 25e,f). The calculated attenuation coefficients for the NIR‐I and NIR‐II fitting curves for 808 and 1064 nm lasers, respectively, are 1.02 and 0.73 (Figure 25e,f, inset). These values show the possibility to efficiently heat the Nb_2_C NSs solution without significantly raising the temperature of the cell. The temperature changes observed in different tissue layers when exposed to NIR‐I and NIR‐II laser radiation tell about the NIR‐II laser causing less reduction in photothermal heating in comparison to the NIR‐I laser when passed via tissues of similar thickness (Figure 25g).

To establish the optimal depth of tissue penetration for photothermal ablation using NIR‐I and NIR‐II, mice with 4T1 tumor grafts were exposed to laser irradiation at wavelengths of 808 or 1064 nm. The outcomes of these experiments are illustrated in Figure 25h. Posttreatments, the tumor cells were divided to examine the levels to which the cancerous tissue was growing cells. This showed that a subcutaneous xenograft tumor could be effectively photothermally abated in nude mice at a depth of 4 mm (Figure 25i). Utilizing Nb_2_C‐PVP NSs for thermal therapy that penetrates deep tissue in biological windows of NIR‐I and NIR‐II, has a large potential, as shown by the calculation by Bashkatov et al.^[^ ^179^ ^]^

**Figure 26:**
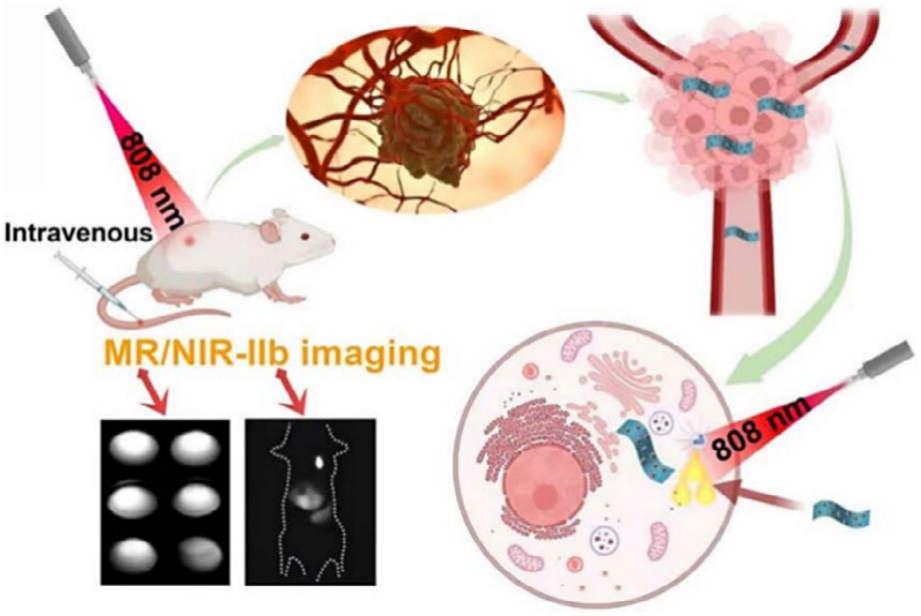
Schematic diagram illustrating the fabrication process of NaErF_4_@Ti_3_C_2_ nanocomposites for NIR‐II fluorescence imaging‐guided PTT in tumor treatment. Reproduced with permission.^[^ ^180^ ^]^ Copyright 2022, ACS. Copyright pending.

Pan et al.^[^ ^180^ ^]^ created a MXene wherein NaErF4:0.5%Tm@ NaLuF4^[^ ^181^, ^182^ ^]^ NPs had been loaded over the Ti_3_C_2_ NSs surface for PTT guided by NIR‐IIb (1530 nm) and MRI. To create the ultrathin Ti_3_C_2_ MXene NSs, a 2‐step exfoliation procedure was adopted. As a result, nanosized MXene NSs with appropriate planar size and dispersity were produced, in line with the criteria of biomedical research. A conventional solvothermal approach was used to create NaErF4:0.5%Tm@NaLuF4 NPs. The NaErF4:0.5%Tm@NaLuF4 NPs' initial OA ligands were substituted with HCl, which caused the NPs to move from oil to the aqueous phase and, after that, combine with Ti_3_C_2_ NSs to produce NaErF4@Ti_3_C_2_ nanocomposites through electrostatic interactions (**Figure** **26**A). The in vitro T2‐weighted NaErF4@Ti_3_C_2_'s MRI performance at various doses was examined to assess the capacity of NaErF4@Ti_3_C_2_ to be an efficient MRI contrast agent. As the concentration of NaErF4@Ti_3_C_2_ composites rises, the pattern, which depicts the strength of the MR signal, becomes darker. These results imply the possibility of NaErF4@Ti_3_C_2_ nanocomposites serving as effective contrast agents in high‐field T2‐weighted MRI. When excited by an 808 nm laser, the NIR‐IIb signals (1500 nm long pass (LP)) were sufficiently bright for passing through the 3–5 mm deep tissue, enabling high‐contrast and high‐resolution imaging. The liver and spleen displayed legible light signals within 1 min of injection. The EPR effect led to 1525 nm luminous signals appearing at the tumor location after about 45 min, which indicates that the nanocomposites had begun concentrating in the tumor (Figure 26B). The imaging data was used to usher subsequent photothermal treatment. Based on their great photothermal conversion capacity (equal to 43.62% at a value of 808 nm irradiation) and photothermal stability, the proposed composites had been studied more for efficacy with an inhibition ratio equal to 92.9%, which was proven both in vivo and in vitro systematically. Also, there was no detectable nanocomposite toxicity at the administered dose. This research demonstrated the potential of the various MXene‐based multifunctional nanomaterials applications in cancer therapy in addition to enhancing their effectiveness.

**Figure 27:**
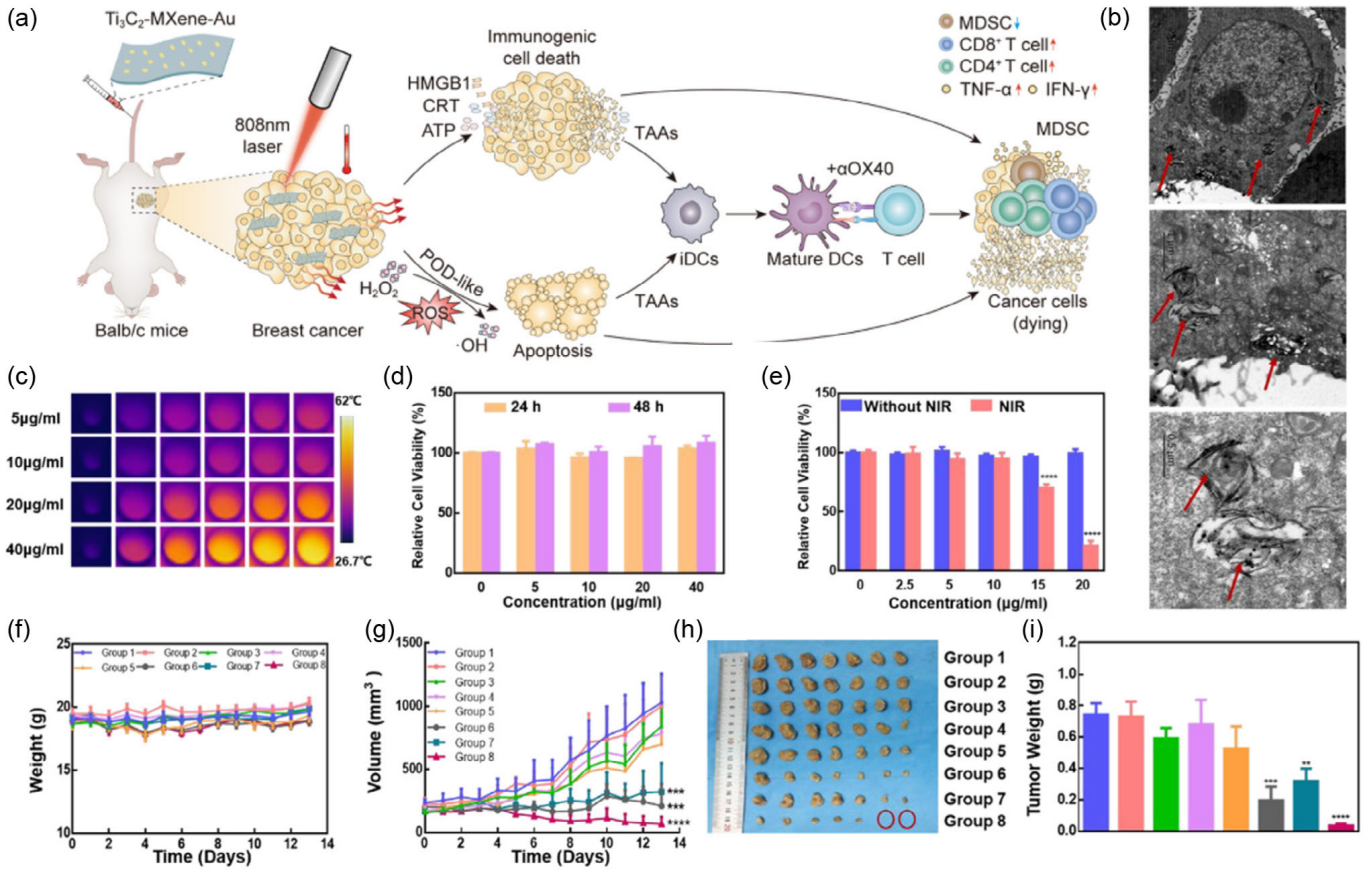
a) Mechanism of the PTT/EDT/immunotherapy triune Ti_3_C_2_‐MXene‐Au nanoplatform. b) TEM pictures of a tumor cell containing Ti_3_C_2_‐MXene‐Au. c) Ti_3_C_2_‐MXene‐Au thermal pictures at different concentrations after laser irradiation. d) The relative viability of MCF10A cells after being exposed to Ti_3_C_2_‐MXene‐Au nanocomposites. e) 4T1 cells relative viabilities exposed to laser irradiation and Ti_3_C_2_‐MXene‐Au. f, g) Body weights and tumor development in mice which have been implanted with the 4T1 tumor and given various treatments. h) On day 14, representative photographs of tumors in various groups were captured: group 1 – PBS, group 2 – Ti_3_C_2_‐MXene−Au, group 3 – αOX40, group 4 – Ti_3_C_2_‐MXene−Au + αOX40, group 5 – PBS + NIR, group 6 – Ti_3_C_2_‐MXene−Au + NIR, group 7 – αOX40 + NIR, and group 8 – Ti_3_C_2_‐MXene−Au + αOX40 + NIR. i) Measurement of tumor weight was performed on day 14 to quantify the tumor mass. Reproduced with permission from Ref.^[^ ^183^ ^]^, Copyright 2022, ACS. Copyright pending.

**Figure** **27**a depicts the scheme developed by Chang et al.^[^ ^183^ ^]^ which suggests combining Ti_3_C_2_‐MXene‐Au nanomedicine with OX40 mAb to create a triad of enzyme dynamic/photothermal/antitumor immune synergistic therapy for TNBC^[^ ^184^ ^]^ Ti_3_C_2_‐MXene‐Au nanocomposites were able to build up at the tumor tissue following biodistribution from IV administration and attained PA/thermal dual‐modality imaging in vivo in the interim, owing to the improved retention and permeability effect (EPR) for nanostructures. Laser irradiation would cause localized heating at the tumor tissue for thermal ablation and would expedite the catalysis reaction to yield hydroxyl radicals (⋅OH), which would cause cancer cell cells to undergo necrosis and apoptosis. Additional administration of OX40 mAb enhanced T cell antitumor immunoreactivity and alleviated tumor cell‐mediated immunosuppression. As a result, a safe and dynamic Ti_3_C_2_‐MXene‐Au system was developed that facilitates NIR‐triggered simultaneous EDT, PTT, and immunotherapy to eliminate cancer cells. Figure 27b displays TEM images revealing a slight bending of Ti_3_C_2_‐MXene Au, characterized by higher electron density, within the cytoplasm. This indicates that Ti_3_C_2_‐MXene Au can easily penetrate and accumulate in tumor cells. By examining 4T1 cells subjected to different treatments, DAMPs expression having CRT, HMGB1, and ATP, which are essential for dendritic cell (DC) maturation and subsequent activation of immunogenic cell death (ICD)‐induced antitumor immunity, could be detected.^[^ ^185^, ^186^, ^187^, ^188^ ^]^ In comparison to other groups treated, the Ti_3_C_2_‐MXene‐Au + NIR group showed a greater level of HMGB1 discharge from 4T1 cells and stronger fluorescence intensity for CRT. Figure 27c shows that at a conc. of 40 μg mL^−1^, the Ti_3_C_2_‐MXene−Au exhibited a substantial temperature rise from 26.7 to 62 °C when exposed to laser power density equal to 1.5 W cm^−2^ for a time period of 5 min. In contrast, the change in temperature of DI water was ≈5.6 °C, which aligned with the results obtained from the infrared thermal camera measurements. MCF‐10 A cells have been given an exposure to the nanocomposites for 24 and 48 h, and no toxicity was observed according to CCK‐8 detection, depicted in Figure 27d, indicating that the nanocomposites were safe for use. When irradiated with laser, the Ti_3_C_2_‐MXene−Au nanocomposites exhibited toxicity to 4T1 and MDA‐MB‐231 cells which was found to be concentration dependent, as depicted in Figure 27e. Anticancer characteristic of combined PTT/EDT treatment was showcased by annihilation of ≈80% of 4T1 cells at 20 μg mL^−1^ concentration. Figure 27f shows infinitesimal variations in the mice weights across all groups. To estimate the therapeutic action, the volume and weight of tumors of every group were measured in Figure 27g,i. Photographs of the pertaining tumor tissues are depicted in Figure 27h.

## 5 Conclusion

As a result of their distinctive physical and chemical characteristics, MXenes have developed into a new subclass of 2D materials that show promise for application in various biomedical usages. The review study examines the difficulties related to their stability in physiological settings, prolonged drug release, and biodegradability and highlights recent advancements in MXene synthesis using top‐down and bottom‐up methods. MXene‐based composites were studied for their potential in the therapy of cancer, specifically in PDT and PTT. The review paper presents an overview of MXene‐based composites for PTT and PDT, such as their mechanism of action, advantages, and limitations. The MXene‐based composites development has opened up new opportunities for designing and developing novel therapeutic strategies for cancer treatment. In summary, this article provides insight into MXene's current status in biomedical applications and highlights its potential as a promising material for future biomedical research.

## Conflict of Interest

The authors declare no conflict of interest.

## Author Contributions

**Aryan Saxena**: conceptualization (lead); writing—original draft (lead); writing—review & editing (lead). **Akshayat Tyagi**: conceptualization (equal); formal analysis (equal); methodology (equal); writing—original draft (equal); writing—review & editing (equal). **Sushipra Vats**: conceptualization (equal); project administration (equal); validation (lead); writing—review & editing (lead). **Ishita Gupta**: conceptualization (equal); methodology (equal); writing—original draft (equal); writing—review & editing (equal). **Akhil Gupta**: conceptualization (equal); formal analysis (equal); writing—original draft (equal); writing—review & editing (equal). **Raminder Kaur**: project administration (equal); writing—review & editing (equal). **Saurabh Kr Tiwary**: project administration (equal); resources (equal); supervision (equal); writing—review & editing (equal). **Ahmed Elzatahry**: data curation (equal); project administration (equal); supervision (equal); validation (equal); writing—review & editing (supporting). **Maninderjeet Singh**: supervision (supporting); writing—review & editing (supporting). **Alamgir Karim**: supervision (lead); writing—review & editing (lead).
